# Neuronal protein Sex-lethal modulates tRNA synthesis via the polymerase III subunit Polr3E in male *Drosophila* neurons

**DOI:** 10.1371/journal.pbio.3003863

**Published:** 2026-07-17

**Authors:** Freya Storer, Colin D. McClure, Alicia Estacio Gomez, Lucy J. Minkley, Tsz Lam Wong, Nina Markevych, Tony D. Southall

**Affiliations:** 1 Department of Life Sciences, Imperial College London, Sir Ernst Chain Building, London, United Kingdom; 2 School of Life Sciences, University of Nottingham, Queen’s Medical Centre, Nottingham, United Kingdom; University of Lausanne, SWITZERLAND

## Abstract

The RNA-binding protein Sex-lethal (Sxl) is classically known as a master regulator of sex determination and mRNA splicing in *Drosophila melanogaster*. However, this role is not conserved across species, and functions beyond the canonical pathway remain poorly understood. In this study, we uncover a splicing-independent role for Sxl at the chromatin level in the *Drosophila* brain. Using Targeted DamID (TaDa) profiling in neurons, we identify widespread binding of Sxl to promoter regions, independent of sex or RNA binding activity. Notably, Sxl chromatin occupancy exhibits near-complete overlap with Polr3E (RPC37), an RNA Polymerase III subunit, with Sxl binding abolished upon *Polr3E* knockdown. Depletion of Sxl in mature male neurons induces widespread transcriptional changes, particularly in metabolic genes, and improves negative geotaxis during aging, phenotypes that closely mirror *Polr3E* knockdown. Conversely, overexpression of the brain-specific *Sxl*^*RAC*^ transcript leads to severe climbing deficits and upregulated gene expression associated with metabolism and translation. Manipulating *Sxl* levels in the brain significantly impacts select tRNA production and global protein synthesis rates. Together, these findings reveal a previously unrecognized role for Sxl in regulating Pol III activity via Polr3E, modulating tRNA synthesis and supporting neuronal metabolism. Given the emerging tie between Pol III regulation and neuronal aging, our study highlights Sxl as a novel factor in neuronal homeostasis.

## Abstract 2

The RNA-binding protein Sex-lethal regulates sex determination and mRNA splicing in Drosophila. This study reveals a new role in neuronal homeostasis and transfer RNA synthesis via Polr3E regulation in neurons.

## Introduction

**Fig 1:**
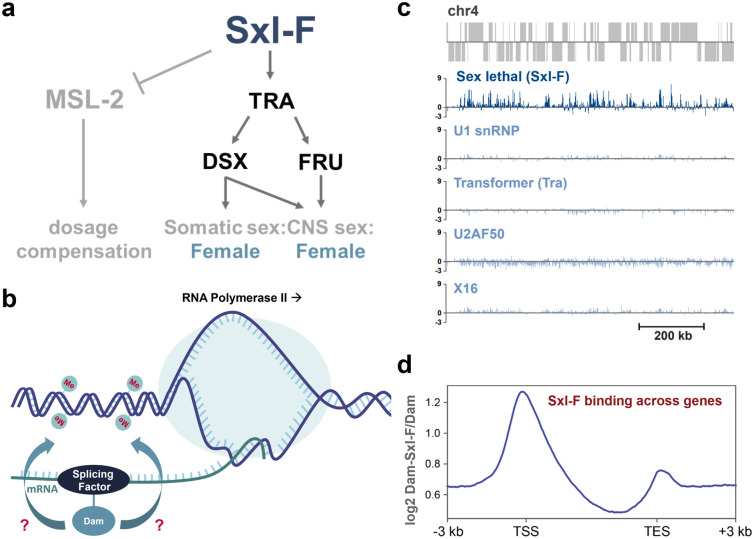
Sxl-F robustly associates with chromatin while other splicing factors do not. **a,** Overview of Sxl-F function in sex determination in *Drosophila melanogaster* (adapted from [6]). **b,** Schematic outlining the use of Targeted DamID (TaDa) to test whether splicing factors make transient contacts with chromatin. **c,** TaDa profiles for Sxl-F and four additional splicing factors across chromosome 4 in neurons of *Drosophila* larval brains. The *y*-axis represents the log_2_ ratio of Dam-factor over Dam-alone. **d,** Metagene plot showing average Sxl-F binding across gene bodies aligned to transcription start sites (TSS) and transcription end sites (TES). Data underlying this figure are available in S1 Table and S1 Fig.

Sexual dimorphism in *Drosophila melanogaster* is tightly controlled by one of the most established pathways in insect biology. At its core lies Sex lethal (Sxl), a splicing factor and master regulator that acts as a binary switch, controlling the sexual identity of the organism. In early female embryos, the ratio of X-linked signaling elements activates Sxl’s ‘early’ promoter, initiating expression of the *Sxl*^*early*^ transcript [1]. Sxl^early^ protein, once translated, directs the female-specific splicing of the *Sxl*^*late*^ transcripts, which are constitutively transcribed [2,3]. This autoregulatory splicing ensures the continued production of the functional canonical Sxl protein in females (Sxl-Female (Sxl-F)) [4], maintaining its own expression and initiating the downstream female-specific sex determination cascade [5]. In males, where *Sxl*^*early*^ is not produced, *Sxl*^*late*^ undergoes default splicing, generating a truncated protein with no known function. This binary mechanism ensures a stable and heritable cellular memory of sexual identity throughout development (Fig 1a).

Although essential for sexual differentiation, the function of Sxl-F has long been considered limited to a number of well-defined roles. Its primary function is to regulate the female-specific splicing of *transformer (tra)* pre-mRNA, a key step in the sex determination hierarchy [7]. Sxl-F promotes exon skipping in *tra*, preventing premature translation termination and enabling the production of functional Tra protein. In turn, Tra drives feminization of tissues through the alternative splicing of transcription factor genes *Doublesex* (*Dsx*) and *Fruitless* (*Fru*) by producing female-specific isoforms of each protein [6]. These factors drive transcriptional changes that lead to the majority of sexual dimorphic physiology and behavior in *Drosophila*. In addition to its role in splicing regulation, Sxl-F inhibits translation of *Male-Sex-lethal 2* (*msl-2*), thereby preventing assembly of the dosage compensation complex (DCC), which otherwise upregulates X-linked gene expression in males [8,9].

Sxl-F contains two RNA-Binding Domains (RBDs), each accommodating two highly conserved ribonucleoprotein (RNP) motifs that recognize poly(U) sequences to regulate splicing [10]. In addition to RNA recognition, these domains mediate interactions with other proteins, including splicing factor Snf16 and the RNA polymerase III subunit E (Polr3E) [11]. Beyond the RBDs, Sxl-F possesses a 120-residue N-terminal domain and an 80-residue C-terminal domain. The first 40 amino acids of the N-terminus are critical for cooperative RNA binding [Wang and Bell, 1996]. Loss of this region uncouples Sxl-F’s sex determination and dosage compensation functions, whereby the protein retains its ability to repress *msl-2* translation but fails to promote female-specific splicing of *tra* [12].

An intriguing aspect of Sxl biology is the expression of another truncated isoform, Sxl^RAC^, in the male nervous system [4,13,14]. Unlike the canonical female-specific isoforms, Sxl^RAC^ is expressed in both sexes but appears restricted to the nervous system of male pupae and adults [13,14]. Notably, Sxl^RAC^ lacks the first 23 amino acids of the N-terminal region, an area critical for promoting female-specific splicing of *tra* [12,15]. Consistent with this truncation, full-length Tra protein is absent in males [7], suggesting that Sxl^RAC^ functions independently of its canonical role in alternative splicing.

Despite extensive characterization of Sxl-F as the master regulator of sex determination, emerging evidence points to additional, Tra-independent functions. A phenomenon termed Tra-insufficient Feminization (TIF) highlights physiological and transcriptional effects that cannot be explained by the canonical Sxl-Tra-Dsx/Fru axis [16]. TIF includes (i) partial feminization of *Sxl* mutant lines rescued by Tra2, (ii) transcriptional changes independent of Dsx and Fru [17], and (iii) phenotypes following *Sxl* knockdown that are not replicated by *tra* depletion [18]. Collectively, these findings support the existence of an alternative, yet unidentified, mode of Sxl action beyond its canonical role in sex-specific splicing.

## Results

### Sxl robustly associates with chromatin while other splicing factors do not

The majority of mRNA splicing occurs co-transcriptionally, bringing factors into close proximity with DNA [19]. To investigate whether splicing factors transiently associate with chromatin in vivo, we employed Targeted DamID (TaDa), a cell-type-specific method for profiling protein–DNA interactions without the need for antibodies or cell isolation [20]. In this system, the protein of interest is fused to the *E. coli* DNA adenine methyltransferase (Dam), which deposits methylation marks at nearby GATC motifs, enabling genome-wide mapping of protein occupancy. Towards this, splicing factors were tagged with Dam to assess whether this enabled profiling of their transient interactions with chromatin in neurons of the developing larval brain (Fig 1b). Dam fusions to U1, Tra, U2AF50 and x16 yielded no significant chromatin-associated signal (Fig 1c), suggesting limited or highly transient interactions. In contrast, Dam fused to Sxl-F exhibited robust chromatin binding, with marked enrichment at transcriptional start sites (TSS) (Fig 1d). Notably, Sxl-F binding encompassed a substantial proportion of the genome, with occupancy detected at 53% of expressed genes (S1 Fig and S1 Table). This widespread chromatin occupancy suggests that Sxl-F may have a broader role in transcriptional regulation beyond its established function in alternative splicing.

### Sxl-F associates with chromatin independently of sex and RNA-binding ability

**Fig 2:**
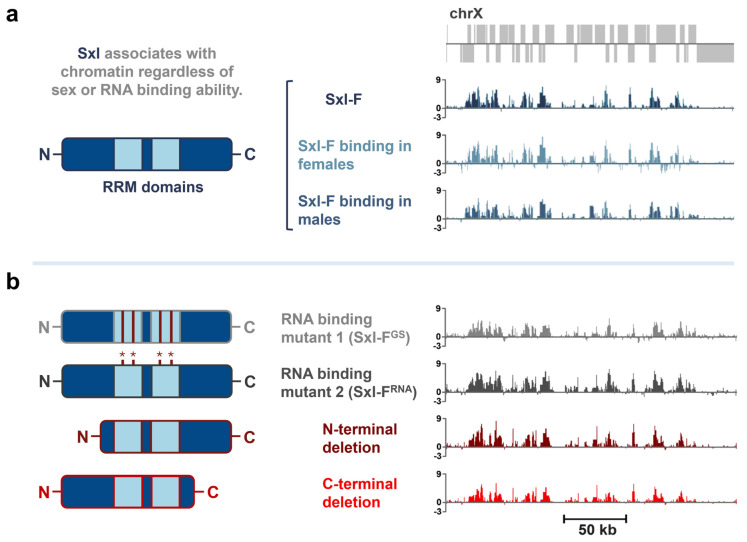
Sxl-F associates with chromatin independently of sex and RNA-binding ability. **a,** Targeted DamID profiles from *Drosophila* larvae showing chromatin occupancy of wild-type Sxl-F in mixed-sex samples (dark blue), females (light blue), and males (medium blue). **b,** Dam-fused Sxl-F variants carrying disruptions in RNA-binding capacity or domain deletions analyzed in mixed-sex larvae. Light blue regions denote RNA recognition motifs (RRMs). Red lines indicate positions at which ribonucleoprotein (RNP) sites were replaced with glycine-serine (GS) linkers. Asterisks mark individual point mutations (Y168A, F170D, V254A, F256D). Data underlying this figure are available in S2–S5 Tables.

Sxl-F, which mediates alternative splicing of *tra* pre-mRNA, is expressed in females but absent in males. To determine whether sex-specific factors influence Sxl-F’s chromatin binding, we performed TaDa profiling of Sxl-F in the neurons of both male and female animals. Interestingly, no significant differences in binding were observed, suggesting a role besides sex determination (Fig 2a). Going deeper, we explored whether Sxl-F’s chromatin association was due to its interaction with RNA substrates, whereby two mutant versions of Sxl-F were generated, incapable of binding RNA. The first mutant (*Sxl-F*^*GS*^) replaces the RNA recognition motifs (RNPs) with glycine/serine linkers [10], while the second mutant (*Sxl-F*^*RNA*^) carries mutations in key amino acids within the RNPs (Y168A, F170D, V254A, F256D) [21]. Previous studies have shown that similar mutations in other RNA-binding proteins, such as *Drosophila* ELAV, abolish RNA-binding ability [21]. Next, the chromatin binding function of these mutants was assessed using TaDa and strikingly, both Dam-fused Sxl-F^GS^ and Sxl-F^RNA^ were able to bind chromatin, as did mutants lacking either the N- or C- terminal regions (Fig 2b). These results suggest that a cofactor, likely interacting with Sxl-F’s central region (containing RNA-binding domains), may mediate Sxl’s recruitment to chromatin. A strong candidate for this interaction is the Pol III subunit Polr3E, previously shown to bind Sxl at RNA-binding domain-1 (RBD-1) in a yeast two-hybrid screen [11].

### Polr3E is required for Sxl-F’s association with chromatin

**Fig 3:**
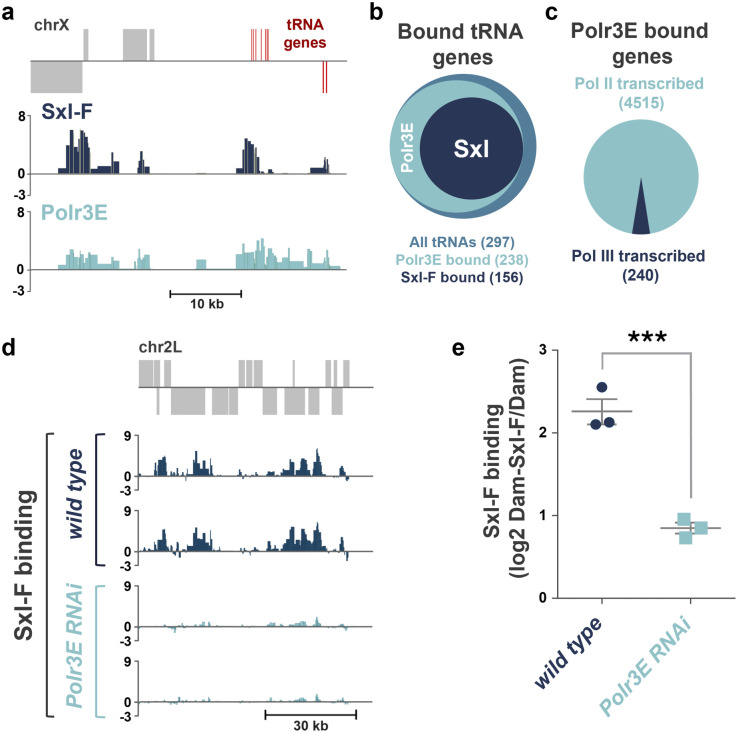
Polr3E is required for Sxl-F’s association with chromatin. **a,** Genome-wide binding profiles of Polr3E show strong overlap with Sxl-F chromatin occupancy, as assessed by targeted DamID (TaDa). **b,** Polr3E binds the majority of annotated tRNA genes, with Sxl-F binding a defined subset. **c,** Polr3E is enriched at both RNA polymerase II and RNA polymerase III-transcribed loci. **d,** Chromatin binding of Sxl-F is markedly reduced in neurons depleted of *Polr3E* by RNAi. **e,** Quantification reveals a significant reduction in Sxl-F binding following *Polr3E* knockdown (***p = 0.0009, unpaired *t* test). Data underlying this figure are available in S6–S8 Tables.

To assess whether Polr3E mediates Sxl association to chromatin, Polr3E binding across the genome in larval neurons was profiled using TaDa. Remarkably, Polr3E shared a highly similar binding profile with Sxl-F (Fig 3a, S6 Table). Both proteins bound to a significant number of Pol III-transcribed genes, including tRNA genes (Fig 3b and S7 Table), but also a large subset of Pol II-transcribed genes (Fig 3c). This observation is not unprecedented, as *Drosophila* TFIIIC, a component of the Pol III transcriptional machinery, also associates with many Pol II-transcribed genes (~2,400) [22].

To directly test whether Polr3E was required for Sxl-F binding, *Polr3E* was knocked down in larval neurons while simultaneously profiling Sxl-F binding via TaDa. This resulted in a marked reduction in Sxl-F occupancy across target sites (*p* = 0.0009, unpaired *t* test; Fig 3d and 3e and S8 Table), confirming that Polr3E is required for Sxl-F recruitment to chromatin.

These results establish Polr3E as a key determinant of Sxl-F chromatin association and highlight the potential for Polr3E to coordinate Sxl-F’s influence on both Pol III- and Pol II-dependent transcriptional programs or vice versa.

### *Sxl* knockdown phenotypes present similarly to *Polr3E* loss in adult males

**Fig 4:**
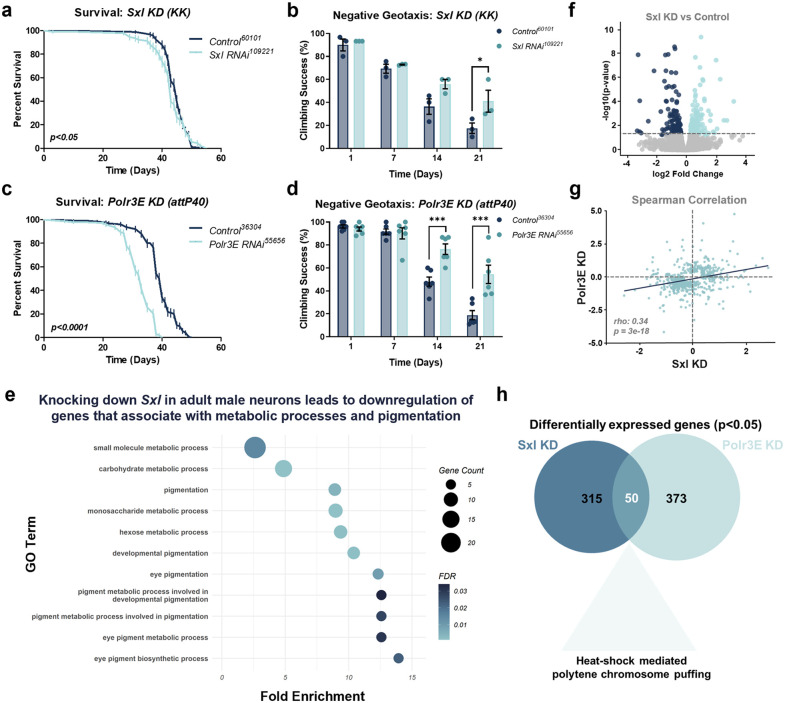
*Sxl* knockdown phenotypes present similarly to *Polr3E* loss in adult males. **a,** Survival curves of adult males following pan-neuronal knockdown of *Sxl* (VDRC #109221) compared to RNAi controls (VDRC #60101) (*n* > 90 per group). Statistical significance was assessed using the Gehan–Breslow–Wilcoxon and Log-rank tests. **b,** Negative geotaxis assay showing improved climbing ability following *Sxl* knockdown in male neurons. The *y*-axis (Climbing Success) indicates the percentage of flies surpassing the 4-cm midpoint. Data were analyzed by two-way ANOVA with Bonferroni post-hoc test; *p < 0.05. **c,** Survival curves following neuronal knockdown of *Polr3E* (BDSC #55656) compared to RNAi controls (BDSC #36304) (*n* > 120 per group). Statistical significance was assessed using the Gehan-Breslow-Wilcoxon and Log-rank tests. **d,** Improved climbing performance in *Polr3E* knockdown flies after day 14 post-eclosion; ***p < 0.001, mirroring the *Sxl* knockdown phenotype. **e,** Gene ontology enrichment analysis of transcripts downregulated after *Sxl* knockdown in adult male neurons. Dot plot shows the top 10 GO terms ranked by fold enrichment (*x*-axis); dot size indicates the number of gene hits, and color reflects false discovery rate (FDR). **f,** Cropped volcano plot showing differential gene expression following *Sxl* knockdown in adult male neurons relative to controls (full size in S3 and S10 Figs). Significantly upregulated transcripts (*p* < 0.05) are shown in light blue, and significantly downregulated transcripts (*p* < 0.05) in dark blue. **g,** Scatter plot comparing significant gene expression changes between *Polr3E* knockdowns and *Sxl* knockdown samples. Each point represents a gene locus, plotted by log_2_ fold change (LFC) in *Sxl* knockdowns (*x*-axis) vs. *Polr3E* knockdowns (*y*-axis). Solid line shows the line of best fit, dashed lines at zero define quadrants of concordant (upper right/lower left) and discordant (upper left/lower right) expression changes. Spearman correlation indicates a moderate positive association (*ρ* = 0.34, *p =* 2.996e−18). **h,** Genes differentially expressed (both directions) in either knockdown condition, overlapping primarily with transcripts associated with polytene chromosome puffing. Data underlying this figure are available in S9–S18, Tables and S2, S3, S10, and S11 Figs.

To further investigate Sxl’s role in the *Drosophila* nervous system, its expression and functional impact was explored in adult male neurons. This approach enabled the isolation of sex-independent functions, avoiding confounding effects related to its canonical role in female sex determination. Using a CRISPR knock-in line (*Sxl-T2A-GAL4*) [14] that traps the majority of Sxl isoforms, we observed that Sxl^RAC^ (the only protein isoform expressed in the male brain) is broadly distributed in neurons throughout the tissue, in contrast to its limited pattern during larval stages (S2 Fig). To investigate a functional role of Sxl^RAC^, expression was knocked down in adult neurons using RNAi driven by *nSyb-*GAL4, bypassing developmental effects using *tub-*GAL80^ts^. Depletion resulted in a mild but consistent reduction in survival (*p* < 0.05, Gehan-Breslow-Wilcoxon test; Figs 4a and S3) and age-related improvement of climbing ability after 21 days (*p* < 0.05, two-way ANOVA; Fig 4b). Strikingly, knockdown of *Polr3E* produced comparable phenotypes, including reduced survival (*p* < 0.0001, Gehan–Breslow–Wilcoxon test; Fig 4c) and climbing improvement after day 14 (*p* < 0.001, two-way ANOVA; Fig 4d).

Given previous evidence linking Pol III modulation to age-related phenotypes [23,24], these findings point to a potential role for Sxl^RAC^ in similar pathways. Exploring this further, RNA-seq was performed on 4-day-old male heads following *Sxl* knockdown. A significant downregulation of genes involved in metabolic processes and pigmentation was observed (Fig 4e and 4f), with no clear differences in pathways associated with sex-determination or dosage compensation (S13 and S14 Tables). Knocking down *Polr3E* showed similar transcriptional changes to the *Sxl* knockdown experiment, with a moderate positive correlation (Spearman *ρ* = 0.34, *p* < 0.0001, Spearman Correlation Test) between the two datasets (Fig 4g).

Crucially, when exploring transcriptional changes of Dam-Sxl targets, it was noted that over 69% of targets were significantly downregulated in *Polr3E* knockdowns, reflecting over 65% targets downregulated in *Sxl* knockdowns (S3 Fig). Genes differentially expressed in both knockdown conditions were enriched for polytene chromosome puffing pathways, suggesting that Sxl-Polr3E complexes may preferentially act at highly transcribed, decondensed chromatin domains (Fig 4h). Together, these results identify a previously unrecognized role for *Sxl* in supporting metabolism and transcription, and promote a functional overlap with Polr3E in adult male neurons.

### Perturbing Sxl^RAC^ levels leads to changes in tRNA expression and protein synthesis

To elucidate the role of the head-specific isoform Sxl^RAC^ [13], we generated a *UAS-Sxl*^*RAC*^ transgenic line and expressed it in mature male neurons using *nSyb*-GAL4, in combination with *tub*-GAL80^ts^ to restrict expression to adulthood.

**Fig 5:**
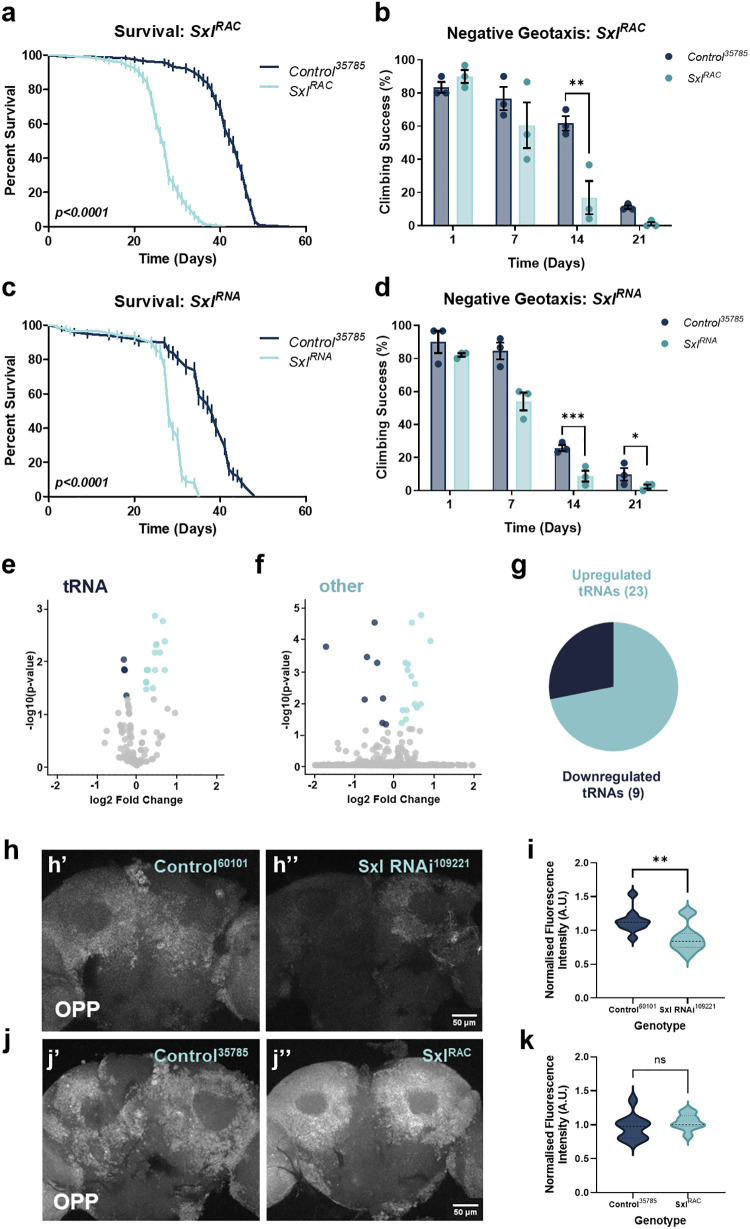
Perturbing Sxl^RAC^ levels leads to changes in tRNA expression and protein synthesis. **a,** Survival curves of adult males following pan-neuronal expression of *Sxl*^*RAC*^ compared to *mCherry* controls (*n* > 119 per group). Statistical significance was assessed using the Gehan–Breslow–Wilcoxon and Log-rank tests. **b,** Negative geotaxis assay showing reduced climbing ability following *Sxl*^*RAC*^ overexpression in male neurons. The *y*-axis (Climbing Success) represents the percentage of flies surpassing the 4-cm midpoint. Data were analyzed by two-way ANOVA with Bonferroni post hoc correction; **p < 0.01. **c,** Survival curves of adult males following pan-neuronal expression of *Sxl*^*RNA*^ compared with *mCherry* controls (*n* > 99 per group). Statistical significance was assessed using the Gehan–Breslow–Wilcoxon and Log-rank tests. **d,** Negative geotaxis assay showing impaired climbing in males overexpressing *Sxl*^*RNA*^. The *y*-axis represents the percentage of flies surpassing the 4-cm midpoint. Data were analyzed by two-way ANOVA with Bonferroni post hoc correction; *p < 0.05 and ***p < 0.001. **e,** Cropped volcano plot showing differential expression of tRNAs in adult male heads comparing *Sxl*^*RAC*^-expressing samples with controls (full size in S12 Fig). Significantly upregulated tRNAs (*p* < 0.05) are shown in light blue, and significantly downregulated tRNAs (*p* < 0.05) in dark blue. **f,** “Other” indicates additional classes of small RNAs, including rRNA, miRNA, ncRNA, snoRNA, and snRNA. The plot is cropped for clarity (full sizes in S13–S17 Figs). **g,** Pie chart illustrating that the majority (72%) of changes in tRNA abundance are due to upregulation. **h,** Immunolabeled z-stacks of adult central brains from 14-day-old male flies showing widespread OPP signal in controls (h′) and reduced OPP signal following neuronal *Sxl* knockdown (h″). **i,** Quantification of normalized OPP fluorescence intensity reveals a significant reduction following *Sxl* depletion (*p* = 0.0048, unpaired *t* test, *n* = 10–12 brains). **j,** Immunolabeled z-stacks of adult central brains from 14-day-old male flies displaying OPP signal in neurons expressing *Sxl*^*RAC*^ (j″) compared with con*t*rols (j′). **k,** Quantification of normalized OPP fluorescence intensity reveals no significant changes following *Sxl*^*RAC*^ expression (*p* = 0.28, unpaired *t* test, *n* = 9-11 brains).

Neuronal overexpression of *Sxl*^*RAC*^ significantly reduced survival compared to controls (*p* < 0.0001, Gehan–Breslow–Wilcoxon test; Fig 5a) and impaired age-dependent climbing ability (*p* < 0.01, two-way ANOVA; Fig 5b), contrasting with the phenotypes observed upon *Sxl* knockdown (Fig 4). RNA-seq analysis revealed widespread transcriptional changes, with significant upregulation of genes involved in metabolism and translation (S4 Fig). Expression of our RNA-binding-deficient construct (*UAS-Sxl*^*RNA*^) largely recapitulated these phenotypes (Figs 5c, 5d and S5), indicating that RNA-binding activity is dispensable for these outcomes. Consistently, ectopic expression of *Sxl*^*RNA*^ in neuronal (*elav*-GAL4) or ubiquitous (*tub*-GAL4) backgrounds did not induce male lethality (S42 Table), further confirming loss of canonical RNA-binding function.

Given the phenotypic similarities between *Sxl* and *Polr3E* perturbation (Fig 4) and their shared chromatin localization at tRNA loci (Fig 3b), we next assessed the impact of *Sxl*^*RAC*^ overexpression on tRNA abundance. Small RNA sequencing revealed numerous changes in tRNA and other small RNA populations (Fig 5e and 5f), with a strong bias towards upregulation (72% of significantly affected tRNAs; Fig 5g). Lys-tRNAs were particularly enriched among upregulated species (S5 Fig), whereas a subset of Glu-tRNAs were selectively reduced (S21 Table).

Assessing RNA polymerase III output independently, we quantified precursor tRNAs using established qPCR assays targeting distinct pre-tRNAs [24–26]. This analysis similarly showed elevated precursor tRNA levels, particularly *pre-Ile-tRNA*^*TAT*^, relative to the Pol II-specific U3 transcript (S6 Fig), with reversed trends observed upon *Sxl* knockdown (S6 Fig).

We next asked whether changes in tRNA abundance are accompanied by alterations in protein synthesis. To this end, O-propargyl-puromycin (OPP) labeling was employed to visualize nascent polypeptide synthesis. *mCherry* expression under *Sxl-T2A-GAL4* control revealed enriched *Sxl* levels within discrete neuronal populations, including the mushroom bodies and medulla (S7 and S8 Figs). Colocalization with the OPP signal was moderate (*R*(total) = 0.55; *R*(coloc) = 0.39; S23 Table), although not complete, indicating spatially heterogeneous and context-dependent coupling.

Manipulation of Sxl expression revealed age-dependent changes in translation. In 4-day-old males, Sxl perturbation did not significantly alter OPP incorporation (S8 Fig). However, in 14-day-old males, neuronal knockdown of Sxl significantly reduced global protein synthesis rates (*p* = 0.0048, unpaired *t* test; Fig 5h and 5i). In contrast, SxlRAC overexpression produced a mild, non-significant increase in OPP signal (Fig 5j and 5k).

Together, these data identify Sxl^RAC^ as a potential modulator of neuronal protein synthesis and associated processes. Enrichment correlates with locally elevated translational activity within specialized neuronal domains, and while overexpression alone is insufficient to drive significant increases, Sxl is required to maintain normal translation levels.

Integrating these findings with the observed Sxl-Polr3E chromatin association and corresponding changes in tRNA abundance supports a model in which neuronal Sxl modulates Pol III-dependent transcriptional output. The selective impact on specific tRNA species, particularly Lys-tRNAs, together with reciprocal effects following *Sxl* knockdown and similarities to *Polr3E* depletion, suggests that Sxl acts as a context-dependent regulator of Pol III transcriptional programs in neurons.

All images were captured at 20× magnification; scale bar, 50 µm. Data underlying this figure are available in S19–S25, S32, and S33 Tables and S12–S17 Figs.

## Discussion

Sex-lethal (Sxl), a master regulator of sex determination in the fruit fly, is known to complete this function through RNA splicing. Our study uncovers a previously unrecognized role for Sxl beyond this well-studied mechanism. Using Targeted DamID, we demonstrate that Sxl is robustly associated to chromatin in the *Drosophila* brain, independent of RNA-binding and sex, and this association is mediated through its interaction with Polr3E. Functional analyses reveal that Sxl^RAC^ influences Pol III-driven transcription, particularly of tRNAs, and plays an important role in maintaining neuronal health. Knockdown and overexpression studies in adult male neurons expose novel, sex-independent functions for Sxl^RAC^, including regulation of metabolic gene expression, life span, and age-related motor performance. Together, these findings identify Sxl as a selective, chromatin-associated modulator of Pol III activity and neuronal homeostasis, warranting further investigation into its functional role.

### Sxl as a chromatin interactor

A key discovery from our research is the identification of Sxl’s chromatin association, with enriched binding at promoters and transcriptional start sites (Fig 1). Notably, this chromatin occupancy occurs independently of Sxl’s splicing activity and RNA-binding ability and is evident in both male and female animals (Fig 2). Although this finding is novel in *Drosophila melanogaster*, it is not without precedent; prior work in *Drosophila virilis* demonstrated Sxl-chromatin association in salivary glands using polytene chromosome spreads [27]. Similar to our observations, binding was detected in both sexes and across the genome. Our studies further suggest that Sxl is widely expressed during late-stage brain development, with distinct and context-dependent expression patterns in adult male tissue, implying a requirement for functionally specialized roles. Mutant analysis confirms that chromatin occupancy is independent of both the N- and C-terminus of Sxl (Fig 2), reinforcing its splicing-independent role and highlighting the importance of its RNA-binding domain, which likely mediates its interactions but not through RNA binding per se. These findings open new avenues for investigating the role of Sxl and other RNA-binding proteins moonlighting as chromatin interactors [28,29].

### RNA Polymerase III modulated by Sxl

In the case of Sxl, RBD-1 is responsible for its interaction with binding partners like Polr3E [11]. The Targeted DamID data presented here reveals a striking co-occupancy of Sxl and Polr3E, with Sxl binding enriched at tRNA loci as well as Pol II-specific genes (Fig 3). Indeed, knockdown of *Polr3E* leads to a loss of Sxl binding to chromatin, suggesting a cooperative mechanism. We speculate that this interaction not only facilitates Pol III transcription but may also influence chromatin architecture and RNA Pol II-driven transcription as observed with Pol III regulation in other organisms [30–33]. Although direct evidence of Polr3E function in *Drosophila* neurons is limited, recent studies indicate that inhibition of Pol III activity yields phenotypes analogous to those observed in our functional knockdown experiments [23,24].

These previous experiments demonstrated that perturbation of Pol III function, through either subunit manipulation or chemical inhibition, affects survival and, in the case of *Polr3D* knockout, delayed age-related loss in climbing ability [23,24]. We note that knocking down *Polr3E* drastically delays this climbing loss (Fig 4), also mirrored in *Sxl* knockdowns, supporting a hypothesis that Sxl and Polr3E work together to maintain neuronal function. Transcriptional analyses further reinforce this, with correlation tests revealing a significant relationship between the two datasets (Fig 4 and S3) and highlighting metabolism as a key downstream process.

RNA Pol III is expressed ubiquitously during development and in a cell-type-specific manner in adulthood, where its activity must be tightly regulated [34,35]. Traditionally, RNA Pol III has been considered a core enzyme responsible for transcribing small, essential non-coding RNAs like tRNAs, 5S rRNA, and U6 snRNA, primarily to support basal cellular function. However, recent evidence indicates that Pol III activity is often elevated or specialized, reflecting the metabolic and transcriptional demands of highly active cells, particularly neurons [36,37]. This may underlie Sxl’s function in the *Drosophila* nervous system, where it regulates Pol III activity during late brain development through interaction with Polr3E. Based on its expression pattern, we hypothesize that Sxl acts as a selective regulator of Pol III activity in metabolically demanding neuronal populations, such as mushroom body neurons [38,39]. Future investigations using electrophysiology will be crucial to uncover the physiological role of both Polr3E and Sxl^RAC^.

### Sxl^RAC^ mediates neuronal homeostasis

The Sxl^RAC^ isoform, naturally expressed in male brains [4,13,14], carries a truncation in its N-terminus, which abolishes its splicing activity [40]. While we observe a mild survival deficit in *Sxl* knockdowns, overexpression of *Sxl*^*RAC*^ in adults results in a marked decline in survival (Fig 5) and significant transcriptional changes associated with metabolic and translational processes (S4 Fig). These data underscore the importance of tightly regulating *Sxl*^*RAC*^ expression to maintain neuronal homeostasis. Since Sxl amorphs are male viable and fertile, this role is likely not essential [16]. The contrasting effects of knockdown and overexpression (both at the phenotypic and transcriptomic levels) point to a dosage-sensitive role for Sxl in adult neurons, with context-dependent requirements. The life span alterations observed in overexpression models are likely explained as consequences of dysregulated tRNA synthesis and/or metabolic trade-offs [41,42].

Using small RNA-seq and qPCR, we found that neuronal *Sxl*^*RAC*^ overexpression leads to changes in Pol III-transcribed loci, with a pronounced bias toward upregulation of tRNAs, particularly lysine tRNAs (Fig 5 and S5), as well as changes in other classes of small RNAs (Figs 5 and S6). These observations align with recent work in mammalian neurons, which highlights cell-type-specific tRNA expression and links dynamic tRNA pools to the regulation of neuronal homeostasis [43]. Our findings suggest that Sxl^RAC^ isoform expression is tuned to cellular requirements, with direct consequences for translational capacity and metabolic output.

This link between Sxl-dependent tRNA regulation and translation is further supported by OPP labeling, which revealed that cells with reduced Sxl expression also exhibit reduced nascent protein synthesis, suggesting a local role for translation regulation (Fig 5). Interestingly, the enrichment of lysine tRNAs raises the possibility that Sxl-dependent tRNA pools influence codon-specific translation efficiency. Lysyl-tRNAs are relevant in this context because their availability can affect decoding competition at termination sites, potentially promoting stop-codon readthrough at select transcripts, as reported in *Drosophila* neurons [44,45]. Such effects could provide a mechanism for fine-tuning proteome composition in highly active neurons.

Taken together, these data support a model in which Sxl is recruited to chromatin by Polr3E to modulate Pol III activity in adult male neurons, thereby filtering tRNA expression and supporting translational capacity and perhaps metabolism in highly active cells. Building on emerging evidence that Pol III inhibition can influence aging and tissue homeostasis [24,23], our work positions Sxl as a novel mediator of age-sensitive translation and homeostatic pathways in the nervous system. Future studies will be required to determine whether Sxl-dependent tRNA regulation contributes to codon-specific translation or readthrough events, and how these effects impact neuronal function and longevity.

### Sxl’s ancestral role

Our findings also raise intriguing questions about the conserved roles of Sxl and its orthologues across species. Though sexual dimorphism in *Sxl* expression is restricted to the *Drosophilidae* family, it has orthologues in other insect species, implying it may have evolved from a broader, sex-independent role, as seen in the Sxl^RAC^ isoform [13,46,47]. The evolution of Sxl as a sex-specific gene (~10 MY) likely reflects a later adaptation to regulate sexual differentiation [13]. However, this sex-independent role in regulating metabolic processes and RNA Pol III activity may be conserved, raising the possibility that Sxl orthologues, including in vertebrates, act in analogous pathways relevant to neuronal homeostasis. For instance, the ELAV family of RNA-binding proteins, which share sequence homology with Sxl, also regulate RNA stability and translation in response to metabolic and stress signals [21,48–50], suggesting similar regulatory mechanisms may be at work.

In conclusion, our study reveals a novel, non-splicing role for Sxl by binding chromatin and regulating Pol III transcription, with important implications for neuronal function, metabolism, and aging. These findings provide new insights into the broader functional repertoire of Sxl and underscore the potential to explore its conserved roles across species, from insects to vertebrates. Further research into the mechanisms governing Sxl’s chromatin recruitment and its interaction with Polr3E will be crucial for understanding its full range, and whether it is restricted to the nervous system.

## Materials and methods

### *Drosophila* husbandry

Crosses were established and maintained under conditions optimized for each experiment. For larval experiments, *elav*^*C155*^*-GAL4* virgin females were crossed to Dam lines (see below) to drive expression in early-developing neurons. For adult-specific investigations, *w*^*-*^*; tub-GAL80*^*ts*^*; nSyb-GAL4* virgin females were crossed to RNAi lines and corresponding controls: *UAS-Sxl-RNAi-I* (VDRC #109221), *UAS-Sxl-RNAi-II* (BDSC #34393), *UAS-Polr3E-RNAi* (BDSC #55656), *y; RNAi-TK* (VDRC #60101) and *y;p(UAS-CaryP)attP40* (BDSC #36304). In adult experiments, crosses were maintained at 18 °C throughout development, with offspring shifted to 29 °C for a defined period to induce GAL4-mediated expression. The *Sxl-T2A-GAL4/FM7* line (kindly provided by Jan Medenbach) was used to visualize *Sxl* expression in the adult brain. For localization and colocalization experiments, flies were crossed to *10xUAS-IVS-mCD8::GFP* (BDSC #32185) and *y; p(UAS-mCherry)attP2* (BDSC #35787) males, respectively.

### *Drosophila* transgenics

When generating *UAS*-Dam-splice factor constructs, *Sxl, U1, tra, U2AF50* and *X16* coding sequences were amplified by PCR from cDNA, digested with NotI and XbaI and cloned using the Gibson assembly strategy (using NEBuilder HiFi DNA Assembly kit (NEB)) into *pUASTattB-LT3-Dam* [20]. For *Sxl*^*RNA*^, 3 overlapping segments of *Sxl* were amplified with mismatch primers (to mutate the RNA binding sites) and simultaneously Gibson cloned into *pUASTattB-LT3-Dam. Sxl*^*GS*^ was generated by gene synthesis (Integrated DNA Technologies (IDT)) and cloned into *pUASTattB-LT3-Dam*. *For generating UAS-Sxl*^*RAC*^, the coding sequence was PCR amplified from cDNA and Gibson cloned into *pUASTattB*. To generate transgenics, these constructs were injected into *attP2* flies (*y w P{y[+t7.7]=nos-phiC31\int.NLS}X #12;; P{y[+t7.7]=CaryP}attP2;* Department of Genetics Microinjection Service, Cambridge) for integration on the third chromosome. In functional studies, experimental flies were compared to landing-site controls *y; p(UAS-mCherry)attP2* (BDSC #35787) crossed with the same driver.

### Behavioral and phenotypic assays

Survival assays were conducted as previously described [51]. Animals were reared at standardized larval densities and, upon eclosion, were allowed to mate for 24 h before being separated by sex. For each experimental condition, groups of 10 flies of the relevant genotype were transferred to vials containing fresh food. Vials were replaced every two days, and mortality was recorded at each transfer. To circumvent developmental effects, flies were reared at 18 °C and shifted to 29 °C post-eclosion to induce GAL4-driven expression by inactivating GAL80^ts^. Each condition was assessed with a minimum of 10 biological replicates to generate survival curves, which were compared to appropriate controls. Statistical analyses were performed using GraphPad Prism (v5.0). Significance was determined using the Gehan-Breslow-Wilcoxon and Log-rank (Mantel Cox) tests.

Age-related changes in locomotor performance were assessed using the Reactive Iterative Negative Geotaxis (RING) assay. A custom-designed apparatus was employed to ensure consistent starting conditions, with all flies dropped from a uniform height of 15 cm at a controlled velocity. For each experimental condition, three biological replicates were assessed, each consisting of 10 flies placed in an empty plastic vial. Flies were allowed to acclimate for 5 min prior to testing. Each group underwent three technical replicate trials, with 2-min rest intervals between drops. Climbing behavior was recorded through video (4K, 30 frames per second) using a handheld camera (12 MP). Performance was quantified by calculating the percentage of flies reaching the halfway mark (4 cm) within 10 s, shown on figures as ‘Climbing Success’. Data were analyzed using two-way ANOVA, followed by appropriate post hoc tests.

### Targeted DamID

Dam fusion and Dam-only males were crossed to *elav*^*C155*^-GAL4 virgins for expression of Dam in neurons. Fifty larval central nervous systems were dissected and stored at −80 °C. Genomic DNA extraction, methylated DNA enrichment and library preparation were carried out as previously described [52]. Two biological replicates were generated for each experiment. For the experiment profiling Sxl binding in neurons with *Polr3E* RNAi (BDSC #55656), the larvae were raised at 18 °C to prevent premature lethality. Three replicates were used for each condition (for *wild type*, ×1 female only sample and ×2 mixed sex samples, and for *Polr3E* RNAi, ×3 female samples—the female only sample is comparable to the mixed sex samples—see correlation coefficients in S8 Table).

Libraries were sequenced with 50 bp single-end sequencing on Illumina HiSeq platform. >10 million reads were obtained for each library. Data were processed using a previously described Perl pipeline [53] (https://github.com/owenjm/damidseq_pipeline).

Peak calling was performed using a previously described Perl program (available at https://github.com/tonysouthall/Peak_calling_DamID) which allows for the identification of broadly bound regions that characterize DamID data (Estacio-Gomez *and colleagues*, 2020). Briefly, false discovery rate (FDR) was calculated for regions consisting of >1 GATC-bounded fragments for each replicate. Significant (FDR < 0.01%) regions present in all replicates are merged to form a final peak file.

### Analysis of DamID targets and transcriptional directionality

Differentially expressed genes from RNA-seq datasets (adjusted *p*-value < 0.05) were intersected with Sxl-F-Dam binding sites identified from DamID experiments. DamID peaks were filtered to retain only high-confidence binding events (peak-calling *p*-value < 0.01) prior to integration with transcriptomic data.

Since RNA-seq provides gene-level resolution whereas DamID can report multiple binding peaks within a single gene locus, duplicate FBgn entries to the same gene were collapsed to a single representative entry. The distribution of log_2_ fold changes among target genes was then compared across conditions to determine whether Sxl-F-Dam binding was associated with a consistent regulatory bias.

### RNA-seq and data analysis

For each experimental condition, three biological replicates were prepared, with total RNA extracted from the heads of 20 flies per replicate using TRIzol reagent (Thermo Fisher Scientific), according to the manufacturer’s instructions. RNA sequencing library preparation and sequencing were performed by Genewiz (Azenta Life Sciences), generating ~20 million paired-end reads per sample.

Raw RNA-seq data were processed using the Galaxy platform (https://usegalaxy.eu). Adapter sequences were trimmed from FASTQ files using Trimmomatic (v0.39) with the ILLUMINACLIP parameter, and read quality was assessed using FastQC (v0.74). Reads were aligned to the *Drosophila* reference genome (release r6.58) using HISAT2 (v2.2.1). Gene-level quantification was performed with featureCounts (v2.0.8), and differential gene expression was analyzed using DESeq2 (v2.11.40.8). Gene Ontology (GO) enrichment analysis was conducted using tools provided by the Gene Ontology Consortium and PANTHER classification system.

### RNA-seq correlation analysis

Differential gene expression analyses were performed using DESeq2 output. Genes lacking log_2_ fold change estimates or adjusted *p*-values (NA entries) were excluded from downstream analysis.

To assess transcriptional concordance between conditions, raw log_2_ fold change values for shared FBgn-annotated genes were extracted and compared between datasets. Spearman correlation analysis was calculated in R (v4.2.2) to quantify the linear relationship between raw transcriptional responses (available in Supporting information). Analyses were then restricted to genes exhibiting significant differential expression (adjusted *p*-value < 0.05) in at least one condition. Correlation analysis was then repeated on this subset to assess concordance among condition-responsive genes (as shown in Figs 4 and S5).

Correlation coefficients and associated significance values were computed in R, and data visualized as scatter plots of log_2_ fold changes between conditions using the ggplot2 package.

### Small RNAseq and data analysis

Small RNA-seq libraries were prepared using a protocol analogous to standard RNA-seq library construction, with enrichment for small RNA fragments at sequencing stage. Sequencing was performed by Genewiz (Azenta Life Sciences), generating approximately 20 million paired-end reads per sample.

For small RNA-seq, data were processed independently using the Galaxy platform. Adapter trimming was performed with Trim Galore! (v0.6.7), and read quality was assessed using Falco (v1.2.4). Only Read 1 (R1) was used, as small RNAs are typically captured in R1, and Read 2 (R2) was excluded due to persistent adapter contamination after trimming. Reads were mapped to the *Drosophila* reference genome (r6.58) using Bowtie2 (v2.5.3), and small RNA species, including tRNAs, were quantified using featureCounts (v2.0.8). Differential expression analysis was again performed using DESeq2 (v2.11.40.8), and data visualization, including volcano plots, was completed in R (v4.2.2) using the ggplot2 package.

### Quantification of RNA Polymerase III targets

Following quantitative RNA extraction, reverse transcription was performed using the iScript cDNA Synthesis Kit (Bio‐Rad) with random hexamers, in accordance with the manufacturer’s instructions. Quantitative real-time PCR (qRT-PCR) was conducted using iTaq Universal SYBR Green Supermix (Bio‐Rad). Expression of target genes was normalized to U3, a small nucleolar RNA transcribed by RNA polymerase II and compared to expression levels in the corresponding control samples. Primers targeting pre-tRNA species were based on previously validated sequences [24–26]; all primer sequences are listed below.

pre-tRNAHisGTG-1 F CGTGATCGTCTAGTGGTTAG,
pre-tRNAHisGTG-1 R CCCAACTCCGTGACAATG,
pre-tRNAIleTAT-1 F CGCACGGTACTTATAATCAG,
pre-tRNAIleTAT-1 R, CCAGGTGAGGCTCGAACTC,
pre-tRNALeuCAC-1 F GCGCCAGACTCAAGATTG,
pre-tRNALeuCAC-1 R TGTCAGAAGTGGGATTCG,
U3 forward CACACTAGCTGAAAGCCAAG, and
U3 reverse CGAAGCCCTGCGTCCCGAAC.

For each genotype, ΔCq values were used for statistical testing, and relative fold change was used for plots. Sample sizes were too small to assess normality reliably; therefore, a one-tailed Mann–Whitney test was applied to account for the non-parametric and directional nature of the data.

### Immunohistochemistry

Immunohistochemistry was performed on third instar larval and adult central nervous systems (CNS). Dissections were carried out in 1× PBS, followed by fixation in 4% formaldehyde (methanol-free; Polysciences) diluted in PBS for 20 min at room temperature. Tissues were washed three times in PBST (0.3% Triton X-100 in PBS) for 5 min each, then blocked in 10% normal goat serum (NGS) in PBST for 1 hour at room temperature. Samples were incubated with primary antibodies overnight at 4 °C. The following day, tissues were washed three times in PBST and incubated with secondary antibodies diluted in PBST for 2 hours at room temperature, followed by final washes. Samples were mounted in VECTASHIELD Antifade Mounting Medium (Vector Laboratories) on standard glass slides. Primary antibodies used were mouse anti-Repo (1:500; 8D12 Developmental Studies Hybridoma Bank, DSHB) and rat anti-Elav (1:500; 7E8A10 DSHB). Secondary antibodies were Alexa Fluor 545 and 633 (1:200; Thermo Fisher Scientific). Images were subsequently analyzed using a Zeiss LSM 510 confocal microscope.

### O-propargyl-puromycin (OPP) assay

Protein synthesis was assessed using the Click-iT Plus OPP Assay Kit (Thermo Fisher Scientific), following the manufacturer’s instructions. Dissections were performed in Schneider’s Insect Medium to preserve neural tissue integrity. Isolated brains were incubated with OPP for 1 hour at room temperature to label nascent peptides. Following incubation, samples were fixed in 4% paraformaldehyde (PFA) for 15 min, then permeabilised and washed in 0.5% Triton X-100 in PBS (PBST). Click chemistry-based detection was carried out using a reaction cocktail composed of 880 µL 1× reaction buffer, 20 µL copper protectant, 100 µL 1× additive, and 2.5 µL Alexa Fluor picolyl azide. After the labeling reaction, samples were washed in 0.5% PBST. Samples were mounted in Vectashield mounting medium and imaged using a Leica Stellaris 5 light sheet microscope (LS1). All imaging acquisition parameters are detailed in S43 Table.

### Colocalization analysis

Colocalization analysis was performed in ImageJ (v1.54p) using the *Colocalization Threshold* plugin. Raw images were exported in 16-bit format and analyzed without compression to preserve fluorescence intensity information. Multichannel images were split into individual channels, and any preprocessing steps were applied identically.

Background subtraction was performed using the rolling ball algorithm (radius = 150 pixels) to remove low-frequency background signal while preserving biological structures. Quantification was performed on both background-corrected and raw images using Pearson’s correlation coefficient (*R*) (S23 Table). Overall *R*(total) and *R*(coloc) values were calculated by averaging across six brains. No regions of interest (ROIs) were selected to avoid spatial bias, and Costes’ automatic thresholding was applied to exclude additional background signal. Scatter plots were generated in ImageJ to visualize signal overlap (S9 Fig), and a baseline control group (nuclear blue) was included as a reference (S23 Table).

### Fluorescence intensity quantification

Fluorescence intensity in OPP-stained brains was quantified from confocal z-stacks using ImageJ (v1.54p). For each sample, z-stacks were combined to generate a single representative image per region. The central brain region was delineated to define a ROI, from which ROI area and mean gray value (MGV) were extracted. To control for inter-experimental variability, MGVs were normalized using a session standardization approach. Specifically, the mean MGV of all samples acquired within each experimental session was calculated, and individual sample MGVs were expressed relative to this session mean. Both raw and normalized fluorescence intensity values are reported (see S24, S25, S40, and S41 Tables).

## Supporting information

S1 Table Sxl DamID peaks. (CSV)

S2 Table Sxl GSmut DamID peaks. (CSV)

S3 Table Sxl RNAmut DamID peaks. (CSV)

S4 Table Sxl Ndel DamID peaks. (CSV)

S5 Table Sxl Cdel DamID peaks. (CSV)

S6 Table Polr3E DamID peaks. (CSV)

S7 Table tRNA binding Polr3E vs. Sxl. (CSV)

S8 Table Sxl DamID wt vs. Polr3E RNAi. (CSV)

S9 Table Sxl RNAi I survival. (CSV)

S10 Table Sxl RNAi I negative geotaxis. (CSV)

S11 Table Polr3E RNAi survival. (CSV)

S12 Table Polr3E RNAi negative geotaxis. (CSV)

S13 Table Sxl RNAi I RNAseq *p* < 0.05. (CSV)

S14 Table Sxl RNAi I RNAseq GO terms. (CSV)

S15 Table Polr3E RNAi RNAseq *p* < 0.05. (CSV)

S16 Table Polr3E RNAi RNAseq GO terms. (CSV)

S17 Table Sxl RNAi I vs. Polr3E RNAi RNAseq correlation. (CSV)

S18 Table Sxl RNAi I vs. Polr3E RNAi Dif Exp genes. (CSV)

S19 Table Sxl RAC srvival. (CSV)

S20 Table Sxl RAC negative geotaxis. (CSV)

S21 Table Sxl RAC small RNAseq tRNAs. (CSV)

S22 Table Sxl RAC small RNAseq other. (CSV)

S23 Table OPP colocalization. (CSV)

S24 Table OPP intensity Sxl RNAi I 14d. (CSV)

S25 Table OPP intensity Sxl RAC 14d. (CSV)

S26 Table Sxl RNAi II survival. (CSV)

S27 Table Sxl RNAi II negative geotaxis. (CSV)

S28 Table Sxl DamID vs. Sxl RNAi I RNAseq. (CSV)

S29 Table Sxl DamID vs. Polr3E RNAi RNAseq. (CSV)

S30 Table Sxl RAC RNAseq *p* < 0.05. (CSV)

S31 Table Sxl RAC RNAseq GO terms. (CSV)

S32 Table Sxl RNAmut survival. (CSV)

S33 Table Sxl RNAmut negative geotaxis. (CSV)

S34 Table Sxl RNAmut RNAseq *p* < 0.05. (CSV)

S35 Table Sxl RNAmut RNAseq GO terms. (CSV)

S36 Table Sxl RAC vs. Sxl RNAi I RNAseq correlation. (CSV)

S37 Table Sxl RAC vs. Sxl RNAmut RNAseq correlation. (CSV)

S38 Table Sxl RNAi I tRNA qPCR. (CSV)

S39 Table Sxl RAC tRNA qPCR. (CSV)

S40 Table OPP intensity Sxl RNAi I 4d. (CSV)

S41 Table OPP intensity Sxl RAC 4d. (CSV)

S42 Table Sxl RNAmut male vs. females eclosion. (CSV)

S43 Table Imaging metadata. (CSV)

S1 Fig Sxl-F robustly binds chromatin across the genome. TaDa profiles for Sxl-F across chromosomes X, 2L/R, 3L/R and 4. The *y*-axis represents the log_2_ ratio of Dam-Sxl-F binding over that of Dam-alone.
(TIF)

S2 Fig Sxl is expressed in the neurons of the male brain. **a,** Immunolabeled sections of the larval male brain showing *Sxl* expression (mCD8–GFP, green), the glial marker *repo* (magenta), and neurons labeled with *Elav* (blue). **b,** Equivalent labeling in adult brain sections. White arrows indicate *Sxl*-positive cells that colocalise with *Elav* but not with *repo*, consistent with neuron-specific expression. Images were captured at 40x magnification; scale bar, 30 µm.
(TIF)

S3 Fig *Sxl* knockdown phenocopies *Polr3E* Loss in adult males. **a,** Survival curves of adult males following pan-neuronal knockdown of *Sxl* using an independent RNAi line (BDSC #38195) compared with RNAi controls (BDSC #36304) (*n* > 90 per group). Statistical significance was assessed using the Gehan-Breslow-Wilcoxon and Log-rank tests. **b,** Negative geotaxis assay showing no significant changes in climbing ability following *Sxl* knockdown with the same RNAi line. The *y*-axis represents the percentage of flies surpassing the 4-cm midpoint. **c,** Full-size volcano plot displaying differential gene expression following *Sxl* knockdown in adult male neurons (VDRC #109221) relative to controls (VDRC #60101). Significantly upregulated transcripts (*p* < 0.05) are shown in light blue, and significantly downregulated transcripts (*p* < 0.05) in dark blue. **d,** Pie chart illustrating that 65% of Dam-Sxl-F chromatin targets are significantly downregulated upon *Sxl* knockdown in adult male neurons. **e,** Equivalent analysis showing that 69% of Dam-Sxl-F targets are similarly downregulated following *Polr3E* knockdown. Data underlying this figure are available in S26, S27, S28, and S29 Tables.
(TIF)

S4 Fig Elevated *Sxl*^*RAC*^ expression upregulates genes associated with metabolism and translation. Gene ontology (GO) enrichment analysis of transcripts upregulated following increased *Sxl*^*RAC*^ expression in adult male neurons. The dot plot shows the top 10 GO terms ranked by fold enrichment (*x*-axis); dot size indicates the number of gene hits, and color reflects the false discovery rate (FDR). Data underlying this figure are available in S30 and S31 Tables.
(TIF)

S5 Fig *Sxl*^*RAC*^ phenotypes correlate with those of RNA-binding mutants. **a,** Spearman correlation analysis of significant transcriptional changes in *Sxl*^*RAC*^ and *Sxl*^*RNA*^-expressing neurons reveals a significant positive relationship (*ρ* = 0.502, *p* = 6.92e−164). **b,** Spearman correlation analysis of all transcriptional changes in *Sxl*^*RAC*^ and *Sxl*^*RNA*^-expressing neurons shows similar positive relationship (*ρ* = 0.245, *p* = 3.66e−170). **c,** In contrast, a significant negative correlation is observed between *Sxl*^*RAC*^ overexpression and *Sxl* knockdown significant profiles (*ρ* = –0.355, *p* = 8.54e−74). **d,** Correlation analysis of all transcriptional changes in *Sxl*^*RAC*^ overexpression and *Sxl* knockdown reveals similar negative correlation (*ρ* = –0.124, *p* = 2.65e−41). **e,** Table highlighting the primary tRNA species upregulated upon *Sxl*^*RAC*^ overexpression, together with corresponding FBgn identifiers. Data underlying this figure are available in S21, S34, S35, S36, and S37 Tables.
(TIF)

S6 Fig Manipulating *Sxl* levels in male brains has a mild impact on core pre-tRNA levels. **a–b,** Pre-tRNA levels trend lower in *Sxl* RNAi-expressing heads (VDRC #109221), with stronger effects observed at 14 days. **a′,** Effects are negligible at 4 days of age, showing relative expression of *pre-tRNA*^*His*^ measured by qPCR (*n* = 6, *p* = 0.197, one-tailed Mann–Whitney test). **a″,** Relative expression of *pre-tRNA*^*Ile*^ (*n* = 6, *p* = 0.469). **a‴,** Relative expression of *pre-tRNA*^*Leu*^ (*n* = 6, *p* = 0.35). **b′,** Effects are more pronounced at 14 days of age, with relative expression of *pre-tRNA*^*His*^ measured by qPCR (*n* = 4, *p* = 0.029, one-tailed Mann–Whitney test). **b″,** Relative expression of *pre-tRNA*^*Ile*^ (*n* = 4, *p* = 0.057). **b‴,** Relative expression of *pre-tRNA*^*Leu*^ (*n* = 4, *p* = 0.171). **c–d,** Overexpression of *Sxl*^*RAC*^ in male neurons mildly increases pre-tRNA abundance at both time points. **c′,** Relative expression of *pre-tRNA*^*His*^ measured by qPCR (*n* = 6, *p* = 0.066, one-tailed Mann–Whitney test). **c″,** Relative expression of *pre-tRNA*^*Ile*^ (*n* = 6, *p* = 0.021). **c‴,** Relative expression of *pre-tRNA*^*Leu*^ (*n* = 6, *p* = 0.242). **d′,** Effects are variably pronounced at 14 days of age, with relative expression of *pre-tRNA*^*His*^ measured by qPCR (*n* = 4, *p* = 0.5, one-tailed Mann–Whitney test). **d″,** Relative expression of *pre-tRNA*^*Ile*^ (*n* = 4, *p* = 0.029). **d‴,** Relative expression of *pre-tRNA*^*Leu*^ (*n* = 4, *p* = 0.5). Data underlying this figure are available in S38 and S39 Tables.
(TIF)

S7 Fig Sxl shows moderate colocalization with OPP-enriched brain regions. Immunolabeled sections of adult male brains showing protein synthesis (OPP, blue) and *Sxl* expression (mCherry, red). Pale dotted lines indicate regions of high OPP signal, whereas red dotted lines highlight areas of low OPP signal. Images were captured at 20× magnification and processed using enhanced contrast to remove background; scale bar, 50 µm. Data underlying this figure are available in S23 Table.
(TIF)

S8 Fig Neuronal regions with enriched *Sxl* expression exhibit elevated protein synthesis. **a,** Fixed adult brain section labeled with OPP to visualize nascent protein synthesis, alongside mCherry expression driven by *Sxl-T2A-GAL4* (*Sxl > mCherry*). Regions of elevated protein synthesis align with areas of enriched *Sxl* expression, notably within the mushroom bodies and medulla. Images were acquired at 20x magnification; scale bar, 50 μm. **b,** Immunolabeled z-stacks of adult central brains from 4-day-old male flies showing widespread OPP signal in controls (b′) and no change following neuronal *Sxl* knockdown (b″). **c,** Quantification of normalized OPP fluorescence intensity reveals no change following *Sxl* depletion (*p* = 0.998, unpaired *t* test, *n* = 8 brains). **d,** Immunolabeled z-stacks of adult central brains from 4-day-old male flies showing no change in OPP signal in neurons expressing *Sxl*^*RAC*^ (d″) compared with controls (d′). **e,** Quantification of normalized OPP fluorescence intensity reveals no change following *Sxl*^*RAC*^ expression (*p* = 0.453, unpaired *t* test, *n* = 9 brains). All secondary images were captured at 20× magnification; scale bar, 50 µm. Data underlying this figure are available in S40 and S41 Tables.
(TIF)

S9 Fig Colocalization threshold analysis of OPP with Sxl and Nuclear Blue. Scatter plots were generated in ImageJ to assess pixel intensity correlation between channels from the same set of images. In all plots, the *x*-axis represents OPP signal (Channel 1), while the *y*-axis represents either Sxl (Channel 2) or Nuclear Blue (Channel 3). **a,** Raw (non-preprocessed) colocalization analysis of OPP versus Sxl. **a′,** Background-subtracted (SB) colocalization analysis of OPP versus Sxl. **b,** Raw (non-preprocessed) colocalization analysis of OPP versus Nuclear Blue. **b′,** Background-subtracted (SB) colocalization analysis of OPP versus Nuclear Blue. All analyses were performed on identical image sets, enabling direct comparison between raw and background-subtracted conditions for each channel pair.
(TIF)

S10 Fig Quality control and differential expression analysis of RNA-seq data comparing mRNA levels in *Sxl* knockdowns and RNAi controls. **a,** Principal component analysis (PCA) of variance-stabilized gene expression data showing sample clustering by experimental condition (*Sxl* knockdown (VDRC #109221, blue) and RNAi controls (VDRC #60101, red)). Principal component 1 (PC1) is plotted on the *x*-axis and explains 43% of the total variance, while principal component 2 (PC2) is plotted on the *y*-axis and explains 35% of the total variance. **b,** Heatmap of sample-to-sample distances calculated from normalized feature counts across all samples. Color intensity represents the relative distance between samples, providing an assessment of sample similarity and clustering among experimental groups. **c,** Dispersion estimates for all detected genes. The *x*-axis represents mean normalized counts and the *y*-axis represents dispersion estimates. Black points indicate gene-wise dispersion estimates, the red line shows the fitted dispersion trend, and blue points represent the final dispersion estimates used for differential expression analysis. **d,** Histogram of *p*-values generated from differential expression testing. The *x*-axis shows *p*-values ranging from 0 to 1, and the *y*-axis indicates the frequency of genes within each *p*-value bin. The distribution provides an overview of the statistical significance profile across all tested genes. **e,** Uncropped MA plot comparing gene expression between conditions *Sxl* knockdown compared and RNAi controls. The *x*-axis represents mean normalized counts, and the *y*-axis represents log_2_ fold change. Each point corresponds to a gene, with blue-highlighted points indicating genes exhibiting statistically significant transcriptional changes between the two conditions.
(PDF)

S11 Fig Quality control and differential expression analysis of RNA-seq data comparing mRNA levels in *Polr3E* knockdowns and RNAi controls. **a,** Principal component analysis (PCA) of variance-stabilized gene expression data showing sample clustering by experimental condition (*Polr3E* knockdown (BDSC #55656, blue) and RNAi controls (BDSC #36304, red)). Principal component 1 (PC1) is plotted on the *x*-axis and explains 52% of the total variance, while principal component 2 (PC2) is plotted on the *y*-axis and explains 22% of the total variance. **b,** Heatmap of sample-to-sample distances calculated from normalized feature counts across all samples. Color intensity represents the relative distance between samples, providing an assessment of sample similarity and clustering among experimental groups. **c,** Dispersion estimates for all detected genes. The *x*-axis represents mean normalized counts and the *y*-axis represents dispersion estimates. Black points indicate gene-wise dispersion estimates, the red line shows the fitted dispersion trend, and blue points represent the final dispersion estimates used for differential expression analysis. **d,** Histogram of *p*-values generated from differential expression testing. The *x*-axis shows *p*-values ranging from 0 to 1, and the *y*-axis indicates the frequency of genes within each *p*-value bin. The distribution provides an overview of the statistical significance profile across all tested genes. **e,** Uncropped MA plot comparing gene expression between conditions *Polr3E* knockdown and RNAi controls. The *x*-axis represents mean normalized counts, and the *y*-axis represents log_2_ fold change. Each point corresponds to a gene, with blue-highlighted points indicating genes exhibiting statistically significant transcriptional changes between the two conditions.
(PDF)

S12 Fig Quality control and differential expression analysis of small RNA-seq data comparing tRNA levels in *Sxl*^*RAC*^ and *mCherry* controls. **a,** Principal component analysis (PCA) of variance-stabilized gene expression data showing sample clustering by experimental condition (*Sxl*^*RAC*^ (blue) and *mCherry* controls (BDSC #35787, red)). Principal component 1 (PC1) is plotted on the *x*-axis and explains 58% of the total variance, while principal component 2 (PC2) is plotted on the *y*-axis and explains 17% of the total variance. **b,** Heatmap of sample-to-sample distances calculated from normalized feature counts across all samples. Color intensity represents the relative distance between samples, providing an assessment of sample similarity and clustering among experimental groups. **c,** Dispersion estimates for all detected genes. The *x*-axis represents mean normalized counts and the *y*-axis represents dispersion estimates. Black points indicate gene-wise dispersion estimates, the red line shows the fitted dispersion trend, and blue points represent the final dispersion estimates used for differential expression analysis. **d,** Histogram of *p*-values generated from differential expression testing. The *x*-axis shows *p*-values ranging from 0 to 1, and the *y*-axis indicates the frequency of genes within each *p*-value bin. The distribution provides an overview of the statistical significance profile across all tested genes. **e,** Uncropped MA plot comparing gene expression between conditions *Sxl*^*RAC*^ and *mCherry* controls. The *x*-axis represents mean normalized counts, and the *y*-axis represents log_2_ fold change. Each point corresponds to a gene, with blue-highlighted points indicating genes exhibiting statistically significant transcriptional changes between the two conditions.
(PDF)

S13 Fig Quality control and differential expression analysis of small RNA-seq data comparing miRNA levels in *Sxl*^*RAC*^ and *mCherry* controls. **a,** Principal component analysis (PCA) of variance-stabilized gene expression data showing sample clustering by experimental condition (*Sxl*^*RAC*^ (blue) and *mCherry* controls (BDSC #35787, red)). Principal component 1 (PC1) is plotted on the *x*-axis and explains 71% of the total variance, while principal component 2 (PC2) is plotted on the *y*-axis and explains 14% of the total variance. **b,** Heatmap of sample-to-sample distances calculated from normalized feature counts across all samples. Color intensity represents the relative distance between samples, providing an assessment of sample similarity and clustering among experimental groups. **c,** Dispersion estimates for all detected genes. The *x*-axis represents mean normalized counts and the *y*-axis represents dispersion estimates. Black points indicate gene-wise dispersion estimates, the red line shows the fitted dispersion trend, and blue points represent the final dispersion estimates used for differential expression analysis. **d,** Histogram of *p*-values generated from differential expression testing. The *x*-axis shows *p*-values ranging from 0 to 1, and the *y*-axis indicates the frequency of genes within each *p*-value bin. The distribution provides an overview of the statistical significance profile across all tested genes. **e,** Uncropped MA plot comparing gene expression between conditions *Sxl*^*RAC*^ and *mCherry* controls. The *x*-axis represents mean normalized counts, and the *y*-axis represents log_2_ fold change. Each point corresponds to a gene, with blue-highlighted points indicating genes exhibiting statistically significant transcriptional changes between the two conditions.
(PDF)

S14 Fig Quality control and differential expression analysis of small RNA-seq data comparing rRNA levels in *Sxl*^*RAC*^ and *mCherry* controls. **a,** Principal component analysis (PCA) of variance-stabilized gene expression data showing sample clustering by experimental condition (*Sxl*^*RAC*^ (blue) and *mCherry* controls (BDSC #35787, red)). Principal component 1 (PC1) is plotted on the *x*-axis and explains 37% of the total variance, while principal component 2 (PC2) is plotted on the *y*-axis and explains 19% of the total variance. **b,** Heatmap of sample-to-sample distances calculated from normalized feature counts across all samples. Color intensity represents the relative distance between samples, providing an assessment of sample similarity and clustering among experimental groups. **c,** Dispersion estimates for all detected genes. The *x*-axis represents mean normalized counts and the *y*-axis represents dispersion estimates. Black points indicate gene-wise dispersion estimates, the red line shows the fitted dispersion trend, and blue points represent the final dispersion estimates used for differential expression analysis. **d,** Histogram of *p*-values generated from differential expression testing. The *x*-axis shows *p*-values ranging from 0 to 1, and the *y*-axis indicates the frequency of genes within each *p*-value bin. The distribution provides an overview of the statistical significance profile across all tested genes. **e,** Uncropped MA plot comparing gene expression between conditions *Sxl*^*RAC*^ and *mCherry* controls. The *x*-axis represents mean normalized counts, and the *y*-axis represents log_2_ fold change. Each point corresponds to a gene, with blue-highlighted points indicating genes exhibiting statistically significant transcriptional changes between the two conditions.
(PDF)

S15 Fig Quality control and differential expression analysis of small RNA-seq data comparing ncRNA levels in *Sxl*^*RAC*^ and *mCherry* controls. **a,** Principal component analysis (PCA) of variance-stabilized gene expression data showing sample clustering by experimental condition (*Sxl*^*RAC*^ (blue) and *mCherry* controls (BDSC #35787, red)). Principal component 1 (PC1) is plotted on the *x*-axis and explains 57% of the total variance, while principal component 2 (PC2) is plotted on the *y*-axis and explains 18% of the total variance. **b,** Heatmap of sample-to-sample distances calculated from normalized feature counts across all samples. Color intensity represents the relative distance between samples, providing an assessment of sample similarity and clustering among experimental groups. **c,** Dispersion estimates for all detected genes. The *x*-axis represents mean normalized counts and the *y*-axis represents dispersion estimates. Black points indicate gene-wise dispersion estimates, the red line shows the fitted dispersion trend, and blue points represent the final dispersion estimates used for differential expression analysis. **d,** Histogram of *p*-values generated from differential expression testing. The *x*-axis shows *p*-values ranging from 0 to 1, and the *y*-axis indicates the frequency of genes within each *p*-value bin. The distribution provides an overview of the statistical significance profile across all tested genes. **e,** Uncropped MA plot comparing gene expression between conditions *Sxl*^*RAC*^ and *mCherry* controls. The *x*-axis represents mean normalized counts, and the *y*-axis represents log_2_ fold change. Each point corresponds to a gene, with blue-highlighted points indicating genes exhibiting statistically significant transcriptional changes between the two conditions.
(PDF)

S16 Fig Quality control and differential expression analysis of small RNA-seq data comparing snRNA levels in *Sxl*^*RAC*^ and *mCherry* controls. **a,** Principal component analysis (PCA) of variance-stabilized gene expression data showing sample clustering by experimental condition (*Sxl*^*RAC*^ (blue) and *mCherry* controls (BDSC #35787, red)). Principal component 1 (PC1) is plotted on the *x*-axis and explains 42% of the total variance, while principal component 2 (PC2) is plotted on the *y*-axis and explains 22% of the total variance. **b,** Heatmap of sample-to-sample distances calculated from normalized feature counts across all samples. Color intensity represents the relative distance between samples, providing an assessment of sample similarity and clustering among experimental groups. **c,** Dispersion estimates for all detected genes. The *x*-axis represents mean normalized counts and the *y*-axis represents dispersion estimates. Black points indicate gene-wise dispersion estimates, the red line shows the fitted dispersion trend, and blue points represent the final dispersion estimates used for differential expression analysis. **d,** Histogram of *p*-values generated from differential expression testing. The *x*-axis shows *p*-values ranging from 0 to 1, and the *y*-axis indicates the frequency of genes within each *p*-value bin. The distribution provides an overview of the statistical significance profile across all tested genes. **e,** Uncropped MA plot comparing gene expression between conditions *Sxl*^*RAC*^ and *mCherry* controls. The *x*-axis represents mean normalized counts, and the *y*-axis represents log_2_ fold change. Each point corresponds to a gene, with blue-highlighted points indicating genes exhibiting statistically significant transcriptional changes between the two conditions.
(PDF)

S17 Fig Quality control and differential expression analysis of small RNA-seq data comparing snoRNA levels in *Sxl*^*RAC*^ and *mCherry* controls. **a,** Principal component analysis (PCA) of variance-stabilized gene expression data showing sample clustering by experimental condition (*Sxl*^*RAC*^ (blue) and *mCherry* controls (BDSC #35787, red)). Principal component 1 (PC1) is plotted on the *x*-axis and explains 40% of the total variance, while principal component 2 (PC2) is plotted on the *y*-axis and explains 22% of the total variance. **b,** Heatmap of sample-to-sample distances calculated from normalized feature counts across all samples. Color intensity represents the relative distance between samples, providing an assessment of sample similarity and clustering among experimental groups. **c,** Dispersion estimates for all detected genes. The *x*-axis represents mean normalized counts and the *y*-axis represents dispersion estimates. Black points indicate gene-wise dispersion estimates, the red line shows the fitted dispersion trend, and blue points represent the final dispersion estimates used for differential expression analysis. **d,** Histogram of *p*-values generated from differential expression testing. The *x*-axis shows *p*-values ranging from 0 to 1, and the *y*-axis indicates the frequency of genes within each *p*-value bin. The distribution provides an overview of the statistical significance profile across all tested genes. **e,** Uncropped MA plot comparing gene expression between conditions *Sxl*^*RAC*^ and *mCherry* controls. The *x*-axis represents mean normalized counts, and the *y*-axis represents log_2_ fold change. Each point corresponds to a gene, with blue-highlighted points indicating genes exhibiting statistically significant transcriptional changes between the two conditions.
(PDF)

S18 Fig Quality control and differential expression analysis of RNA-seq data comparing mRNA levels in *Sxl*^*RAC*^ and *mCherry* controls. **a,** Principal component analysis (PCA) of variance-stabilized gene expression data showing sample clustering by experimental condition (*Sxl*^*RAC*^ (blue) and *mCherry* controls (BDSC #35787, red)). Principal component 1 (PC1) is plotted on the *x*-axis and explains 57% of the total variance, while principal component 2 (PC2) is plotted on the *y*-axis and explains 34% of the total variance. **b,** Heatmap of sample-to-sample distances calculated from normalized feature counts across all samples. Color intensity represents the relative distance between samples, providing an assessment of sample similarity and clustering among experimental groups. **c,** Dispersion estimates for all detected genes. The *x*-axis represents mean normalized counts and the *y*-axis represents dispersion estimates. Black points indicate gene-wise dispersion estimates, the red line shows the fitted dispersion trend, and blue points represent the final dispersion estimates used for differential expression analysis. **d,** Histogram of *p*-values generated from differential expression testing. The *x*-axis shows *p*-values ranging from 0 to 1, and the *y*-axis indicates the frequency of genes within each *p*-value bin. The distribution provides an overview of the statistical significance profile across all tested genes. **e,** Uncropped MA plot comparing gene expression between conditions *Sxl*^*RAC*^ and *mCherry* controls. The *x*-axis represents mean normalized counts, and the *y*-axis represents log_2_ fold change. Each point corresponds to a gene, with blue-highlighted points indicating genes exhibiting statistically significant transcriptional changes between the two conditions.
(PDF)

S19 Fig Quality control and differential expression analysis of RNA-seq data comparing mRNA levels in *Sxl*^*RNA*^ and *mCherry* controls. **a,** Principal component analysis (PCA) of variance-stabilized gene expression data showing sample clustering by experimental condition (*Sxl*^*RNA*^ (blue) and *mCherry* controls (BDSC #35787, red)). Principal component 1 (PC1) is plotted on the *x*-axis and explains 51% of the total variance, while principal component 2 (PC2) is plotted on the *y*-axis and explains 25% of the total variance. **b,** Heatmap of sample-to-sample distances calculated from normalized feature counts across all samples. Color intensity represents the relative distance between samples, providing an assessment of sample similarity and clustering among experimental groups. **c,** Dispersion estimates for all detected genes. The *x*-axis represents mean normalized counts and the *y*-axis represents dispersion estimates. Black points indicate gene-wise dispersion estimates, the red line shows the fitted dispersion trend, and blue points represent the final dispersion estimates used for differential expression analysis. **d,** Histogram of *p*-values generated from differential expression testing. The *x*-axis shows *p*-values ranging from 0 to 1, and the *y*-axis indicates the frequency of genes within each *p*-value bin. The distribution provides an overview of the statistical significance profile across all tested genes. **e,** Uncropped MA plot comparing gene expression between conditions *Sxl*^*RNA*^ and *mCherry* controls. The *x*-axis represents mean normalized counts, and the *y*-axis represents log_2_ fold change. Each point corresponds to a gene, with blue-highlighted points indicating genes exhibiting statistically significant transcriptional changes between the two conditions.
(PDF)

## Abbreviations

CNS: central nervous systems
Dam: DNA adenine methyltransferase
FDR: false discovery rate
IDT: Integrated DNA Technologies
MGV: mean gray value
NGS: normal goat serum
OPP: O-propargyl-puromycin
PFA: paraformaldehyde
qRT-PCR: quantitative real-time PCR
RBDs: RNA-Binding Domains
RING: Reactive Iterative Negative Geotaxis
ROIs: regions of interest
RNP: ribonucleoprotein
Sxl: Sex-lethal
TaDa: Targeted DamID
TIF: Tra-insufficient Feminization
TSS: transcriptional start sites
