# Supplementary figures and images for: Neuronal protein Sex-lethal modulates tRNA synthesis via the polymerase III subunit Polr3E in male Drosophila neurons
Source: PLoS Biol. 2026 Jul 17;24(7):e3003863. doi: 10.1371/journal.pbio.3003863 (PMC13432123; doi:10.1371/journal.pbio.3003863)

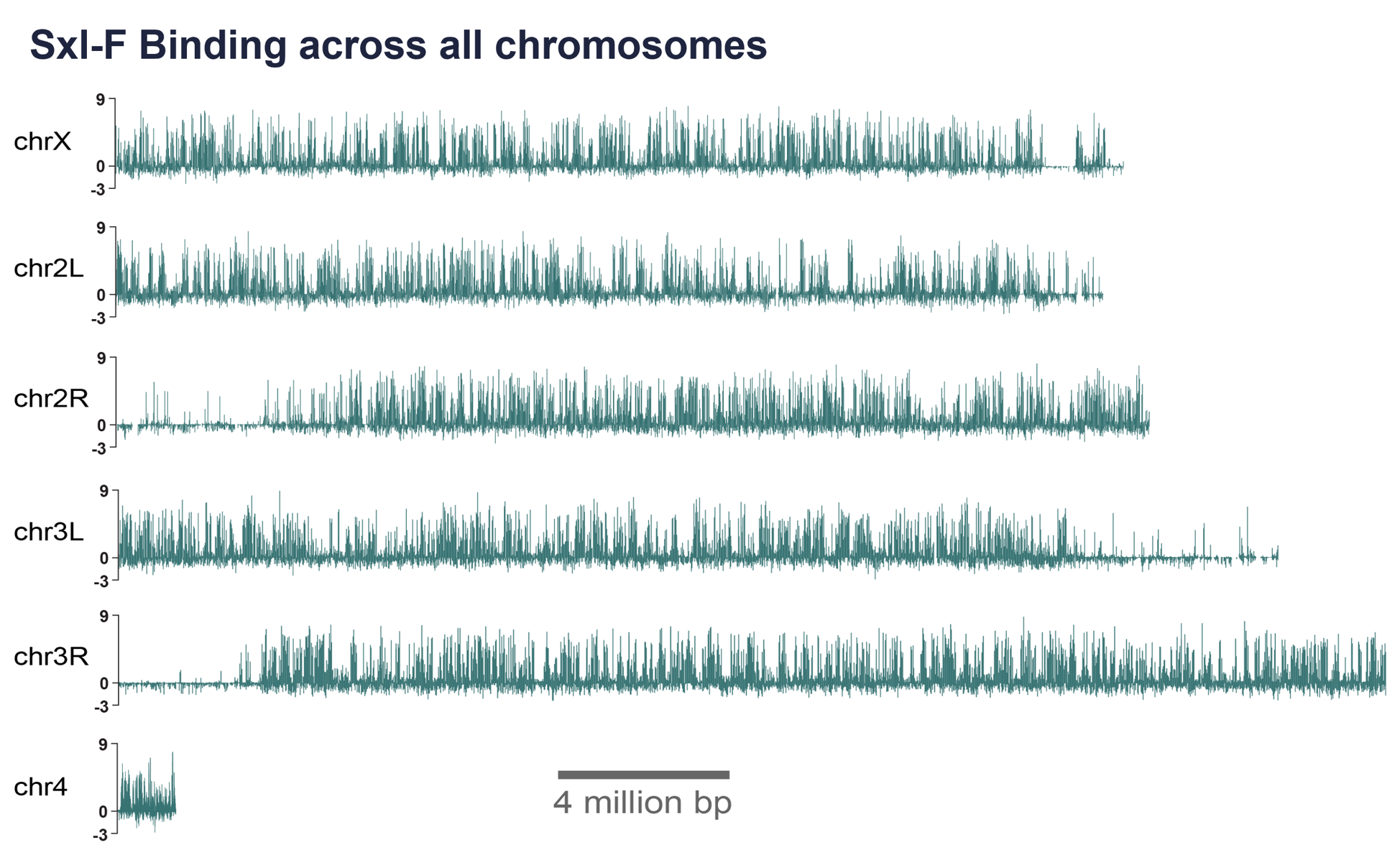

Supplement: S1 Fig — TaDa profiles for Sxl-F across chromosomes X, 2L/R, 3L/R and 4. The y-axis represents the log2 ratio of Dam-Sxl-F binding over that of Dam-alone. (TIF) [file pbio.3003863.s044.tif]

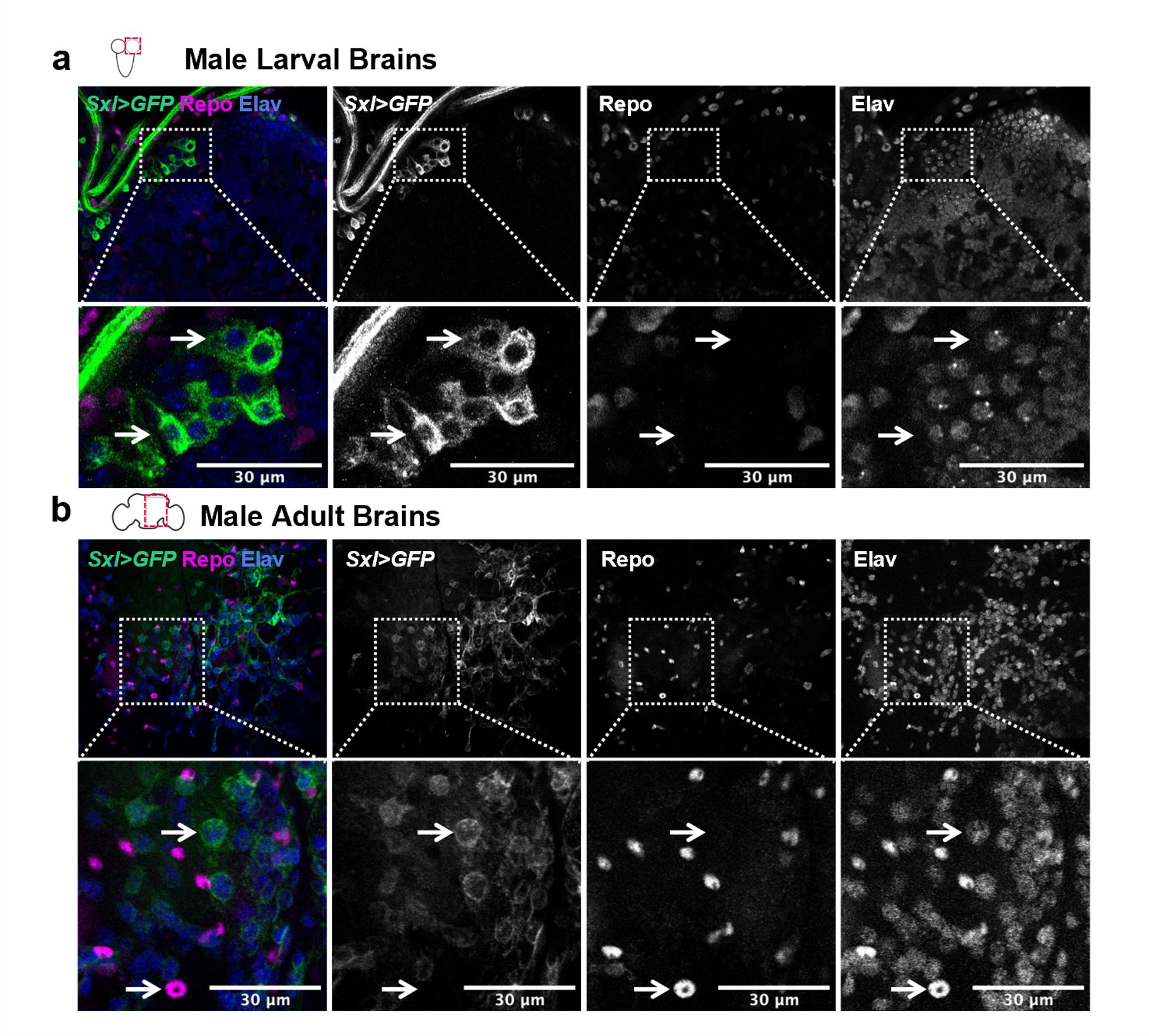

Supplement: S2 Fig — a, Immunolabeled sections of the larval male brain showing Sxl expression (mCD8–GFP, green), the glial marker repo (magenta), and neurons labeled with Elav (blue). b, Equivalent labeling in adult brain sections. White arrows indicate Sxl-positive cells that colocalise with Elav but not with repo, consistent with neuron-specific expression. Images were captured at 40x magnification; scale bar, 30 µm. (TIF) [file pbio.3003863.s045.tif]

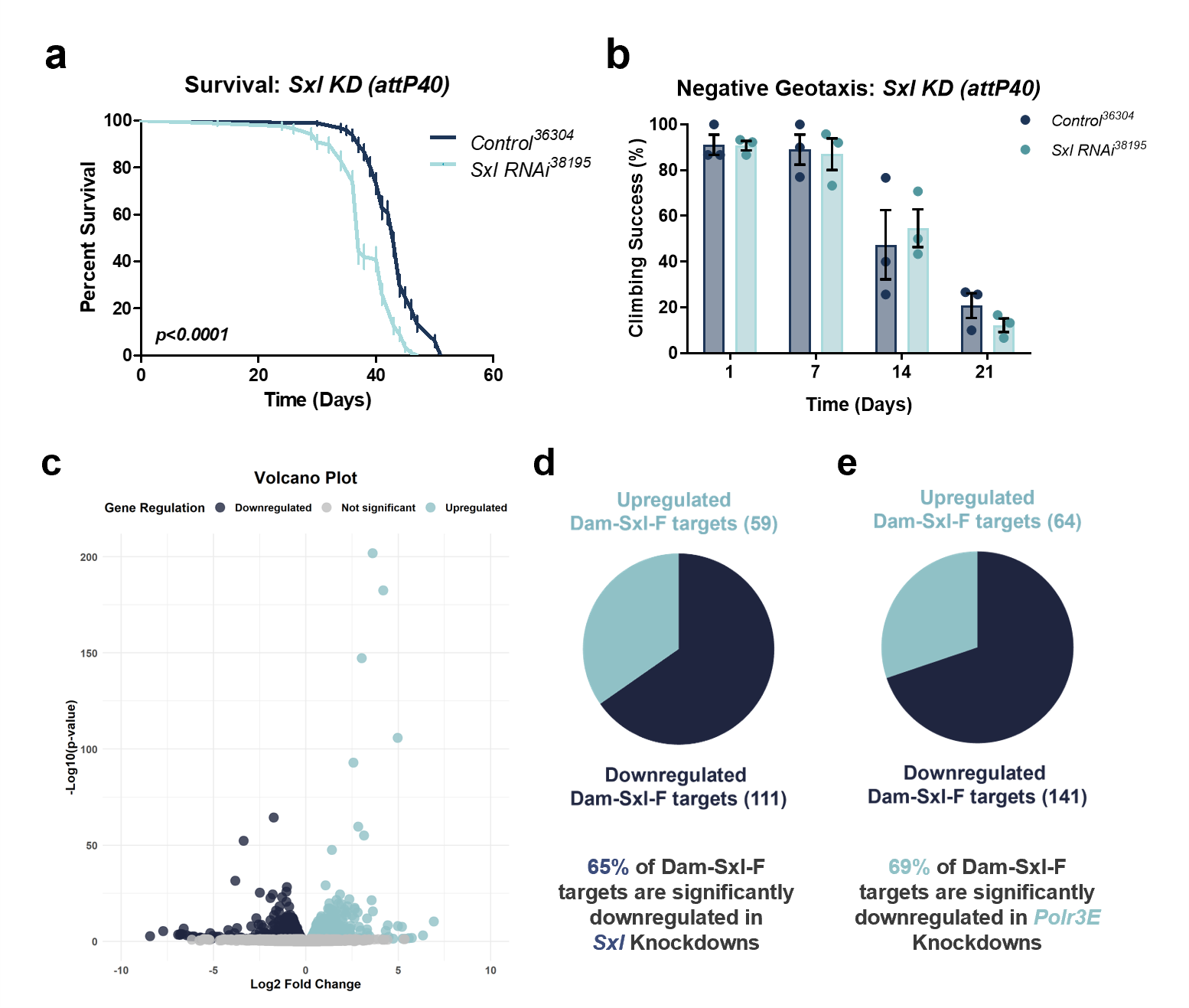

Supplement: S3 Fig — a, Survival curves of adult males following pan-neuronal knockdown of Sxl using an independent RNAi line (BDSC #38195) compared with RNAi controls (BDSC #36304) (n > 90 per group). Statistical significance was assessed using the Gehan-Breslow-Wilcoxon and Log-rank tests. b, Negative geotaxis assay showing no significant changes in climbing ability following Sxl knockdown with the same RNAi line. The y-axis represents the percentage of flies surpassing the 4-cm midpoint. c, Full-size volcano plot displaying differential gene expression following Sxl knockdown in adult male neurons (VDRC #109221) relative to controls (VDRC #60101). Significantly upregulated transcripts (p < 0.05) are shown in light blue, and significantly downregulated transcripts (p < 0.05) in dark blue. d, Pie chart illustrating that 65% of Dam-Sxl-F chromatin targets are significantly downregulated upon Sxl knockdown in adult male neurons. e, Equivalent analysis showing that 69% of Dam-Sxl-F targets are similarly downregulated following Polr3E knockdown. Data underlying this figure are available in S26, S27, S28, and S29 Tables. (TIF) [file pbio.3003863.s046.tif]

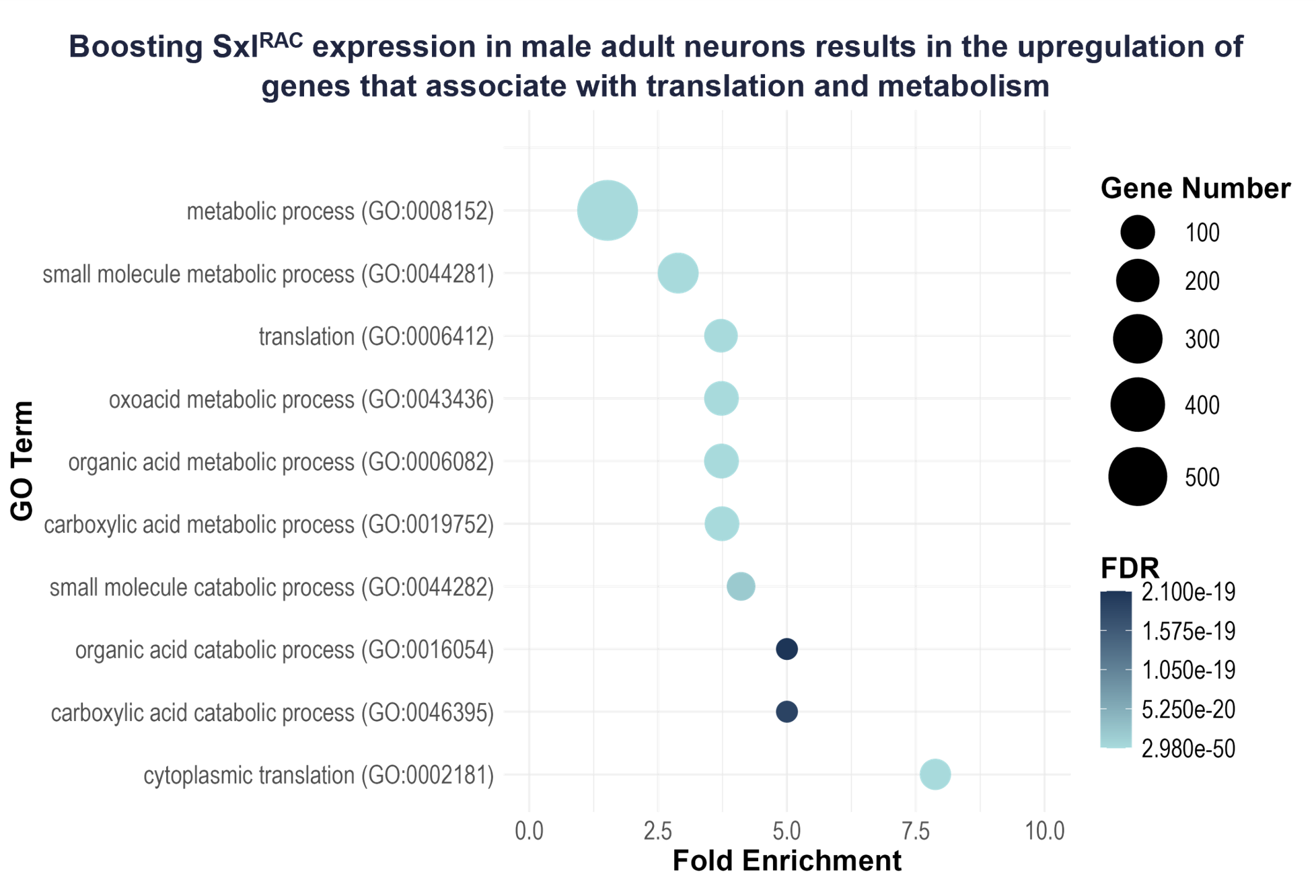

Supplement: S4 Fig — Gene ontology (GO) enrichment analysis of transcripts upregulated following increased SxlRAC expression in adult male neurons. The dot plot shows the top 10 GO terms ranked by fold enrichment (x-axis); dot size indicates the number of gene hits, and color reflects the false discovery rate (FDR). Data underlying this figure are available in S30 and S31 Tables. (TIF) [file pbio.3003863.s047.tif]

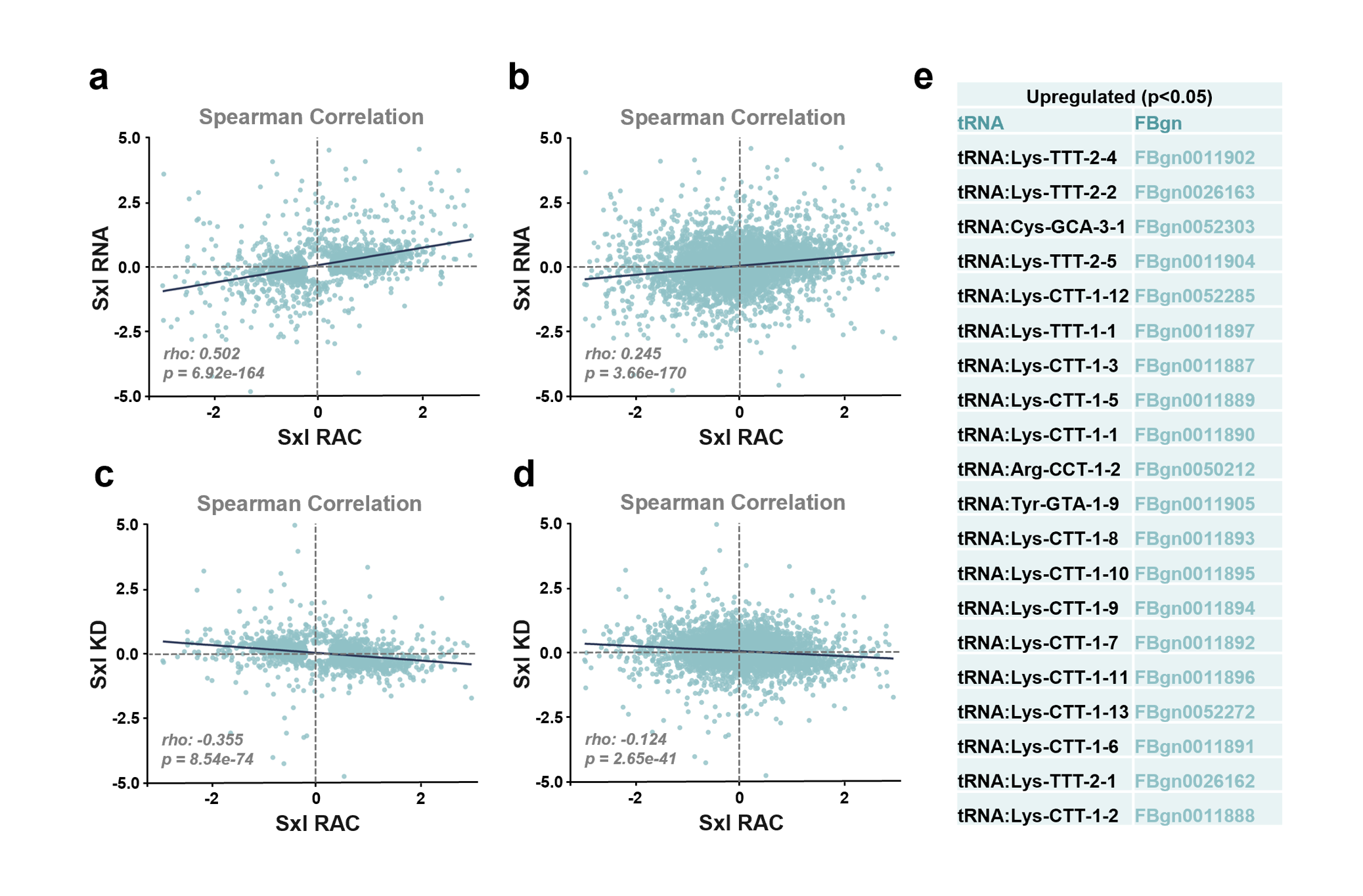

Supplement: S5 Fig — a, Spearman correlation analysis of significant transcriptional changes in SxlRAC and SxlRNA-expressing neurons reveals a significant positive relationship (ρ = 0.502, p = 6.92e−164). b, Spearman correlation analysis of all transcriptional changes in SxlRAC and SxlRNA-expressing neurons shows similar positive relationship (ρ = 0.245, p = 3.66e−170). c, In contrast, a significant negative correlation is observed between SxlRAC overexpression and Sxl knockdown significant profiles (ρ = –0.355, p = 8.54e−74). d, Correlation analysis of all transcriptional changes in SxlRAC overexpression and Sxl knockdown reveals similar negative correlation (ρ = –0.124, p = 2.65e−41). e, Table highlighting the primary tRNA species upregulated upon SxlRAC overexpression, together with corresponding FBgn identifiers. Data underlying this figure are available in S21, S34, S35, S36, and S37 Tables. (TIF) [file pbio.3003863.s048.tif]

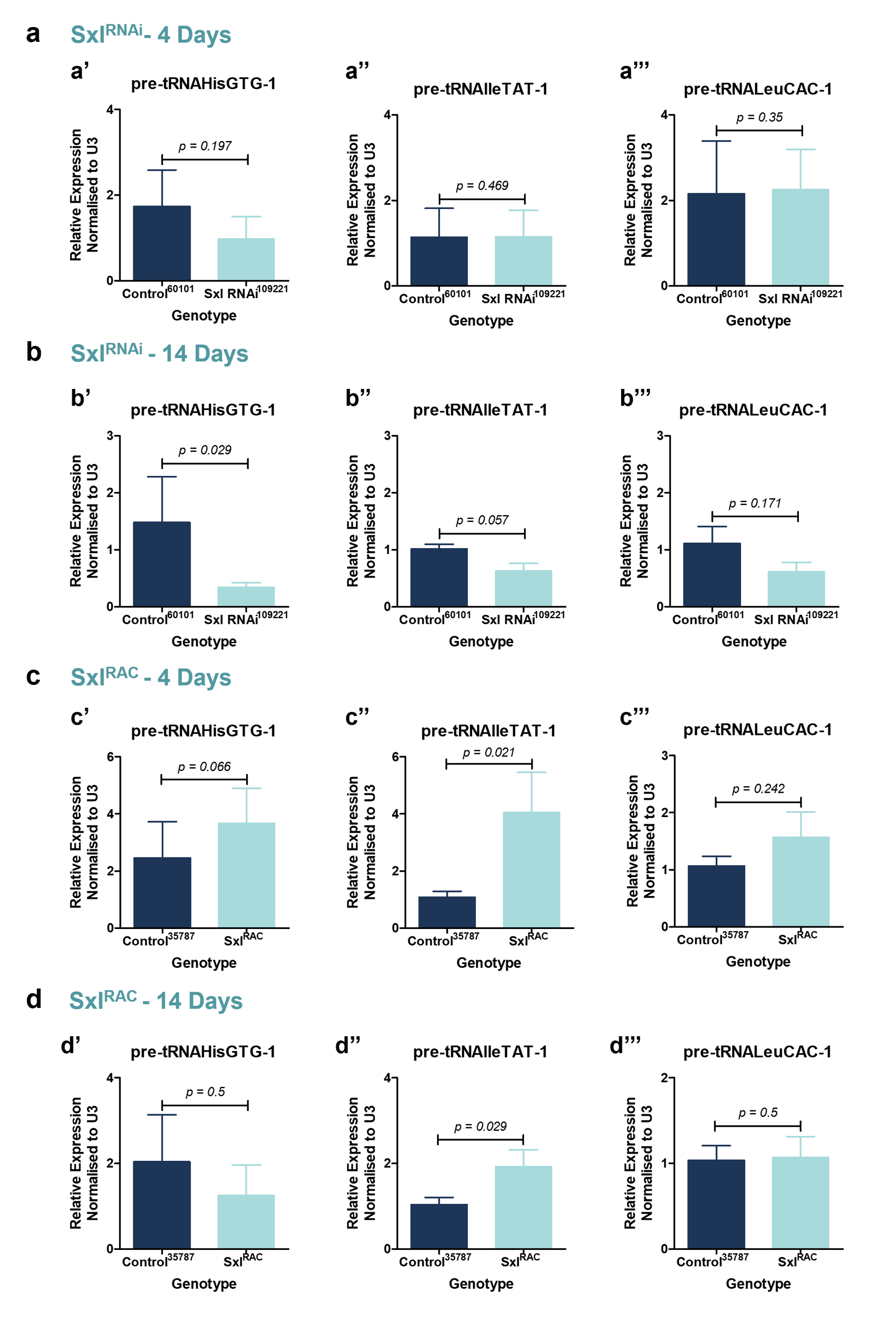

Supplement: S6 Fig — a–b, Pre-tRNA levels trend lower in Sxl RNAi-expressing heads (VDRC #109221), with stronger effects observed at 14 days. a′, Effects are negligible at 4 days of age, showing relative expression of pre-tRNAHis measured by qPCR (n = 6, p = 0.197, one-tailed Mann–Whitney test). a″, Relative expression of pre-tRNAIle (n = 6, p = 0.469). a‴, Relative expression of pre-tRNALeu (n = 6, p = 0.35). b′, Effects are more pronounced at 14 days of age, with relative expression of pre-tRNAHis measured by qPCR (n = 4, p = 0.029, one-tailed Mann–Whitney test). b″, Relative expression of pre-tRNAIle (n = 4, p = 0.057). b‴, Relative expression of pre-tRNALeu (n = 4, p = 0.171). c–d, Overexpression of SxlRAC in male neurons mildly increases pre-tRNA abundance at both time points. c′, Relative expression of pre-tRNAHis measured by qPCR (n = 6, p = 0.066, one-tailed Mann–Whitney test). c″, Relative expression of pre-tRNAIle (n = 6, p = 0.021). c‴, Relative expression of pre-tRNALeu (n = 6, p = 0.242). d′, Effects are variably pronounced at 14 days of age, with relative expression of pre-tRNAHis measured by qPCR (n = 4, p = 0.5, one-tailed Mann–Whitney test). d″, Relative expression of pre-tRNAIle (n = 4, p = 0.029). d‴, Relative expression of pre-tRNALeu (n = 4, p = 0.5). Data underlying this figure are available in S38 and S39 Tables. (TIF) [file pbio.3003863.s049.tif]

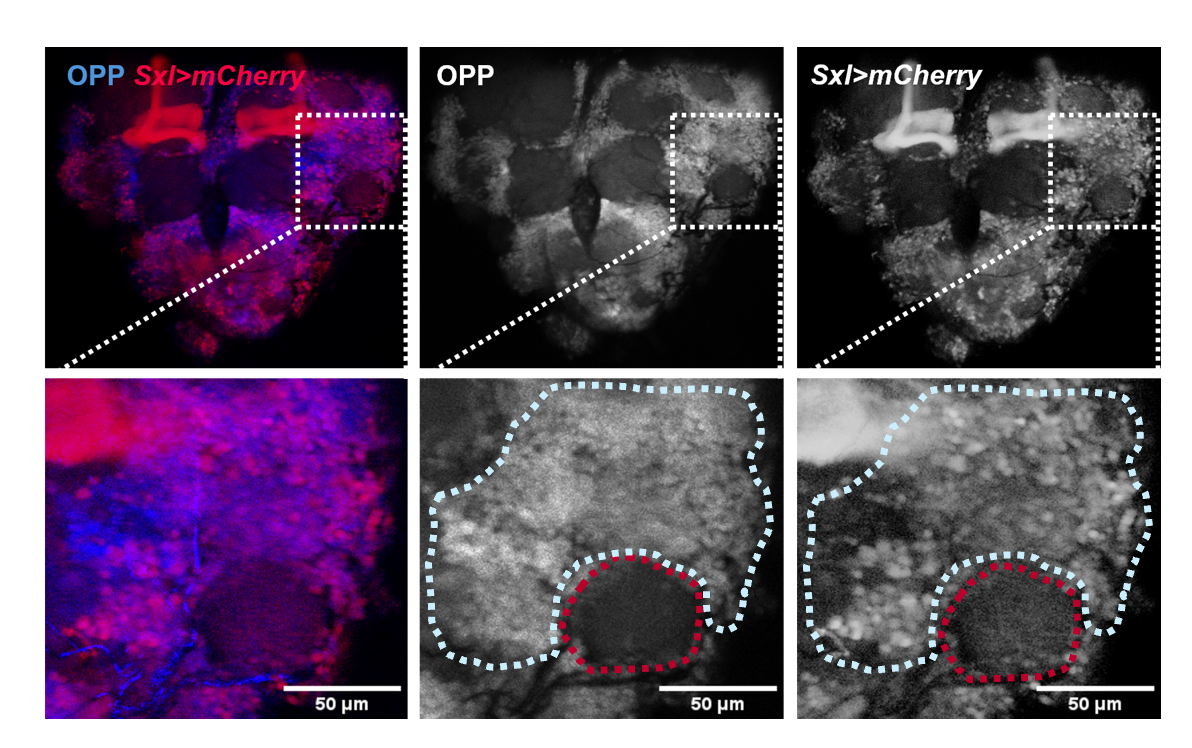

Supplement: S7 Fig — Immunolabeled sections of adult male brains showing protein synthesis (OPP, blue) and Sxl expression (mCherry, red). Pale dotted lines indicate regions of high OPP signal, whereas red dotted lines highlight areas of low OPP signal. Images were captured at 20× magnification and processed using enhanced contrast to remove background; scale bar, 50 µm. Data underlying this figure are available in S23 Table. (TIF) [file pbio.3003863.s050.tif]

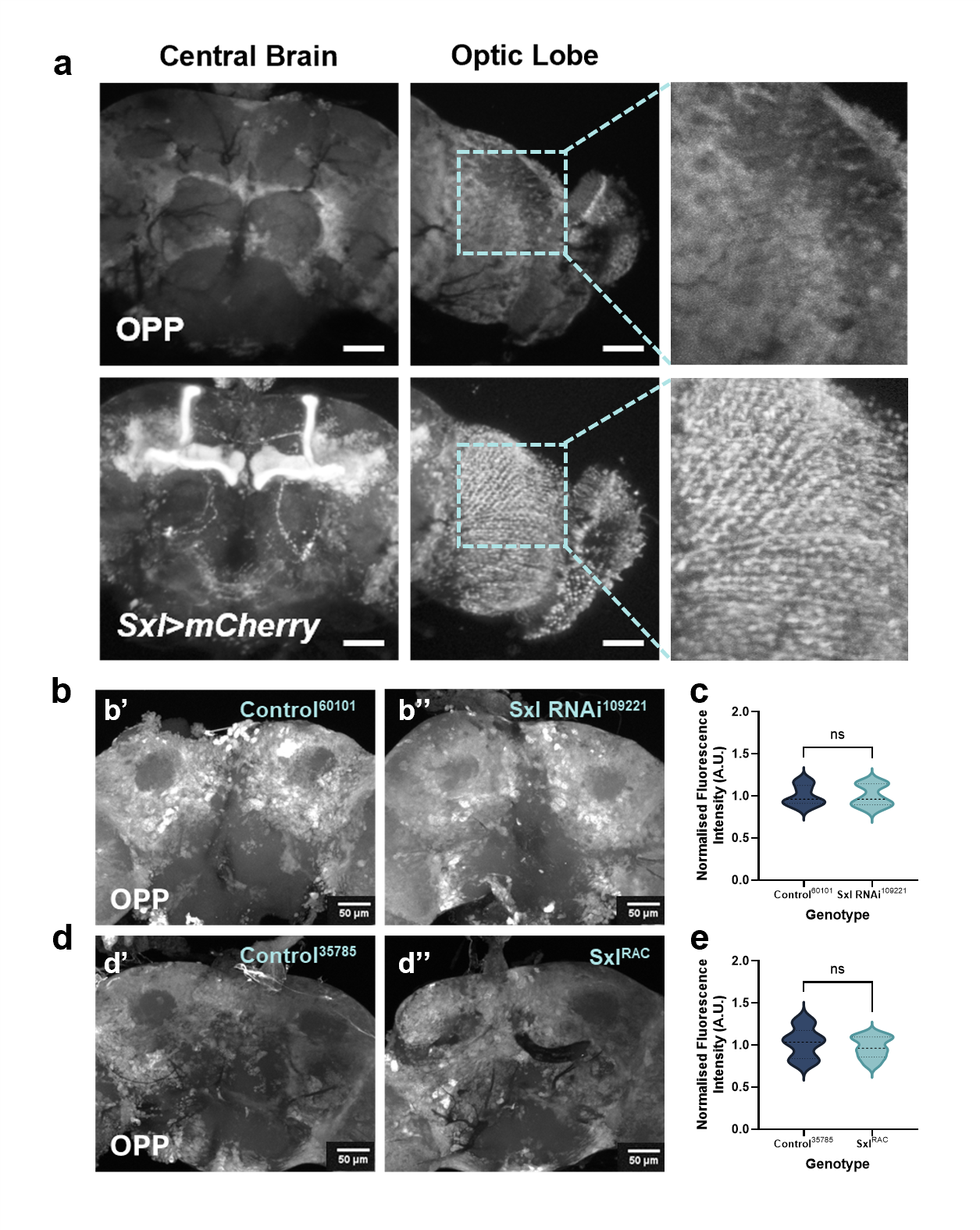

Supplement: S8 Fig — a, Fixed adult brain section labeled with OPP to visualize nascent protein synthesis, alongside mCherry expression driven by Sxl-T2A-GAL4 (Sxl > mCherry). Regions of elevated protein synthesis align with areas of enriched Sxl expression, notably within the mushroom bodies and medulla. Images were acquired at 20x magnification; scale bar, 50 μm. b, Immunolabeled z-stacks of adult central brains from 4-day-old male flies showing widespread OPP signal in controls (b′) and no change following neuronal Sxl knockdown (b″). c, Quantification of normalized OPP fluorescence intensity reveals no change following Sxl depletion (p = 0.998, unpaired t test, n = 8 brains). d, Immunolabeled z-stacks of adult central brains from 4-day-old male flies showing no change in OPP signal in neurons expressing SxlRAC (d″) compared with controls (d′). e, Quantification of normalized OPP fluorescence intensity reveals no change following SxlRAC expression (p = 0.453, unpaired t test, n = 9 brains). All secondary images were captured at 20× magnification; scale bar, 50 µm. Data underlying this figure are available in S40 and S41 Tables. (TIF) [file pbio.3003863.s051.tif]

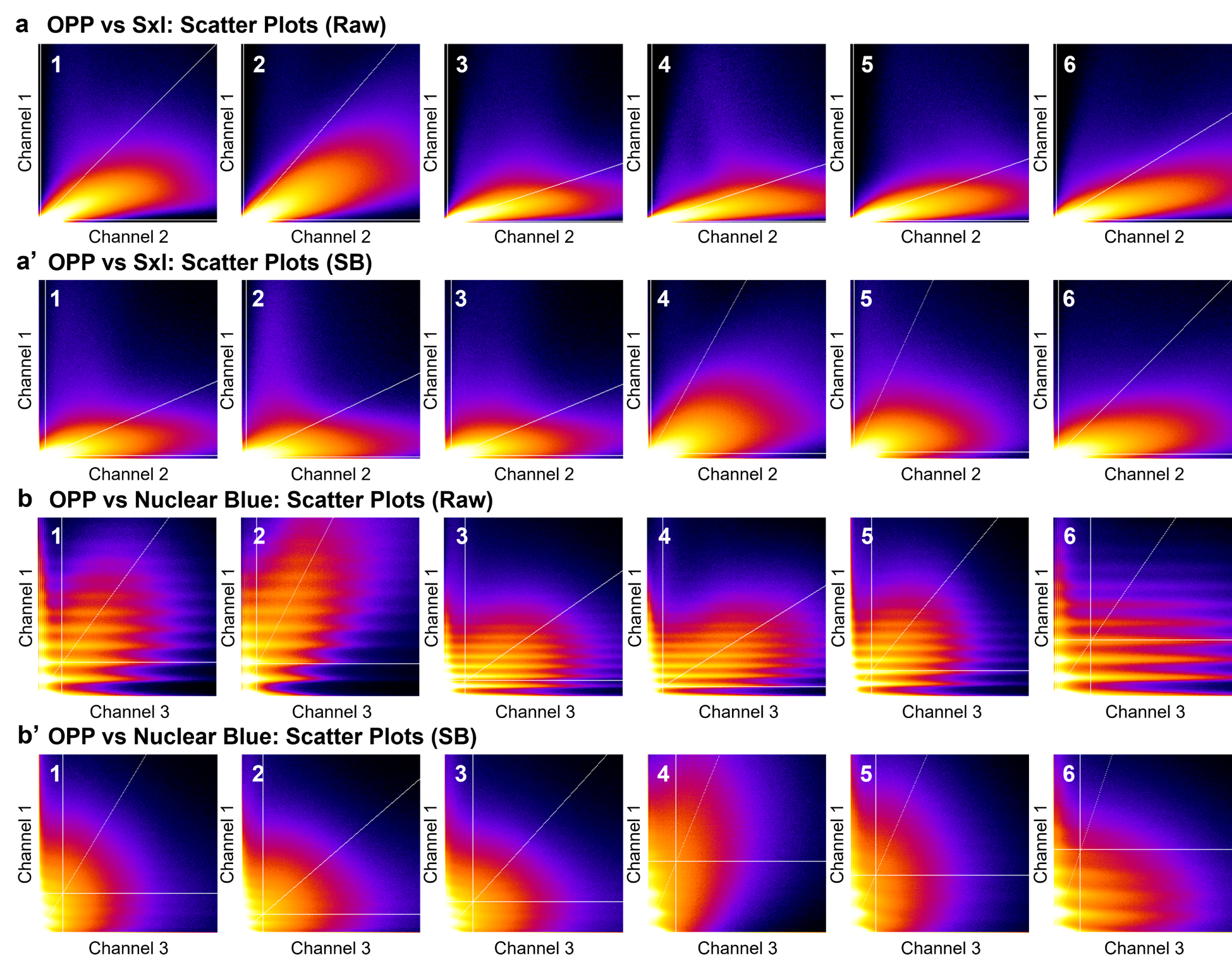

Supplement: S9 Fig — Scatter plots were generated in ImageJ to assess pixel intensity correlation between channels from the same set of images. In all plots, the x-axis represents OPP signal (Channel 1), while the y-axis represents either Sxl (Channel 2) or Nuclear Blue (Channel 3). a, Raw (non-preprocessed) colocalization analysis of OPP versus Sxl. a′, Background-subtracted (SB) colocalization analysis of OPP versus Sxl. b, Raw (non-preprocessed) colocalization analysis of OPP versus Nuclear Blue. b′, Background-subtracted (SB) colocalization analysis of OPP versus Nuclear Blue. All analyses were performed on identical image sets, enabling direct comparison between raw and background-subtracted conditions for each channel pair. (TIF) [file pbio.3003863.s052.tif]
