# Supplementary material for: Neuronal protein Sex-lethal modulates tRNA synthesis via the polymerase III subunit Polr3E in male Drosophila neurons
Source: PLoS Biol. 2026 Jul 17;24(7):e3003863. doi: 10.1371/journal.pbio.3003863 (PMC13432123; doi:10.1371/journal.pbio.3003863)
Supplement: S10 Fig — a, Principal component analysis (PCA) of variance-stabilized gene expression data showing sample clustering by experimental condition (Sxl knockdown (VDRC #109221, blue) and RNAi controls (VDRC #60101, red)). Principal component 1 (PC1) is plotted on the x-axis and explains 43% of the total variance, while principal component 2 (PC2) is plotted on the y-axis and explains 35% of the total variance. b, Heatmap of sample-to-sample distances calculated from normalized feature counts across all samples. Color intensity represents the relative distance between samples, providing an assessment of sample similarity and clustering among experimental groups. c, Dispersion estimates for all detected genes. The x-axis represents mean normalized counts and the y-axis represents dispersion estimates. Black points indicate gene-wise dispersion estimates, the red line shows the fitted dispersion trend, and blue points represent the final dispersion estimates used for differential expression analysis. d, Histogram of p-values generated from differential expression testing. The x-axis shows p-values ranging from 0 to 1, and the y-axis indicates the frequency of genes within each p-value bin. The distribution provides an overview of the statistical significance profile across all tested genes. e, Uncropped MA plot comparing gene expression between conditions Sxl knockdown compared and RNAi controls. The x-axis represents mean normalized counts, and the y-axis represents log2 fold change. Each point corresponds to a gene, with blue-highlighted points indicating genes exhibiting statistically significant transcriptional changes between the two conditions. (PDF) [file pbio.3003863.s053.pdf]

a

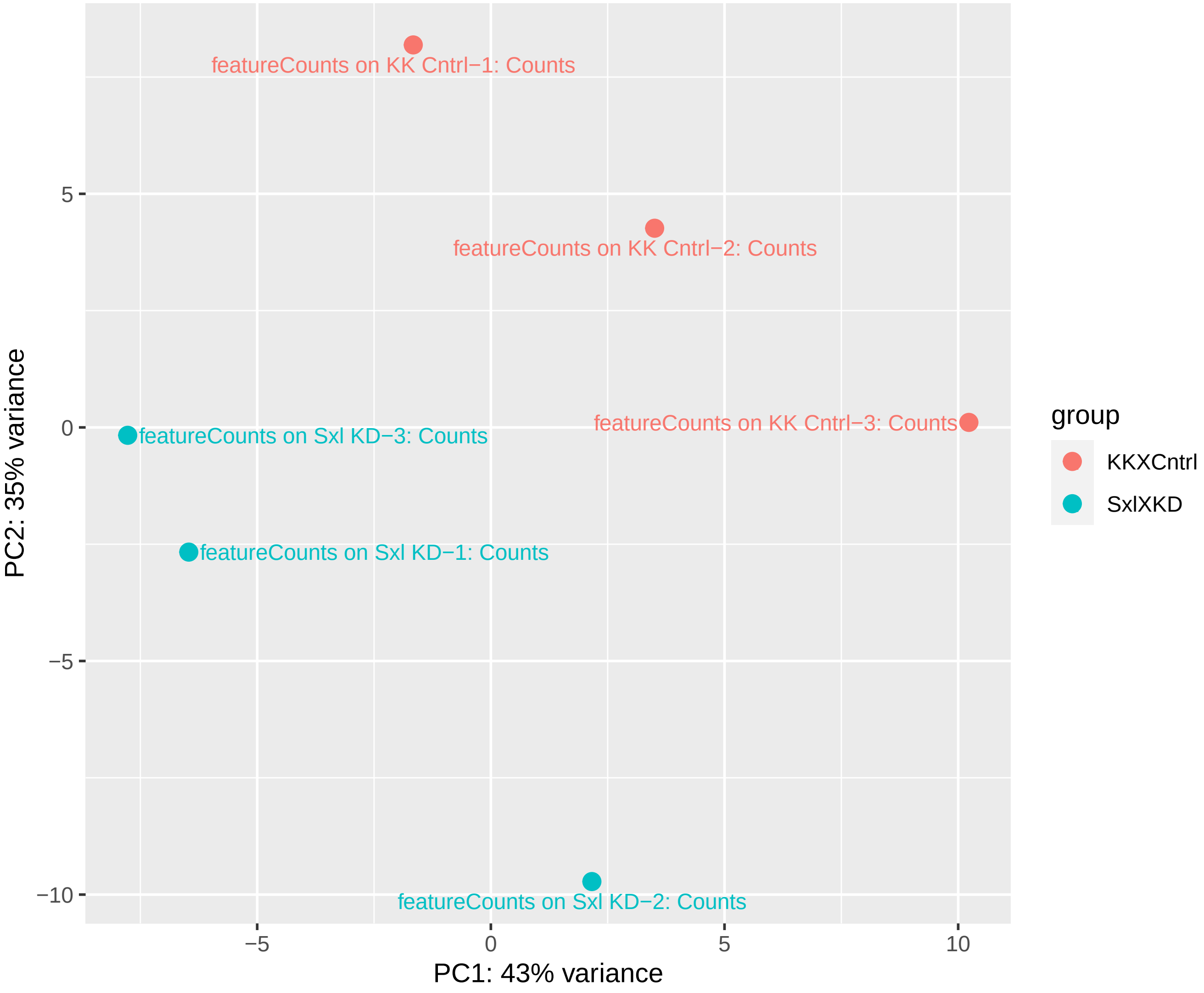
featureCounts on KK Cntrl−1: Counts
featureCounts on KK Cntrl−2: Counts
featureCounts on KK Cntrl−3: Counts
featureCounts on Sxl KD−3: Counts
featureCounts on Sxl KD−1: Counts
featureCounts on Sxl KD−2: Counts
5
0
−5
−10
−5
0
5
10
PC1: 43% variance
PC2: 35% variance
group
KKXCntrl
SxlXKD

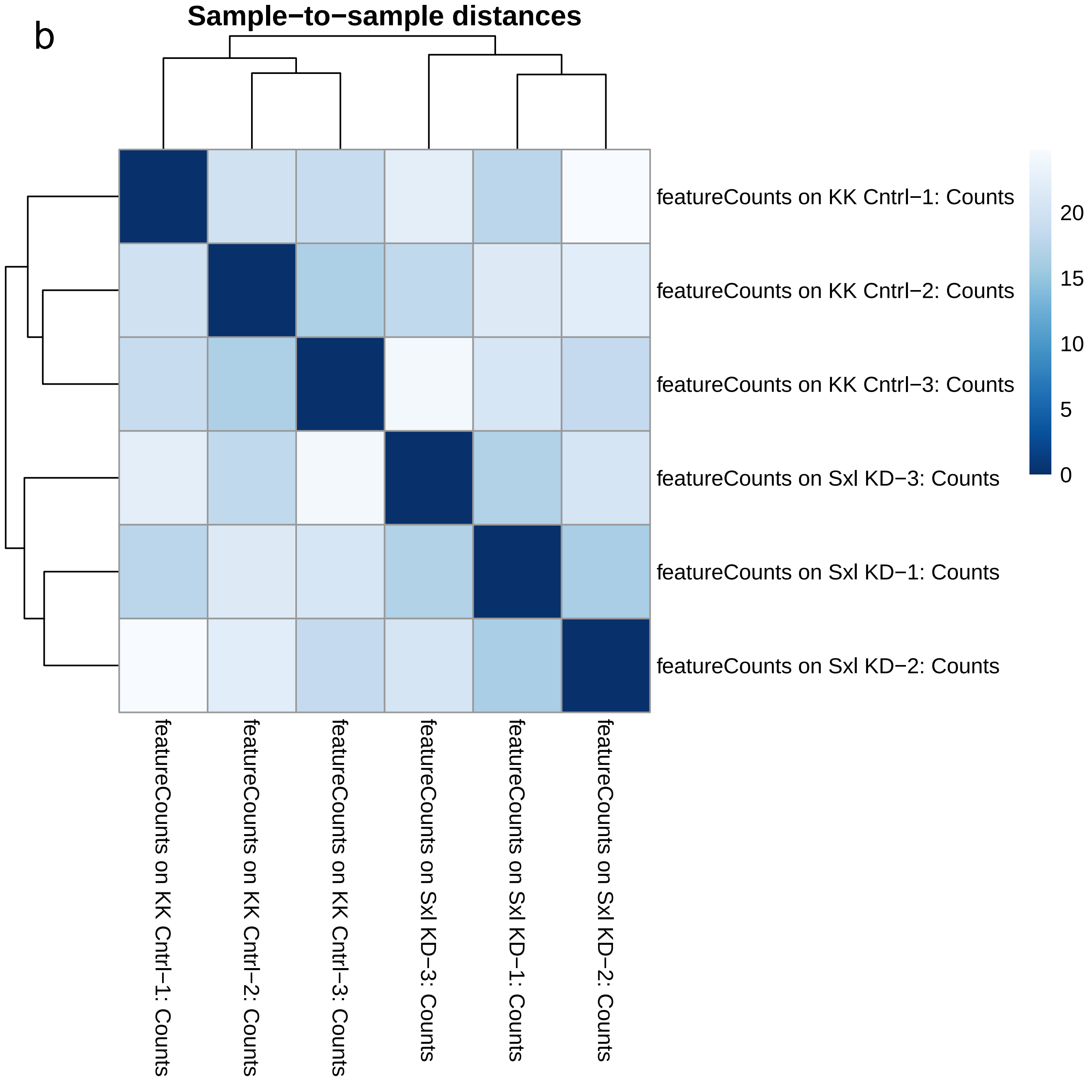
b
Sample−to−sample distances
featureCounts on KK Cntrl−1: Counts
featureCounts on KK Cntrl−2: Counts
featureCounts on KK Cntrl−3: Counts
featureCounts on Sxl KD−3: Counts
featureCounts on Sxl KD−1: Counts
featureCounts on Sxl KD−2: Counts
20
15
10
5
0

c

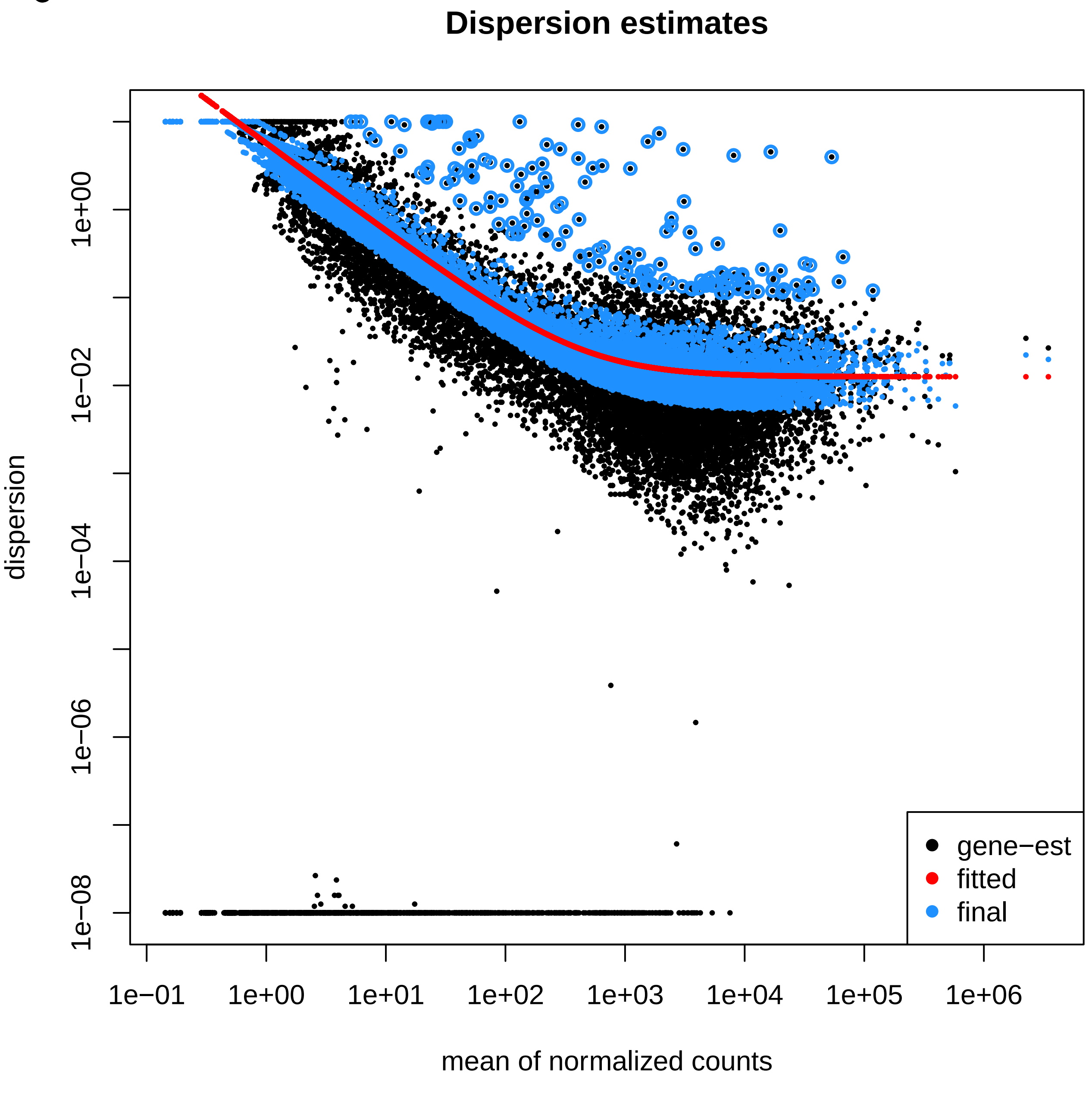
Dispersion estimates
dispersion
1e+00
1e−02
1e−04
1e−06
1e−08
mean of normalized counts
1e−01
1e+00
1e+01
1e+02
1e+03
1e+04
1e+05
1e+06
gene−est
fitted
final

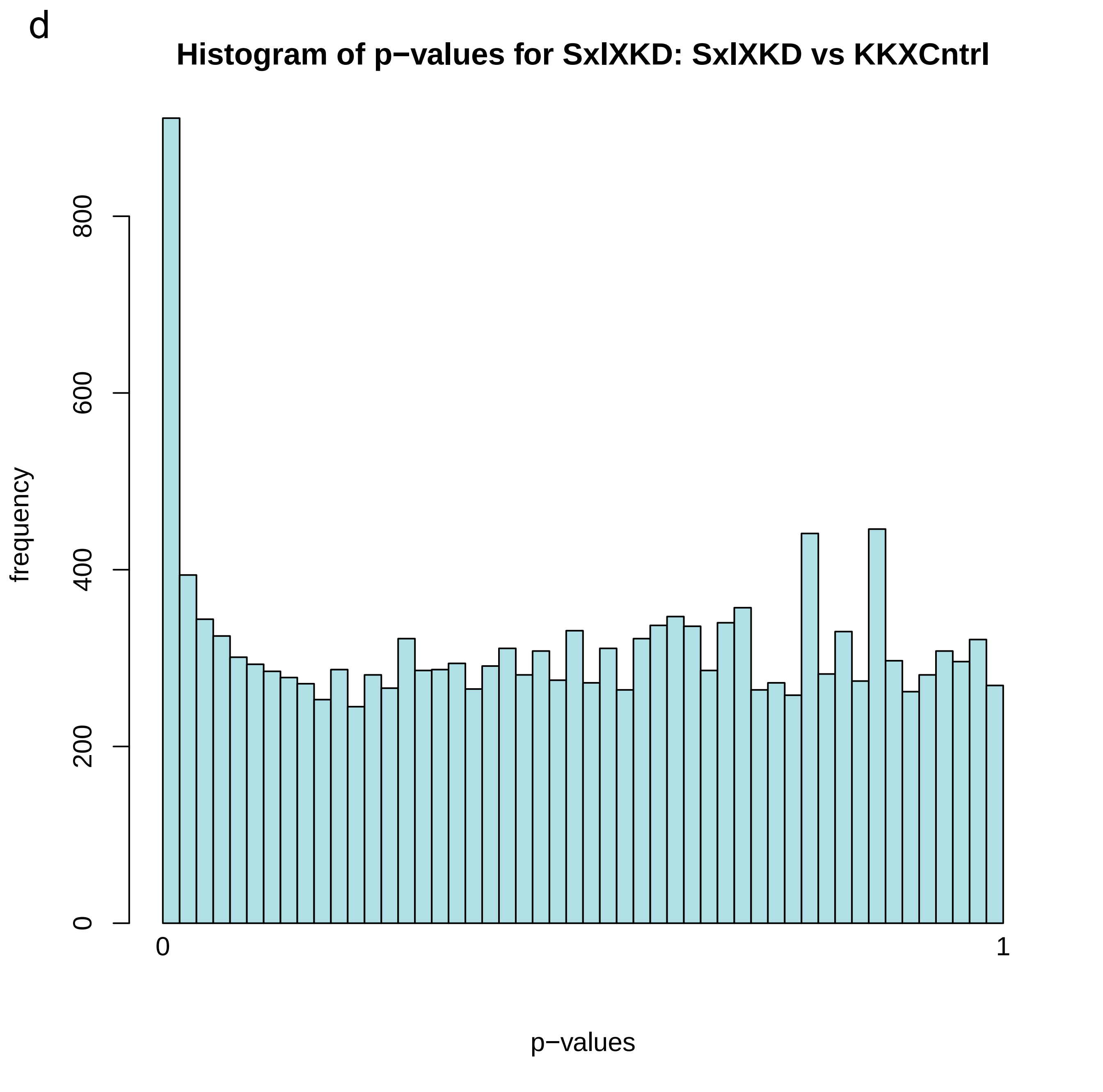
d
Histogram of p−values for SxlXKD: SxlXKD vs KKXCntrl
frequency
0
200
400
600
800
0
1
p−values

e

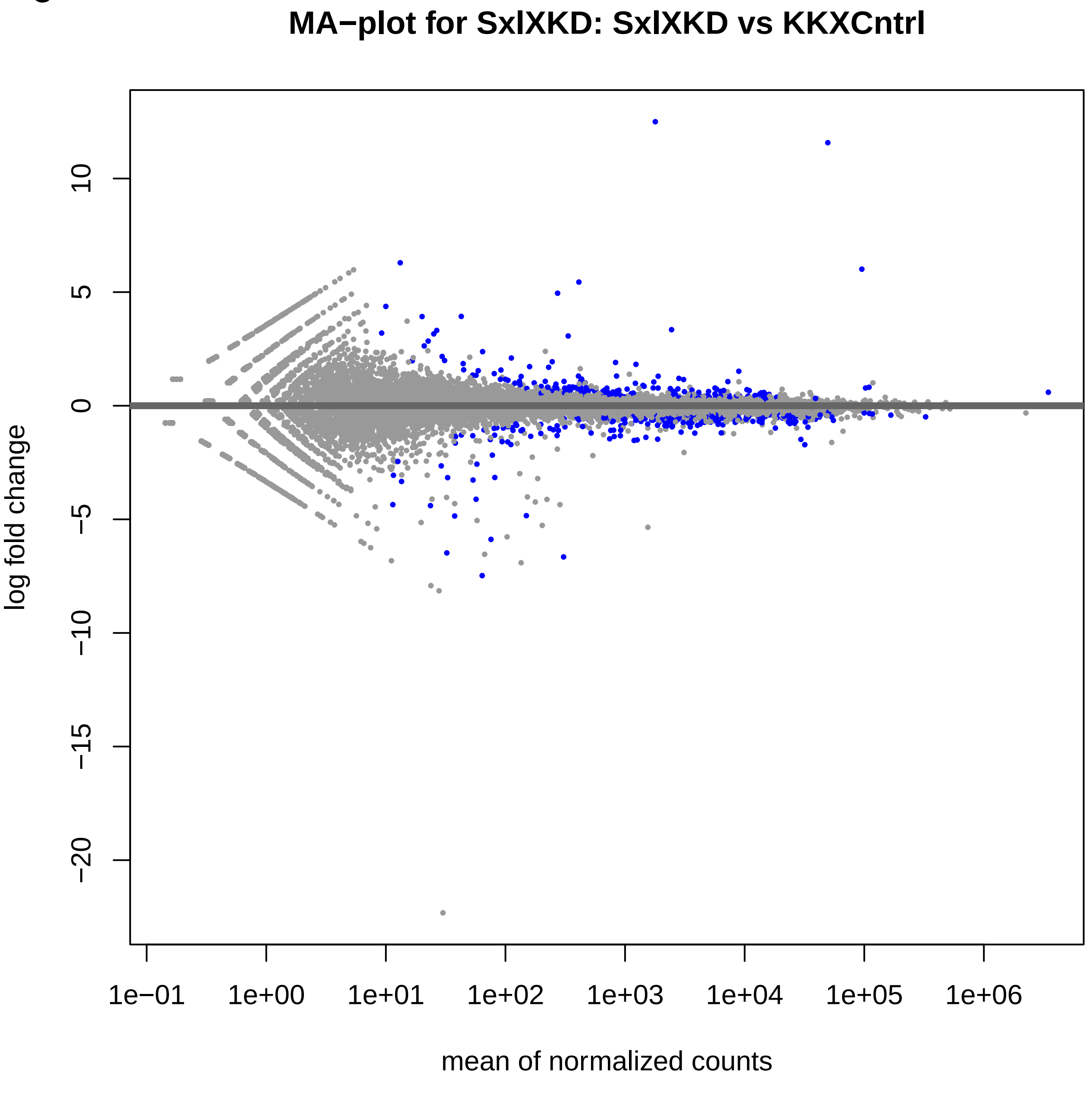
MA−plot for SxlXKD: SxlXKD vs KKXCntrl
log fold change
10
5
0
−5
−10
−15
−20
1e−01
1e+00
1e+01
1e+02
1e+03
1e+04
1e+05
1e+06
mean of normalized counts
