# Supplementary material for: Neuronal protein Sex-lethal modulates tRNA synthesis via the polymerase III subunit Polr3E in male Drosophila neurons
Source: PLoS Biol. 2026 Jul 17;24(7):e3003863. doi: 10.1371/journal.pbio.3003863 (PMC13432123; doi:10.1371/journal.pbio.3003863)
Supplement: S17 Fig — a, Principal component analysis (PCA) of variance-stabilized gene expression data showing sample clustering by experimental condition (SxlRAC (blue) and mCherry controls (BDSC #35787, red)). Principal component 1 (PC1) is plotted on the x-axis and explains 40% of the total variance, while principal component 2 (PC2) is plotted on the y-axis and explains 22% of the total variance. b, Heatmap of sample-to-sample distances calculated from normalized feature counts across all samples. Color intensity represents the relative distance between samples, providing an assessment of sample similarity and clustering among experimental groups. c, Dispersion estimates for all detected genes. The x-axis represents mean normalized counts and the y-axis represents dispersion estimates. Black points indicate gene-wise dispersion estimates, the red line shows the fitted dispersion trend, and blue points represent the final dispersion estimates used for differential expression analysis. d, Histogram of p-values generated from differential expression testing. The x-axis shows p-values ranging from 0 to 1, and the y-axis indicates the frequency of genes within each p-value bin. The distribution provides an overview of the statistical significance profile across all tested genes. e, Uncropped MA plot comparing gene expression between conditions SxlRAC and mCherry controls. The x-axis represents mean normalized counts, and the y-axis represents log2 fold change. Each point corresponds to a gene, with blue-highlighted points indicating genes exhibiting statistically significant transcriptional changes between the two conditions. (PDF) [file pbio.3003863.s060.pdf]

a

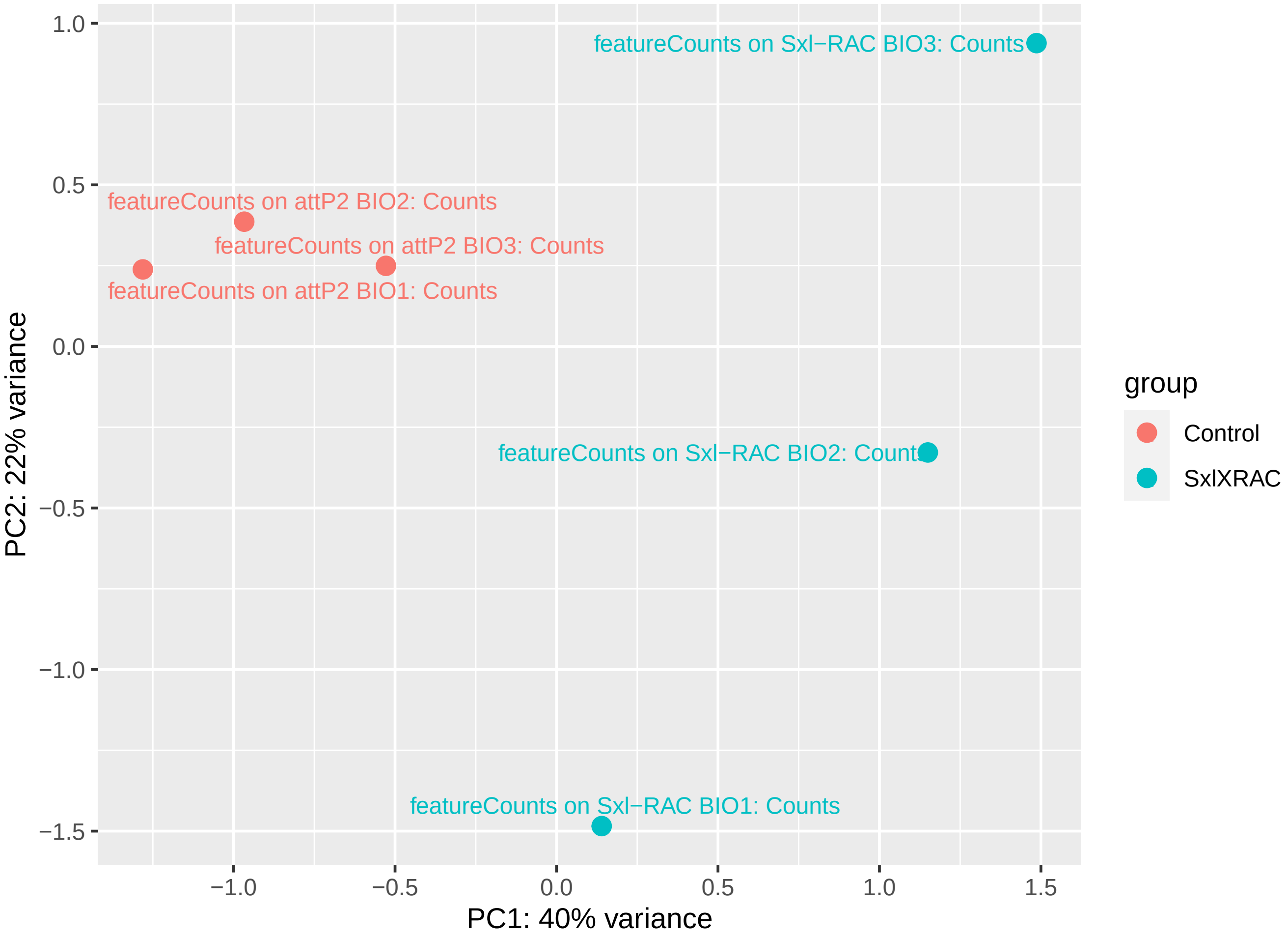

featureCounts on Sxl−RAC BIO3: Counts
featureCounts on attP2 BIO2: Counts
featureCounts on attP2 BIO3: Counts
featureCounts on attP2 BIO1: Counts
featureCounts on Sxl−RAC BIO2: Counts
featureCounts on Sxl−RAC BIO1: Counts
1.0
0.5
0.0
−0.5
−1.0
−1.5
−1.0
−0.5
0.0
0.5
1.0
1.5
PC1: 40% variance
PC2: 22% variance
group
Control
SxlXRAC

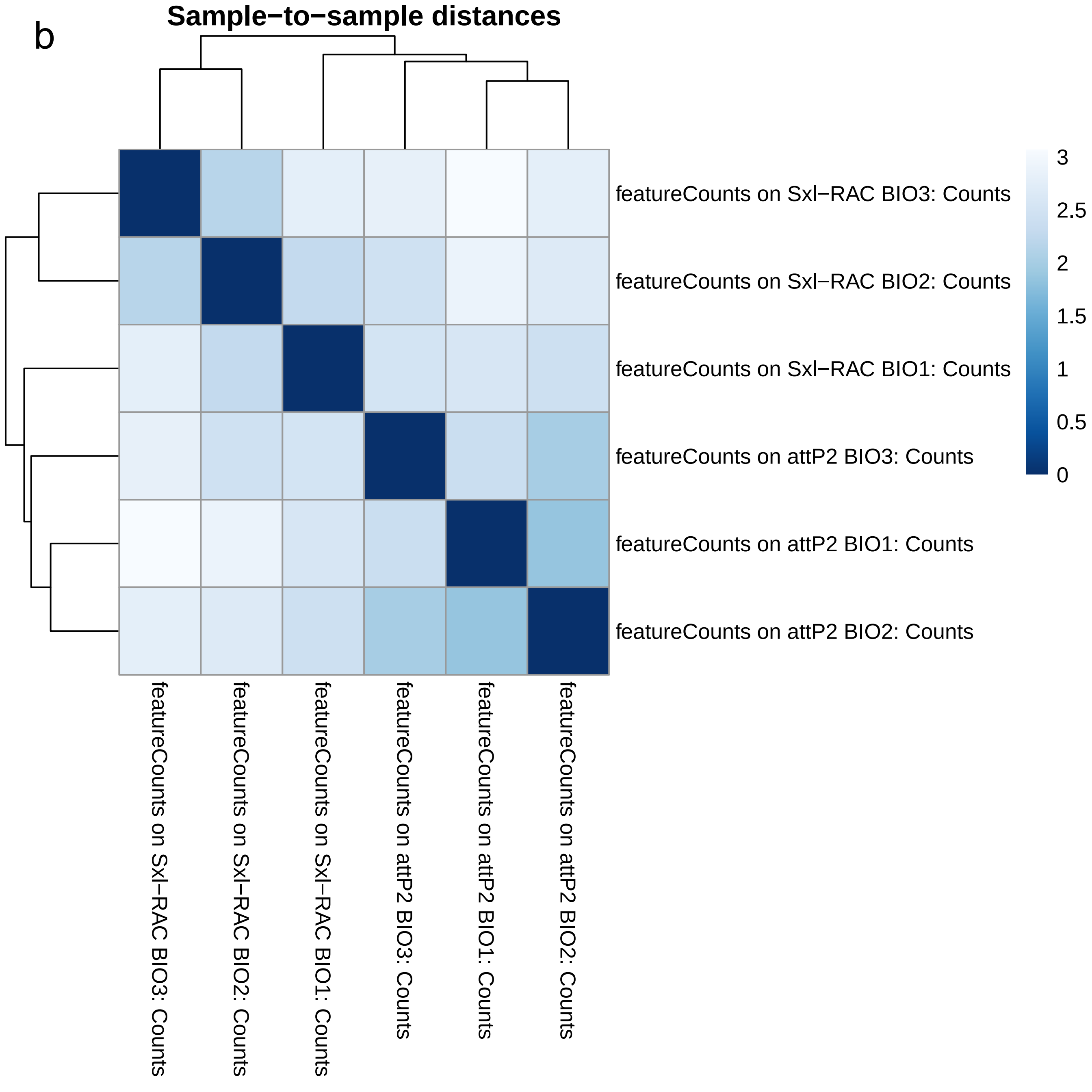
b
Sample−to−sample distances
featureCounts on Sxl−RAC BIO3: Counts
featureCounts on Sxl−RAC BIO2: Counts
featureCounts on Sxl−RAC BIO1: Counts
featureCounts on attP2 BIO3: Counts
featureCounts on attP2 BIO1: Counts
featureCounts on attP2 BIO2: Counts
3
2.5
2
1.5
1
0.5
0

c

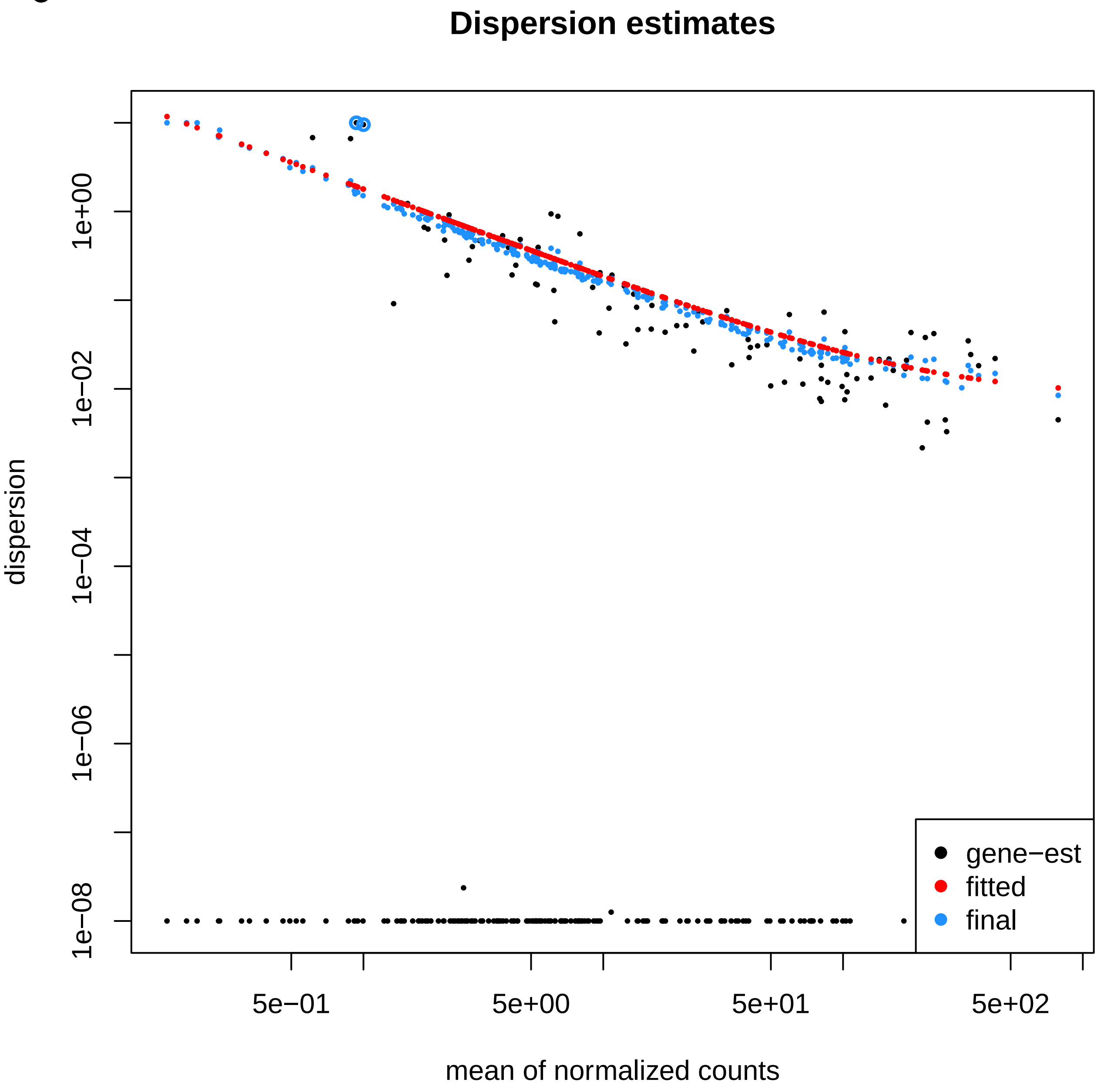
Dispersion estimates
dispersion
1e+00
1e−02
1e−04
1e−06
1e−08
mean of normalized counts
5e−01
5e+00
5e+01
5e+02
gene−est
fitted
final

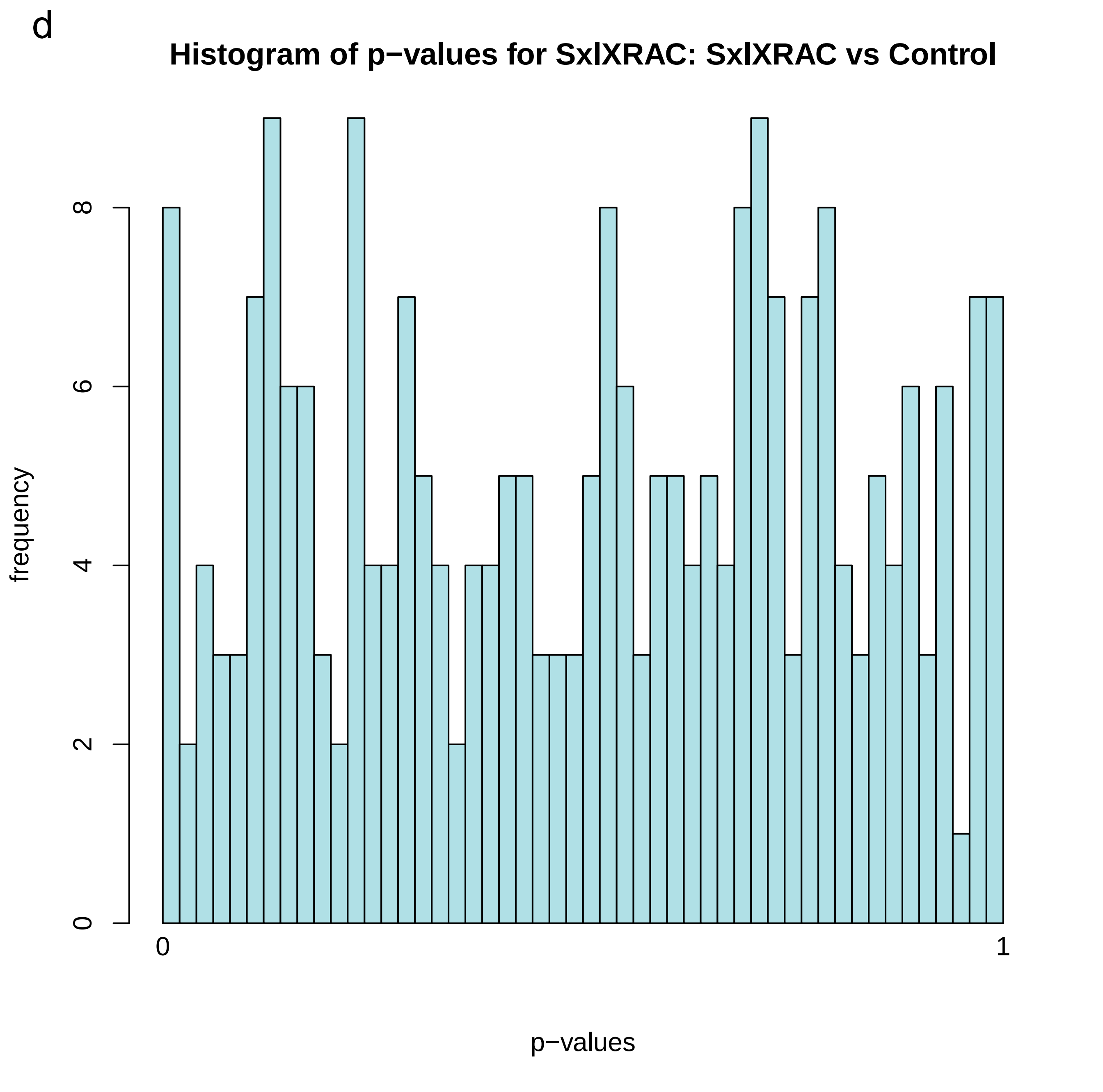

d
Histogram of p−values for SxlXRAC: SxlXRAC vs Control
frequency
0
2
4
6
8
0
1
p−values

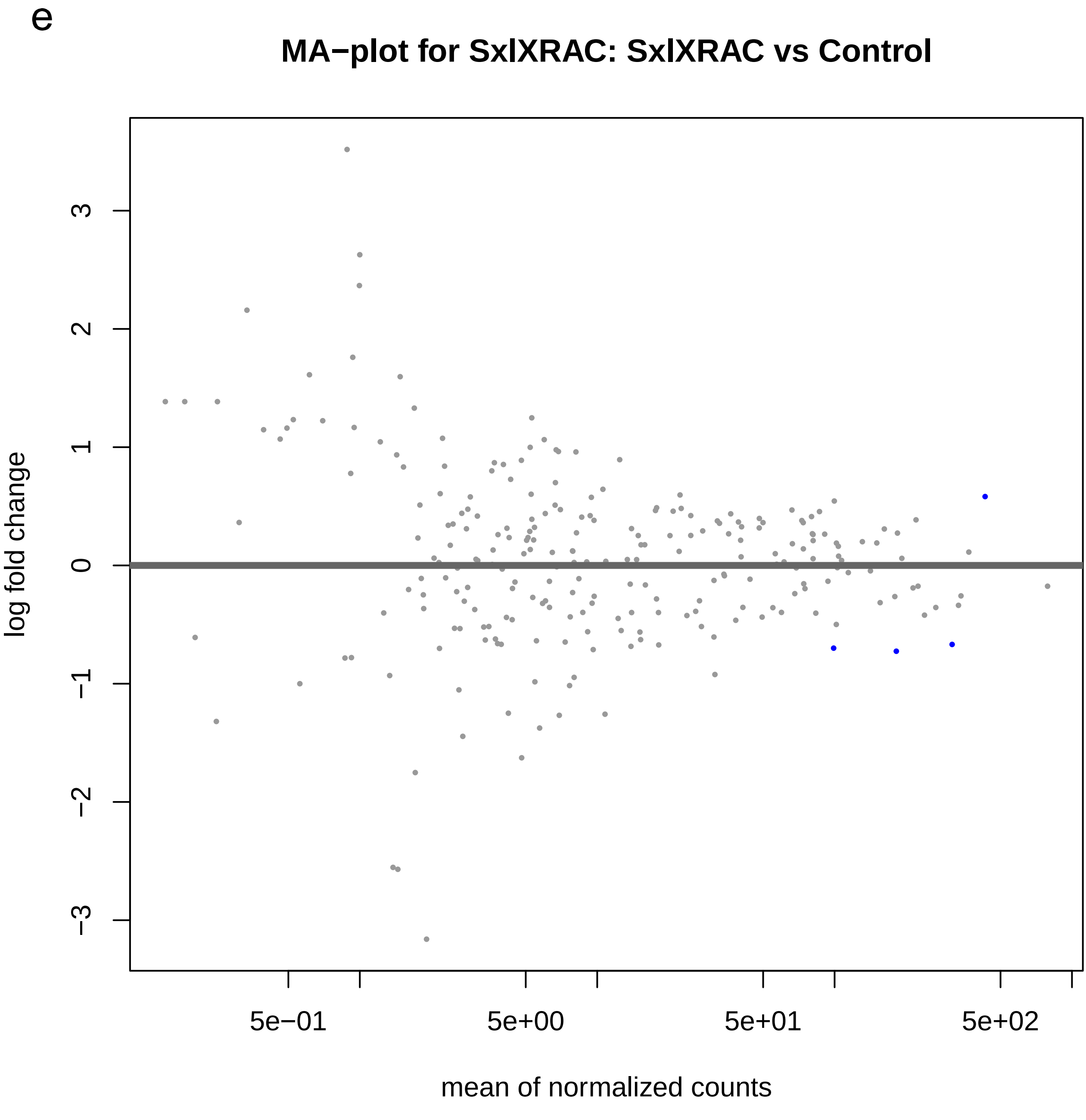

e
MA−plot for SxlXRAC: SxlXRAC vs Control
log fold change
3
2
1
0
−1
−2
−3
5e−01
5e+00
5e+01
5e+02
mean of normalized counts
