# Supplementary material for: Neuronal protein Sex-lethal modulates tRNA synthesis via the polymerase III subunit Polr3E in male Drosophila neurons
Source: PLoS Biol. 2026 Jul 17;24(7):e3003863. doi: 10.1371/journal.pbio.3003863 (PMC13432123; doi:10.1371/journal.pbio.3003863)
Supplement: Attachment — Submitted filename: 157578_1_rebuttal_3448522_szjvj8.pdf [file pbio.3003863.s063.pdf]

***Reviewer #1 (Evidence, reproducibility and clarity (Required)):***

*As stated by the authors in the introduction, the RNA-binding protein Sxl is foundational to understanding sex determination in Drosophila. Sxl has been extensively studied as the master regulator of female sex determination in the soma, where it is known to initiate an alternative splicing cascade leading to the expression of DsxF. Additionally, Sxl has been shown to be responsible for keeping X chromosome dosage compensation off in females, while males hyperactivate their X chromosome. While these roles have been well defined, the authors explore an aspect of Sxl that is quite separate from its role as master regulator of female fate. They describe Sxl-RAC, a Sxl isoform that is expressed in the male and female nervous system. Using several genomic techniques, the authors conclude that the Sxl-RAC isoform associates with chromatin in a similar pattern to the RNA polymerase II/III subunit, Polr3E, and Sxl depends on Polr3E for chromatin-association. Further, neuronal loss of Sxl causes changes in lifetime and geotaxis in a similar manner as loss of Polr3E. The work is thorough and significant and should be appropriate for publication if a few issues can be addressed.*

***Major Concerns:***

*1) How physiological is the Sxl chromatin-association assay? As binding interactions are concentration-dependent, how similar is Sxl-DAM expression to wt Sxl expression in neurons? In addition, does the Sxl-DAM protein function as a wt Sxl protein? Does UAS-Sxl-DAM rescue any Sxl loss phenotypes?*

> **Author response:**
>
> As Reviewer 3 correctly notes, Targeted DamID relies on ribosomal re-initiation (codon slippage) to produce only trace amounts of the Dam-fusion protein. By design, this results in expression levels that are **significantly lower** than those of the endogenous protein. As such, the experiment can be interpreted within a **near–wild-type** context, rather than as an overexpression model. The primary aim of this experiment was to determine whether Sxl associates with chromatin, and our dataset provides clear evidence supporting such binding.

*2) Is Polr3E chromatin-association also dependent on Sxl? They should do the reciprocal experiment to their examination of Sxl chromatin-association in Polr3E knockdown. This might also help address point 1-if wt Sxl is normally required for aspects of Polr3E chromatin binding, then concerns about whether the Sxl-DAM chromatin-association is real or artifactual would be assuaged.*

> **Author response:**
>
> This is an interesting thought, however, if Sxl were required for Polr3E recruitment to RNA Pol III, then, in most male *Drosophila melanogaster* cells, Polr3E would not be incorporated, and **males would not be viable** (as it is essential for Pol III activity). While

it is possible that there could be a subtle effect on Polr3E recruitment, such an experiment, would **not alter the central conclusion** of our study - that Sxl is recruited to chromatin (accessory to the Pol III complex) via Polr3E.

***Minor concerns:***

*The observed Sxl loss of function phenotypes are somewhat subtle (although perhaps any behavior phenotype at all is a plus). Did they try any other behaviour assays-courtship, learning/memory, anything else at all to test nervous system function?*

**Author response:**

Given the **exploratory nature** of this study, we focused on broader behavioural and transcriptional assays.

*While well written, it is sometimes difficult to understand how the experiment was performed or what genotypes were used without looking into the methods sections. One example is they should describe the nature of the Sxl-DAM fusion protein clearly in the results.*

**Author response:**

We will revise these sections to improve clarity and ensure there is no confusion.

***Reviewer #1 (Significance (Required)):***

*This manuscript represents a dramatic change in our thinking about the action of the Sex-lethal protein. Previously, Sxl was known as the master regulator of both sex determination and dosage compensation, and performed these roles as an RNA-binding protein affecting RNA splicing and translational regulation. Here, the authors describe a sex-non-specific role of Sxl in the male and female nervous system. Further, this activity appears independent of Sxl's RNA binding activity and instead Sxl functions as a chromatin-associating protein working with the RNA pol2/3 factor Polr3E to regulate gene expression. Thus, this represents a highly significant finding.*

***Reviewer #2 (Evidence, reproducibility and clarity (Required)):***

*Summary:*
*In this paper, the authors report on an unexpected activity for Sex lethal (Sxl) (a known splicing regulator that functions in sex determination and dosage compensation) in binding to chromatin. They show, using DamID, that Sxl binds to approximately the same chromatin*

*regions as Polr3E (a subunit of RNA Pol III). They show that this binding to chromatin is unaffected by mutations in the RNA binding domains or by deletions of either N or C terminal regions of the Sxl protein. This leads the authors to conclude that Sxl must bind to chromatin through some interacting protein working through the central region of the Sxl protein. They show that Sxl binding is dependent on Polr3E function. They show that male-specific neuronal knockdown of Sxl gives similar phenotypes to knockdown of Polr3E in terms of lethality and improved negative geotaxis. They show gene expression changes with knockdown of Sxl in male adult neurons - mainly that metabolic and pigmentation genes go down in expression. They also show that expression of a previously discovered male adult specific form of Sxl (that does not have splicing activity) in the same neurons also leads to changes in gene expression, including more upregulated than downregulated tRNAs. But they don't see (or don't show) that the same tRNA genes are down with knockdown of Sxl. Nonetheless, based on these findings, they suggest that Sxl plays an important role in regulating Pol III activity through the Polr3E subunit.*

***Major comments:***

*To be honest, I'm not convinced that the conclusions drawn from this study are correct. The fact that every mutant form of Sxl shows the same result from the DamID labelling is a little concerning. I would like to see independent evidence of the SxlRac protein binding chromatin.*

*Do antibodies against this form (or any form) of Sxl bind chromatin in salivary gland polytene chromosomes, for example? Does Sxl from other insects where Sxl has no role in sex determination bind chromatin?*

**Author Response:**

Regarding the reviewer’s overall concerns about the legitimacy of the Sxl binding data:

**i)** The fold differences between Dam-Sxl-mutants and the Dam-only control are **very robust** (up to 9 log2 fold change (500-fold change)), which is higher than what we observe with most transcription factors using Targeted DamID.

**ii)** We observed that Sxl binding was significantly reduced upon knockdown of Polr3E, confirming that the signal we observe is **biologically specific** and not due to technical noise or background.

**iii)** If the concern relates to potential Sxl binding in non-neuronal tissues such as salivary glands, we would like to clarify that all DamID constructs were expressed under ***elav*-GAL4**, a pan-neuronal driver. Furthermore, dissections were performed to isolate larval brains, with **salivary glands carefully removed**. This ensures that chromatin profiles were derived from neuronal tissue exclusively.

**iv)** Salivary gland polytene chromosome staining with a Sxl antibody in a closely related species (*Drosophila virilis*) show **binding of Sxl to chromatin** in both sexes (Bopp et al., 1996).

We will include more text in the revised manuscript to emphasise these points.

*Do antibodies against this form (or any form) of Sxl bind chromatin in salivary gland polytene chromosomes, for example? Does Sxl from other insects where Sxl has no role in sex determination bind chromatin?*

**Author Response:**

Prior work in *Drosophila virilis* (where Sxl is also required for sex determination and Sxl-RAC is conserved) has already demonstrated **Sxl-chromatin association** (using a full-length Sxl antibody) in salivary glands using polytene chromosome spreads (Bopp et al., 1996). Binding is observed in **both sexes and across the genome**, reflecting our observations. We will incorporate this into the revised discussion to support the chromatin-binding role of Sxl across species.

There is a clear and long-overlooked **precedent** for *Sxl*'s alternative, sex-independent roles, findings that have been largely overshadowed by the gene's canonical function. Our study not only **validates** and extends these observations but also brings much-needed attention to this understudied aspect of fundamental biology.

Bopp D, Calhoun G, Horabin JI, Samuels M, Schedl P. Sex-specific control of Sex-lethal is a conserved mechanism for sex determination in the genus Drosophila. Development. 1996 Mar;122(3):971-82. doi: 10.1242/dev.122.3.971. PMID: 8631274.

*I would like to see independent evidence of the SxlRac protein binding chromatin.*

**Author Response:**

We do not believe this is necessary:

**i)** Our data demonstrated that a large **N-terminal truncation** of Sxl (removing far more of the N-terminal region than is absent in Sxl-RAC) does not impair chromatin binding.

**ii)** Our deletion experiments show that it is the **central domain** of Sxl that is required for chromatin association (as removal of the N-or C-terminal domain has no effect). This central domain is **unaffected** in Sxl-RAC.

**iii)** Independent Y2H experiments have shown that it is exclusively the **RBD-1** (RNA binding domain 1) of the central domain of Sxl that interacts with Polr3E (Dong et al., 1999). Sxl-RAC contains this region, therefore will be recruited by Polr3E.

**iv)** Review 3 also believes that this is not necessary (see cross-review below) and highlights the **robustness** of the Y2H experiments performed by Dong et al., 1999.

*Also, given that their DamID experiments reveal that Sxl binds half of the genes encoded in the Drosophila genome, finding that it binds around half of the tRNA genes is perhaps not surprising.*

**Author Response:**

Our data show that Sxl binds to a range of Pol III-transcribed loci, and this binding pattern supports the proposed model that **Sxl plays a broader regulatory role in Pol III activity**. Within these Pol III targets, tRNA genes represent a specific and biologically relevant subset. The emphasis on tRNAs is not to suggest they are the exclusive or primary targets of Sxl, but rather to **highlight a functionally important class of Pol III-transcribed elements** that align with the model we are proposing. We will revise the text to better reflect this framing and avoid any confusion regarding the scope of Sxl's binding profile.

*I would like to see evidence beyond citing a 1999 yeast two-hybrid study that Sxl and Polr3E directly interact with one another.*

**Author response:**

We do not believe this is necessary (these points were also mentioned above):

**i)** The Dong et al., 1999 study was **highly comprehensive** in its characterisation of Sxl binding to Polr3E.

**ii)** Our DamID data provide strong complementary evidence for this interaction: knockdown of *Polr3E* robustly reduces *Sxl*'s recruitment to chromatin, strongly supporting the relevance of the interaction *in vivo*.

**iii)** Review 3 highlights the robustness of the Y2H experiments performed by Dong et al., 1999.

*In my opinion, the differences in lethality observed with loss of Sxl versus control are unlikely to be meaningful given the different genetic backgrounds.*
*The similar defects in negative geotaxis could be meaningful, but I'm unsure how often this phenotype is observed. What other class of genes affect negative geotaxis? It's a little unclear why having reduced expression of metabolic and pigment genes or of tRNAs would improve neuronal function.*

**Author response:**

While the differences in survival were indeed subtle, they were statistically significant and thus warranted inclusion. Our primary aim in this section was to demonstrate that knockdown of *Sxl* or *Polr3E* results in **comparable behavioural and transcriptional phenotypes**, suggesting overlapping functional roles. In this context, we believe the data were presented transparently and effectively support our interpretation.

Regarding the negative geotaxis phenotype, we appreciate the reviewer's interest and agree that it is both **intriguing and atypical**. For this reason, we performed the assay **multiple times**, particularly in *Polr3e* knockdowns, to confirm the robustness of the

result. To address potential confounding variables, we **carefully selected control lines** that account for genetic background and transgene insertion site, including KK controls and attP40-matched lines. We also employed multiple independent RNAi lines targeting *Sxl* to validate the phenotype across different genetic backgrounds.

Although the observed improvement in climbing is unexpected, it is not without **precedent** in the RNA polymerase III field. Notably, Malik et al. (2024) demonstrated that heterozygous *Polr3D*$^{EY/+}$ mutants exhibit a significantly delayed decline in climbing ability with age. We allude to this in the discussion and will revise the text to emphasise this connection more explicitly.

Finally, while we recognise that negative geotaxis is a relatively broad assay and thus does not pinpoint the precise cellular mechanisms involved, we interpret the phenotype as suggesting a **neural basis** and a functional role for *Sxl* in the nervous system.

*One would expect that not just the same classes of genes would be affected by loss and overexpression of Sxl, but the same genes would be affected - are the same genes changing in opposite directions in the two experiments or just the same classes of genes. Likewise, are the same genes changing expression in the same direction with both Sxl and the Polr3E loss? Also, why are tRNA genes not also affected with Sxl loss. Finally, they describe the changes in gene expression as being in male adult neurons, but the sequencing was done of entire heads - so no way of knowing which cell type is showing differential gene expression.*

**Author response:**

While we do examine gene classes, our approach also includes **pairwise correlation** analyses of gene expression changes between specific genotypes. Notably, we observed a **significant positive correlation** between *Polr3e* knockdowns and *Sxl* knockdowns, and a **significant negative correlation** between Sxl-RAC–expressing flies and *Sxl* knockdowns. Furthermore, we examined Sxl-DamID target genes within our RNA-seq datasets and found a consistent relationship between Sxl targets and genes differentially expressed in *Polr3e* knockdowns.

Regarding the Pol III qPCR results, we note that tRNA expression changes may require a **longer duration** of RNAi induction (e.g., beyond 4 days) to become apparent, especially given that phenotypic effects such as changes in lifespan and negative geotaxis only emerge after **20 days** or more. It is also plausible that *Sxl* knockdown leads to a partial reduction in Pol III efficiency, which may not be readily detectable through bulk Pol III qPCRs. We are willing to repeat Pol III qPCRs at later timepoints to further investigate this trend.

Finally, we infer that gene expression changes observed in our RNA-seq data are of neuronal origin, as all knockdown and overexpression constructs used in this study were **driven pan-neuronally** using *elav-/nSyb*-GAL4. While we acknowledge that bulk RNA-seq does not provide cell-type resolution, tissue-specific assumptions are widely used in the field when driven by a relevant promoter.

*I'm also not sure what I'm supposed to be seeing in panel 5F (or in the related supplemental*

*figure) and if it has any meaning - If they are using the Sxl-T2A-Gal4 to drive mCherry, I think one would expect to see expression since Sxl transcripts are made in both males and in females. Also, one would expect to see active protein expression (OPP staining) in most cells of the adult male brain and I think that is what is observed, but again, I'm not sure what I'm supposed to be looking at given the absence of any arrows or brackets in the figures.*

**Author Response:**

Due to the presence of the **T2A tag** and the premature stop codon in exon 3 of early male *Sxl* transcripts, GAL4 expression is not expected in males unless the head-specific $Sxl^{RAC}$ isoform is produced. The aim of panel 5F is to demonstrate the **spatial overlap** between $Sxl^{RAC}$ expression (as we are examining male brains) and regions of elevated protein synthesis, as detected by OPP staining.

To quantitatively assess this relationship, we performed **colocalisation analysis** using ImageJ, which showed a positive correlation between *Sxl* and OPP signal intensity, supporting this interpretation. It is also evident from our images that regions with lower levels of protein synthesis (such as the neuropil - as shown in independent studies Villalobos-Cantor et al., 2023) **concurrently lack *Sxl*-related signal**. We have highlighted regions in Fig. 5 exhibiting higher/lower levels of *Sxl*/OPP signal to better illustrate this relationship. We can also test the effects of knockdown/overexpression on general protein synthesis if required.

Villalobos-Cantor S, Barrett RM, Condon AF, Arreola-Bustos A, Rodriguez KM, Cohen MS, Martin I. Rapid cell type-specific nascent proteome labeling in *Drosophila*. Elife. 2023 Apr 24;12:e83545. doi: 10.7554/eLife.83545. PMID: 37092974; PMCID: PMC10125018.

***Minor comments:***

*Line 223 - 225 - I believe that it is expected that Sxl transcripts would be broadly expressed in the male and female adult, given that it is only the spliced form of the transcript that is female specific in expression.*

As explained above, **the only isoform that will be 'trapped' by the T2A-GAL4 in males is the Sxl-RAC isoform** (as the other isoforms contain premature stop codons). Our immunohistochemistry data indicate that *Sxl-RAC* is expressed in the male brain, specifically in neurons. Therefore, knockdown experiments in males will reduce all mRNA isoforms, of which, Sxl-RAC is the only one producing a protein.

*Line 236 - 238 - Sentence doesn't make sense.*

We have addressed and clarified this.

***Reviewer #2 (Significance (Required)):***

*It would be significant to discover that a gene previously thought to function in only sex determination and dosage compensation also moonlights as a regulator of RNA polymerase III activity. Unfortunately, I am not convinced by the work presented in this study that this is the case.*

*My expertise is in Drosophila biology, including development, transcription, sex determination, morphogenesis, genomics, transcriptomics, DNA binding*

***Reviewer #3 (Evidence, reproducibility and clarity (Required)):***

*Storer, McClure and colleagues use genome-wide DNA-protein binding assays, transcriptomics, and genetics to work out that Drosophila Sxl, widely known as an RNA-binding protein which functions as a splicing factor to determine sex identity in Drosophila and related species, is also a chromatin factor that can stimulate transcription by Pol III and Pol II of genes involved with metabolism and protein homeostasis, specifically some encoding tRNAs.*

*The evidence for the tenet of the paper -- that Sxl acts as a chromatin regulator with Polr3E, activating at least some of its targets with either Pol III or Pol II -- is logical and compelling, the paper is well written and the figures well presented. Of course, more experiments could always be wished for and proposed, but I think this manuscript could be published in many journals with just a minor revision not involving additional experiments. I have a few specific comments below, all minor.*

***Scientific points:***

*- The approach taken for the evaluation of Sxl DNA-binding activity in Fig2 is not entirely clear. I assume these are crosses of elav-Gal4 x different UAS- lines, then using males or females for UAS-Sxl-Full-Length. But what about the others? Were the experiments done in males only? This is hinted at in the main text but not explicitly indicated in the figure or the methods (at least, that I could easily find). And is this approach extended to all other experiments? Longevity? Climbing assays? Considering the role of Sxl, it may be helpful to be fastidiously systematic with this.*

**Author Response:**

We have revised the wording to ensure greater clarity. Males were used for all survival and behavioural experiments (as only males can be leveraged for knocking down Sxl-RAC without affecting the canonical Sxl-F isoform).

*- In the discussion, lines 360-61, the authors say: Indeed, knockdown of Polr3E leads to a loss of Sxl binding to chromatin, suggesting a cooperative mechanism. Maybe I am misunderstanding the authors, but when I read "cooperation" in this context I think of biochemical cooperative binding. This is possible, but I do not think a simple 'requirement' test can suggest specifically that this mechanistic feature of biochemical binding is at play. I would expect, for starters, a reciprocal requirement for binding (which is not tested), and some quantitative features that*

*would be difficult to evaluate in vivo. I do not think cooperative binding needs to be invoked anyway, as the authors do not make any specific point or prediction about it. But if they do think this is going on, I think it would need to be referred to as a speculation.*

**Author Response:**

We appreciate that the original wording may have been unclear and will revise the text to more accurately reflect a **functional relationship**, rather than implying direct cooperation.

*- In lines 428-432, the authors discuss the ancestral role of Sxl and make a comparison with ELAV, in the context of an RNA-binding protein that has molecular functions beyond those of a splicing factor, considering the functions of ELAV in RNA stability and translation, and finishing with "suggesting that similar regulatory mechanisms may be at play". I do not understand this latter sentence. Which mechanisms are these? Are the authors referring to the molecular activities of ELAV and SXL? But what would be the similarity? SXL seems to have a dual capacity to bind RNA and protein interactors, which allows it to work both in chromatin-level regulation as well as post-transcriptionally in splicing; but ELAV seems rather to take advantage of its RNA binding function to make it work in multiple RNA-related contexts, all post-transcriptional. I do not see an obvious parallel beyond the fact that RNA binding proteins can function at different levels of gene expression regulation -- but I would not say this parallel are "similar regulatory mechanisms", so I find the whole comparison a bit confusing.*

**Author Response:**

We have reduced this section, as it is largely speculative and intended to highlight potential, though indirect, links in **higher organisms**. Our goal was primarily to illustrate the possibility that *Sxl* may have an ancestral role distinct from its well-characterised function, and to suggest a potential avenue for future research into ELAV2's involvement in chromatin or Pol III regulation.

*- One aspect of the work that I find is missing in the discussion is the possibility that the simultaneous capacity of Sxl for RNA binding and Polr3E binding: are these mutually exclusive? if so, are they competitive or hierarchical? how would they be coordinated anyway?*

**Author Response:**

This is an interesting point, and we have expanded on it further in the Discussion section.

*- The only aspect of the paper where I found that one could make an experimental improvement is the claim that Sxl induces the expression of genes that have the overall effect of stimulating protein synthesis. The OPP experiment shows a correlation between the expression of Sxl and the rate of protein synthesis initiation. However, a more powerful experiment would be, rather*

*obviously, to introduce Sxl knock-down in the same experiment, and observe whether in Sxl-expressing neurons the incorporation of OPP is reduced. I put this forth as a minor point because the tenet of the paper would not be affected by the results (though the perception of importance of the newly described function could be reinforced).*

**Author Response:**

This could be a valid experiment and we are prepared to perform it if required.

*- In a similar way, it would be interesting to know whether the recruitment of Polr3E and Sxl to chromatin is co-dependent or Sxl follows Polr3E. This is also a minor point because this would possibly refine the mechanism of recruitment but does not alter the main discovery.*

**Author Response:**

We have addressed a similar point for Reviewer 2 (see below) and will include a Discussion point for this:

If Sxl were required for Polr3E recruitment to RNA Pol III, then, in most male *Drosophila melanogaster* cells, Polr3E would not be incorporated, and males would not be viable (as it is essential for Pol III activity). While it is possible that there could be a subtle effect on Polr3E recruitment, such an experiment, would not alter the central conclusion of our study - that Sxl is recruited to chromatin (accessory to the Pol III complex) via Polr3E.

***Figures and reporting:***

*- In Figure 2, it would be helpful to see the truncation coordinate for the N and C truncations.*

*- In Figure 3D, genomic coordinates are missing.*

*- In Figure 3E, the magnitude in the Y axis is not entirely clear (at least not to me). How is the amount of binding across the genome quantified? Is this the average amplitude of normalised TaDa signal across the genome? Or only within binding intervals?*

*- Figure S3E-F: it would be interesting to show the degree of overlap between the downregulated genes that are also binding targets (regardless of the outcome).*

*- Figure 5C-E: similarly to Figure S3, it would be interesting to know how the transcriptional effects compare with the binding targets.*

*- Authors use Gehan-Breslow-Wilcoxon to test survival, which is a bit unusual, as it gives more weight to the early deaths (which are rare in most Drosophila longevity experiments). Is there any rationale behind this? It may be even favour their null hypothesis.*

**Author response:**

Thank you for the detailed feedback on our figures. We have **incorporated** the suggested changes.

We agree that examining the overlap between *Sxl* binding sites and transcriptional changes is valuable, and we aimed to highlight this in the pie charts shown in Figures S3 and S5. If the reviewer is suggesting a more explicit quantification of the proportion of *Sxl*-Dam targets with significant transcriptomic changes, we are happy to include this analysis in the final version of the manuscript.

As noted in the Methods, both Gehan–Breslow–Wilcoxon (GBW) and Kaplan–Meier tests were used. The significance in Figure 4a is specific to the GBW test, which we indicated by describing the effect as *mild*. Our focus here is not on the magnitude of survival differences, but on the consistent trends observed in both *Polr3e* and *Sxl* knockdowns.

***Writing and language:***

*- Introduction finishes without providing an outline of the findings (which is fine by me if that is what the authors wanted).*

*- In lines 361-5, the authors say "We speculate that this interaction not only facilitates Pol III transcription but may also influence chromatin architecture and RNA Pol II-driven transcription as observed with Pol III regulation in other organisms". "This interaction" refers to Polr3E-Sxl-DNA interaction and with "Pol III transcription" I presume the authors refer to transcription executed by Pol III. I am not clear about the meaning of the end of the sentence "as observed with Pol III regulation in other organisms". What is the observation, exactly? That Pol III modifies chromatin in Pol II regulated loci, or that Pol III interactors change chromatin architecture?*

*- DPE abbreviation is not introduced (and only used once).*

*- A few typos:*
*Line 41 ...splicing of the Sxl[late] transcripts, which is [ARE?] constitutively transcribed (Keyes et al.,...*
*Line 76 ...sexes but appears restricted to the nervous system [OF] male pupae and adults (Cline et*
*Line 289 ...and S41). To assess any effect [ON]translational output, O-propargyl-puromycin (OPP)o*
*Line 323 ...illustrating that the majority (72%) changes in tRNA levels [ARE] due to upregulation...hi*
*Line 402 ...it was discovered [WE DISCOVERED]*
*Line 792 ...Sxl across chromosomes X, 2 L/R, 3 L/R and 4. The y-axis represents the log[SYMBOL] ratio... This happens in other figure legends as well.*

**Author response:**

Thank you for the detailed feedback, we have clarified and incorporated the suggested changes.

*****Referee Cross-commenting*****

*Reviewer 1 asks how physiological is the Sxl chromatin-association assay. I think the loss of association in Polr3E knock-down and the lack of association of other splicing factors goes a long way into answering this question. It is true that having positive binding data specifically for Sxl-RAC and negative binding data for a deletion mutant of the RMM domain would provide more robust conclusions (see below), but I am not sure it is completely necessary -- though this will depend on which journal the authors want to send the paper to.*

*I think that the comment of reviewer 1 about the levels of expression of Sxl-DAM does not apply here because of the way TaDa works - it relies on codon slippage to produce minimal amounts of the DAM fusion protein, so by construction it will be expressed at much lower levels than the endogenous protein.*

*Reviewer 1 also asks whether Polr3E chromatin-association is also dependent on Sxl, to round up the model and also as a way to address whether Sxl association to chromatin is real. While I agree with this on the former aim (this would be a nice-to-have), I think I disagree on the latter; there is no need for Polr3E recruitment to depend on Sxl for Sxl association to chromatin to be physiologically relevant. Polr3E is a peripheral component of Pol III and unlikely to depend on a factor of restricted expression like Sxl to interact with chromatin. The recruitment of Sxl could well be entirely 'hierarchical' and subject to Polr3E.*

*Revewer 2 is concerned with the fact that every mutant form of Sxl shows the same result from the DamID labelling. I have to agree with this to a point. A deletion mutant of RMM domains would address this. Microscopy evidence in salivary glands would be nice, certainly, but the system may not lend itself to this particular interaction, which might be short-lived and/or weak. I do not immediately see the relevance of the chromatin binding capacity of non-Drosophilidae Sxl -- though it might indicate that the impact of the discovery is less likely to go beyond this group.*

*Reviewer 2 does not find surprising that some tRNA genes (less than half) are regulated by Sxl. I think the value of that observation is just qualitative, as tRNAs are Pol III-produced transcripts, but their point is correct. A hypergeometric test could settle this.*

*Reviewer 2 is concerned that the evidence of direct interaction between Sxl and Polr3E is a single 1999 two-hybrid study. But that paper contains also GST pull-downs that narrow down the specific domains that mediate binding, and perform the binding in competitive salt conditions. I think it is enough. The author team, I think, are not biochemists, so finding the right collaborators and performing these experiments would take time that I am not sure is warranted.*

*Reviewer 2 is also concerned that the longevity assays may not be meaningful due to the difference in genetic backgrounds. This is a very reasonable concern (which I would extend to the climbing assays - any quantitative phenotype is sensitive to genetic background). However, I think the authors here may have already designed the experiment with this in mind - the controls express untargeted RNAi constructs, but I lose track of which one is control of which. This*

*should be clarified in Methods.*

*Other comments are in line, I think, with what I have pointed out and I generally agree with everything else that has been said.*

***Reviewer #3 (Significance (Required)):***

*Drosophila Sxl is widely known as an RNA-binding protein which functions as a splicing factor to determine sex identity in Drosophila and related species. It is a favourite example of how splicing factors and alternative can have profound influence in biology and used cleverly in the molecular circuitry of the cell to enact elegant regulatory decisions.*

*In this work, Storer, McClure and colleagues use genome-wide DNA-protein binding assays, transcriptomics, and genetics to work out that Sxl is also a chromatin factor with an sex-independent, neuron-specific role in stimulating transcription by Pol III and Pol II, of genes involved with metabolism and protein homeostasis, including some encoding tRNAs.*

*This opens a large number of interesting biological questions that range from biochemistry, gene regulation or neurobiology to evolution. How is the simultaneous capacity of binding RNA and chromatin (with the same protein domain, RRM) regulated/coordinated? How did this dual activity evolve and which one is the ancestral one? How many other RRM-containin RNA-binding proteins can also bind chromatin? How is Sxl recruited to chromatin to both Pol II and Pol III targets and are they functionally related? If so, how is the coordination of cellular functions activated through different RNA polymerases taking place and what is the role of Sxl in this? What are the functional consequences to neuronal biology? Does this affect similarly all Sxl-expressing neurons?*

*The evidence for the central tenet of the paper -- that Sxl acts as a chromatin regulator with Polr3E, activating at least some of its targets with either Pol III or Pol II -- is logical and compelling, the paper is well written and the figures well presented. Of course, more experiments could always be wished for and proposed, but I think this manuscript could be published in many journals with just a minor revision not involving additional experiments.*

**Reviewer #4 (Evidence, reproducibility and clarity (Required)):**

*The convincing analysis demonstrates a role for the Drosophila Sex determining gene sex lethal in controlling aspects of transcription in the nervous system independent of its role in splicing. Interaction with an RNA Pol III subunit mediating Sxl association with chromatin and similar knockdown phenotypes strongly support the role of Sxl in the regulation of neuronal metabolism. Given that Sxl is an evolutionary recent acquisition for sex determination, the study may reveal an ancestral role for Sxl.*

*The conclusions are well justified by the datasets presented and I have no issues with the study or the interpretation. Throughout the work is well referenced, though perhaps the authors might take a look at Zhang et al (2014) (PMID: 24271947) for an interesting evolutionary perspective for the discussion.*

**Author Response:**

Thank you for the thoughtful suggestion. We will be sure to incorporate the findings from Zhang et al. regarding the evolution of the sex determination pathway.

*I have some minor comments for clarification:*

*There is no Figure 2b, should be labelled 2 or label TaDa plots as 2b*

*Clarify if Fig 2 data are larval or adult*

*Larval*

*Fig 3d - are these replicates or female and male?*

*Please elaborate on tub-GAL80[ts] developmental defects*

*Fig 4e, are transcriptomics done with the VDRC RNAi line? The VDRC and BDSC RNAi lines exhibit different behaviours - former has "better" survival and Better negative geotaxis, the latter seems to have poorer survival but little geotaxis effect?*

*Fig S3 - volcano plot for Polr3E?*

*Fig S4a - legend says downregulated genes?*

*The discussion should at least touch on the fact that Sxl amorphs (i.e. Sxl[fP7B0] are male viable and fertile, emphasising that the newly uncovered role is not essential.*

**Author Response:**

We agree with the suggestions outlined in the comments and have made the appropriate revisions.

***Reviewer #4 (Significance (Required)):***

*A nonessential role for Sxl in the nervous system independent of sex-determination contributes to better understanding a) the evolution of sex determining mechanisms, b) the role of RNA PolIII in neuronal homeostasis and c) more widely to the neuronal aging field. I think this well-focused study reveals a hitherto unsuspected role for Sxl.*

**Author Amendments:**

The authors are grateful for the overall feedback and have made changes to the text to address reviewer comments. Furthermore, and if required, we are happy to perform the following experiments:

i) tRNA qPCR at later timepoint in Sxl KD flies.
ii) OPP staining in Sxl KD / Sxl-RAC flies.
