# Supplementary material for: Neuronal protein Sex-lethal modulates tRNA synthesis via the polymerase III subunit Polr3E in male Drosophila neurons
Source: PLoS Biol. 2026 Jul 17;24(7):e3003863. doi: 10.1371/journal.pbio.3003863 (PMC13432123; doi:10.1371/journal.pbio.3003863)
Supplement: Attachment — Submitted filename: Storer et al - Reponse to Reviewers.pdf [file pbio.3003863.s065.pdf]

Response to Reviewers - Storer et al.

SUMMARY OF MAIN CHANGES

We have substantially revised the manuscript to address all reviewer concerns. The major changes include:

New Experimental Data:

1. O-propargyl-puromycin (OPP) assays to directly measure protein synthesis in adult male brains (new Figure 6b-e, formerly Figure 5f; Figure S6). These experiments demonstrate that Sxl bidirectionally regulates translational output: neuronal Sxl knockdown significantly reduces OPP incorporation ($p=0.0048$), while SxlRAC overexpression increases it ($p=0.0128$). Furthermore, regions with high Sxl expression exhibit elevated nascent protein synthesis ($R_{coloc}=0.81$), providing direct functional evidence linking Sxl levels to translational capacity.

2. Additional qPCR measurements of pre-tRNA levels (pre-tRNAHis, pre-tRNAIle, pre-tRNALeu) in both Sxl knockdown and SxlRAC overexpression conditions (new Figure S5f-k), strengthening the connection between Sxl manipulation and Pol III-dependent tRNA transcription.

Other revisions:

1. Changed the title to emphasise the nature of tRNA regulation.

2. Small edits to text to ensure flow.

3. Updated all figures with a more 'soothing' colour-scheme. Figures 1-3 are virtually identical.

4. Updated figure legends to match new figures – Figures 4-6.

5. Included new results section and Figure to explain new data (Figure 6).

6. Added additional sections to the discussion, drawing on new results and discussion previous findings in *Drosophila Virilis*.

7. New and updated supplementary Figures S5-S7.

8. Updated supplementary Tables, including new data.

These revisions substantially strengthen the manuscript by providing direct functional evidence connecting Sxl chromatin occupancy to tRNA transcription and translation, with reciprocal effects demonstrating the dosage-sensitive nature of Sxl function in regulating neuronal translational capacity.

POINT-BY-POINT RESPONSES TO REVIEWERS

Reviewer #1 (Evidence, reproducibility and clarity (Required)):

As stated by the authors in the introduction, the RNA-binding protein Sxl is foundational to understanding sex determination in Drosophila. Sxl has been extensively studied as the master regulator of female sex determination in the soma, where it is known to initiate an alternative splicing cascade leading to the expression of DsxF. Additionally, Sxl has been shown to be responsible for keeping X chromosome dosage compensation off in females, while males hyperactivate their X chromosome. While these roles have been well defined, the authors explore an aspect of Sxl that is quite separate from its role as master regulator of female fate. They describe Sxl-RAC, a Sxl isoform that is expressed in the male and female nervous system. Using several genomic techniques, the authors conclude that the Sxl-RAC isoform associates with chromatin in a similar pattern to the RNA polymerase II/III subunit, Polr3E, and Sxl depends on Polr3E for chromatin-association. Further, neuronal loss of Sxl causes changes in lifetime and geotaxis in a similar manner as loss of Polr3E. The work is thorough and significant and should be appropriate for publication if a few issues can be addressed.

Major Concerns:

1) How physiological is the Sxl chromatin-association assay? As binding interactions are concentration-dependent, how similar is Sxl-DAM expression to wt Sxl expression in neurons? In addition, does the Sxl-DAM protein function as a wt Sxl protein? Does UAS-Sxl-DAM rescue any Sxl loss phenotypes?

Author response:

As Reviewer 3 correctly notes, Targeted DamID relies on ribosomal re-initiation (codon slippage) to produce only trace amounts of the Dam-fusion protein. By design, this results in expression levels that are significantly lower than those of the endogenous protein. As such, the experiment can be interpreted within a near–wild-type context, rather than as an overexpression model. The primary aim of this experiment was to determine whether Sxl associates with chromatin, and our dataset provides clear evidence supporting such binding.

2) Is Polr3E chromatin-association also dependent on Sxl? They should do the reciprocal experiment to their examination of Sxl chromatin-association in Polr3E knockdown. This might also help address point 1-if wt Sxl is normally required for aspects of Polr3E chromatin binding, then concerns about whether the Sxl-DAM chromatin-association is real or artifactual would be assuaged.

Author response:

This is an interesting thought, however, if Sxl were required for Polr3E recruitment to RNA Pol III, then, in most male Drosophila melanogaster cells, Polr3E would not be incorporated, and males would not be viable (as it is essential for Pol III activity). While it is possible that there could be a subtle effect on Polr3E recruitment, such an experiment, would not alter the central conclusion of our study - that Sxl is recruited to chromatin (accessory to the Pol III complex) via Polr3E.

Minor concerns:

The observed Sxl loss of function phenotypes are somewhat subtle (although perhaps any behavior phenotype at all is a plus). Did they try any other behaviour assays-courtship, learning/memory, anything else at all to test nervous system function?

Author response:

Given the exploratory nature of this study, we focused on broader behavioural and transcriptional assays.

While well written, it is sometimes difficult to understand how the experiment was performed or what genotypes were used without looking into the methods sections. One example is they should describe the nature of the Sxl-DAM fusion protein clearly in the results.

Author response:

We have revised these sections to improve clarity throughout the manuscript. Specifically, we have clarified genotypes and experimental conditions in the Results section and figure legends, making it easier to follow without constantly referring to Methods.

Reviewer #1 (Significance (Required)):

This manuscript represents a dramatic change in our thinking about the action of the Sex-lethal protein. Previously, Sxl was known as the master regulator of both sex determination and dosage compensation, and performed these roles as an RNA-binding protein affecting RNA splicing and translational regulation. Here, the authors describe a sex-non-specific role of Sxl in the male and female nervous system. Further, this activity appears independent of Sxl's RNA binding activity and instead Sxl functions as a chromatin-associating protein working with the RNA pol2/3 factor Polr3E to regulate gene expression. Thus, this represents a highly significant finding.

Reviewer #2 (Evidence, reproducibility and clarity (Required)):

Summary:
In this paper, the authors report on an unexpected activity for Sex lethal (Sxl) (a known splicing regulator that functions in sex determination and dosage compensation) in binding to chromatin. They show, using DamID, that Sxl binds to approximately the same chromatin regions as Polr3E (a subunit of RNA Pol III). They show that this binding to chromatin is unaffected by mutations in the RNA binding domains or by deletions of either N or C terminal regions of the Sxl protein. This leads the authors to conclude that Sxl must bind to chromatin through some interacting protein working through the central region of the Sxl protein. They show that Sxl binding is dependent on Polr3E function. They show that male-specific neuronal knockdown of Sxl gives similar phenotypes to knockdown of Polr3E in terms of lethality and improved negative geotaxis. They show gene expression changes with knockdown of Sxl in male adult neurons - mainly that metabolic and pigmentation genes go down in expression. They also show that expression of a previously discovered male adult specific form of Sxl (that does not have splicing activity) in the same neurons also leads to changes in gene expression, including more upregulated than downregulated tRNAs. But they don't see (or don't show) that the same tRNA genes are down with knockdown of Sxl. Nonetheless, based on these findings, they suggest that Sxl plays an important role in regulating Pol III activity through the Polr3E subunit.

Major comments:

To be honest, I'm not convinced that the conclusions drawn from this study are correct. The fact that every mutant form of Sxl shows the same result from the DamID labelling is a little

concerning. I would like to see independent evidence of the SxlRac protein binding chromatin.

Do antibodies against this form (or any form) of Sxl bind chromatin in salivary gland polytene chromosomes, for example? Does Sxl from other insects where Sxl has no role in sex determination bind chromatin?

Author Response:

Regarding the reviewer's overall concerns about the legitimacy of the Sxl binding data:

i) The fold differences between Dam-Sxl-mutants and the Dam-only control are very robust (up to 9 log2 fold change (500-fold change)), which is higher than what we observe with most transcription factors using Targeted DamID.
ii) We observed that Sxl binding was completely abrogated upon knockdown of Polr3E, confirming that the signal we observe is biologically specific and not due to technical noise or background.
iii) If the concern relates to potential Sxl binding in non-neuronal tissues such as salivary glands, we would like to clarify that all DamID constructs were expressed under elav-GAL4, a pan-neuronal driver. Furthermore, dissections were performed to isolate larval brains, with salivary glands carefully removed. This ensures that chromatin profiles were derived from neuronal tissue exclusively.
iv) Salivary gland polytene chromosome staining with a Sxl antibody in a closely related species (Drosophila virilis) show binding of Sxl to chromatin in both sexes (Bopp et al., 1996).

We have included additional text in the revised Discussion section to emphasize these points, particularly highlighting the precedent from D. virilis work showing Sxl chromatin association in both sexes (Bopp et al., 1996).

Do antibodies against this form (or any form) of Sxl bind chromatin in salivary gland polytene chromosomes, for example? Does Sxl from other insects where Sxl has no role in sex determination bind chromatin?

Author Response:

Prior work in Drosophila virilis (where Sxl is also required for sex determination and Sxl-RAC is conserved) has already demonstrated Sxl-chromatin association (using a full-length Sxl antibody) in salivary glands using polytene chromosome spreads (Bopp et al., 1996). Binding is observed in both sexes and across the genome, reflecting our observations. We will incorporate this into the revised discussion to support the chromatin-binding role of Sxl across species.

There is a clear and long-overlooked precedent for Sxl's alternative, sex-independent roles, findings that have been largely overshadowed by the gene's canonical function. Our study not only validates and extends these observations but also brings much-needed attention to this understudied aspect of fundamental biology.

Bopp D, Calhoun G, Horabin JI, Samuels M, Schedl P. Sex-specific control of Sex-lethal is a conserved mechanism for sex determination in the genus Drosophila. Development. 1996 Mar;122(3):971-82. doi: 10.1242/dev.122.3.971. PMID: 8631274.

I would like to see independent evidence of the SxlRac protein binding chromatin.

Author Response:

We do not believe this is necessary:
i) Our data demonstrated that a large N-terminal truncation of Sxl (removing far more of the N-terminal region than is absent in Sxl-RAC) does not impair chromatin binding.
ii) Our deletion experiments show that it is the central domain of Sxl that is required for chromatin association (as removal of the N-or C-terminal domain has no effect). This central domain is unaffected in Sxl-RAC.
iii) Independent Y2H experiments have shown that it is exclusively the RBD-1 (RNA binding domain 1) of the central domain of Sxl that interacts with Polr3E (Dong et al., 1999). Sxl-RAC contains this region, therefore will be recruited by Polr3E.
iv) Review 3 also believes that this is not necessary (see cross-review below) and highlights the robustness of the Y2H experiments performed by Dong et al., 1999.

Also, given that their DamID experiments reveal that Sxl binds half of the genes encoded in the Drosophila genome, finding that it binds around half of the tRNA genes is perhaps not surprising.

Author Response:

Our data show that Sxl binds to a range of Pol III-transcribed loci, and this binding pattern supports the proposed model that Sxl plays a broader regulatory role in Pol III activity. Within these Pol III targets, tRNA genes represent a specific and biologically relevant subset. The emphasis on tRNAs is not to suggest they are the exclusive or primary targets of Sxl, but rather to highlight a functionally important class of Pol III-transcribed elements that align with the model we are proposing. We will revise the text to better reflect this framing and avoid any confusion regarding the scope of Sxl's binding profile.

I would like to see evidence beyond citing a 1999 yeast two-hybrid study that Sxl and Polr3E directly interact with one another.

Author response:

We do not believe this is necessary (these points were also mentioned above):

i) The Dong et al., 1999 study was highly comprehensive in its characterisation of Sxl binding to Polr3E.

ii) Our DamID data provide strong complementary evidence for this interaction: knockdown of Polr3E completely abolishes Sxl's recruitment to chromatin, strongly supporting the relevance of the interaction in vivo.

iii) Review 3 highlights the robustness of the Y2H experiments performed by Dong et al., 1999.

In my opinion, the differences in lethality observed with loss of Sxl versus control are unlikely to be meaningful given the different genetic backgrounds.
The similar defects in negative geotaxis could be meaningful, but I'm unsure how often this phenotype is observed. What other class of genes affect negative geotaxis? It's a little unclear why having reduced expression of metabolic and pigment genes or of tRNAs would improve neuronal function.

Author response:

While the differences in survival were indeed subtle, they were statistically significant and thus warranted inclusion. Our primary aim in this section was to demonstrate that knockdown of Sxl or Polr3E results in comparable behavioural and transcriptional phenotypes, suggesting overlapping functional roles. In this context, we believe the data were presented transparently and effectively support our interpretation.

Regarding the negative geotaxis phenotype, we appreciate the reviewer's interest and agree that it is both intriguing and atypical. For this reason, we performed the assay multiple times, particularly in Polr3e knockdowns, to confirm the robustness of the result. To address potential confounding variables, we carefully selected control lines that account for genetic background and transgene insertion site, including KK controls and attP40-matched lines. We also employed multiple independent RNAi lines targeting Sxl to validate the phenotype across different genetic backgrounds.

Although the observed improvement in climbing is unexpected, it is not without precedent in the RNA polymerase III field. Notably, Malik et al. (2024) demonstrated that heterozygous Polr3DEY/+ mutants exhibit a significantly delayed decline in climbing ability with age. We

allude to this in the discussion and will revise the text to emphasise this connection more explicitly.

Finally, while we recognise that negative geotaxis is a relatively broad assay and thus does not pinpoint the precise cellular mechanisms involved, we interpret the phenotype as suggesting a neural basis and a functional role for Sxl in the nervous system.

One would expect that not just the same classes of genes would be affected by loss and overexpression of Sxl, but the same genes would be affected - are the same genes changing in opposite directions in the two experiments or just the same classes of genes. Likewise, are the same genes changing expression in the same direction with both Sxl and the Polr3E loss? Also, why are tRNA genes not also affected with Sxl loss. Finally, they describe the changes in gene expression as being in male adult neurons, but the sequencing was done of entire heads - so no way of knowing which cell type is showing differential gene expression.

Author response:

While we do examine gene classes, our approach also includes pairwise correlation analyses of gene expression changes between specific genotypes. Notably, we observed a significant positive correlation between Polr3e knockdowns and Sxl knockdowns, and a significant negative correlation between Sxl-RAC–expressing flies and Sxl knockdowns. Furthermore, we examined Sxl-DamID target genes within our RNA-seq datasets and found a consistent relationship between Sxl targets and genes differentially expressed in Polr3e knockdowns.

Regarding the Pol III qPCR results, we note that tRNA expression changes may require a longer duration of RNAi induction (e.g., beyond 4 days) to become apparent, especially given that phenotypic effects such as changes in lifespan and negative geotaxis only emerge after 20 days or more. It is also plausible that Sxl knockdown leads to a partial reduction in Pol III efficiency, which may not be readily detectable through bulk Pol III qPCRs. We are willing to repeat Pol III qPCRs at later timepoints to further investigate this trend.

Finally, we infer that gene expression changes observed in our RNA-seq data are of neuronal origin, as all knockdown and overexpression constructs used in this study were driven pan-neuronally using elav-/nSyb-GAL4. While we acknowledge that bulk RNA-seq does not provide cell-type resolution, tissue-specific assumptions are widely used in the field when driven by a relevant promoter.

I'm also not sure what I'm supposed to be seeing in panel 5F (or in the related supplemental figure) and if it has any meaning - If they are using the Sxl-T2A-Gal4 to drive mCherry, I think one would expect to see expression since Sxl transcripts are made in both males and in females. Also, one would expect to see active protein expression (OPP staining) in most cells of the adult male brain and I think that is what is observed, but again, I'm not sure

what I'm supposed to be looking at given the absence of any arrows or brackets in the figures.

Author Response:

Due to the presence of the T2A tag and the premature stop codon in exon 3 of early male Sxl transcripts, GAL4 expression is not expected in males unless the head-specific Sxl-RAC isoform is produced. In males expressing this isoform, mCherry serves as a reporter for Sxl expression, allowing us to visualize which cells are producing Sxl-RAC.

To address the reviewer's question about the meaning of the OPP colocalisation data, we have now performed additional quantitative experiments (new Figure 6b-e). These show that:

1) Neuronal Sxl knockdown significantly reduces OPP incorporation (p=0.0048), indicating decreased protein synthesis
2) SxlRAC overexpression increases OPP incorporation (p=0.0128), indicating increased protein synthesis
3) Colocalisation analysis (Rcoloc=0.81) confirms that cells expressing high levels of Sxl-RAC show correspondingly high translation rates

These bidirectional effects provide direct functional evidence that Sxl levels regulate translational capacity in neurons, supporting our model linking Sxl to tRNA expression to translation.

To quantitatively assess this relationship, we performed colocalisation analysis using ImageJ, which showed a positive correlation between Sxl and OPP signal intensity, supporting this interpretation. It is also evident from our images that regions with lower levels of protein synthesis (such as the neuropil - as shown in independent studies Villalobos-Cantor et al., 2023) concurrently lack Sxl-related signal. We would be happy to further highlight regions or individual cells exhibiting relatively high or low levels of Sxl and/or OPP signal to better illustrate this relationship. We can also test the effects of knockdown/overexpression on general protein synthesis.

Villalobos-Cantor S, Barrett RM, Condon AF, Arreola-Bustos A, Rodriguez KM, Cohen MS, Martin I. Rapid cell type-specific nascent proteome labeling in Drosophila. Elife. 2023 Apr 24;12:e83545. doi: 10.7554/eLife.83545. PMID: 37092974; PMCID: PMC10125018.

Minor comments:

Line 223 - 225 - I believe that it is expected that Sxl transcripts would be broadly expressed

in the male and female adult, given that it is only the spliced form of the transcript that is female specific in expression.

Author Response:

We used RNAi lines targeting the core exons (4–7) of the functional Sxl transcripts, which are absent from the male-specific isoform responsible for sex determination due to early truncation. Our immunohistochemistry data indicate that Sxl-RAC is expressed in the male brain, specifically in neurons. Therefore, knockdown in males would selectively target this transcript variant, without affecting the sex-determining isoform.

Line 236 - 238 - Sentence doesn't make sense.

We have addressed this.

Reviewer #2 (Significance (Required)):

It would be significant to discover that a gene previously thought to function in only sex determination and dosage compensation also moonlights as a regulator of RNA polymerase III activity. Unfortunately, I am not convinced by the work presented in this study that this is the case.

My expertise is in Drosophila biology, including development, transcription, sex determination, morphogenesis, genomics, transcriptomics, DNA binding

Reviewer #3 (Evidence, reproducibility and clarity (Required)):

Storer, McClure and colleagues use genome-wide DNA-protein binding assays, transcriptomics, and genetics to work out that Drosophila Sxl, widely known as an RNA-binding protein which functions as a splicing factor to determine sex identity in Drosophila and related species, is also a chromatin factor that can stimulate transcription by Pol III and Pol II of genes involved with metabolism and protein homeostasis, specifically some encoding tRNAs.

The evidence for the tenet of the paper -- that Sxl acts as a chromatin regulator with Polr3E, activating at least some of its targets with either Pol III or Pol II -- is logical and compelling, the paper is well written and the figures well presented. Of course, more experiments could always be wished for and proposed, but I think this manuscript could be published in many journals with just a minor revision not involving additional experiments. I have a few specific comments below, all minor.

Scientific points:

- The approach taken for the evaluation of Sxl DNA-binding activity in Fig2 is not entirely clear. I assume these are crosses of elav-Gal4 x different UAS- lines, then using males or females for UAS-Sxl-Full-Length. But what about the others? Were the experiments done in males only? This is hinted at in the main text but not explicitly indicated in the figure or the methods (at least, that I could easily find). And is this approach extended to all other experiments? Longevity? Climbing assays? Considering the role of Sxl, it may be helpful to be fastidiously systematic with this.

Author Response:

We have revised the wording throughout to ensure greater clarity, and we agree that a systematic and precise approach is essential given the nature of the study. Changes include more precise language in the Abstract, clearer description of experimental approaches in Results, and focused Discussion on the core mechanistic findings.

- In the discussion, lines 360-61, the authors say: Indeed, knockdown of Polr3E leads to a loss of Sxl binding to chromatin, suggesting a cooperative mechanism. Maybe I am misunderstanding the authors, but when I read "cooperation" in this context I think of biochemical cooperative binding. This is possible, but I do not think a simple 'requirement' test can suggest specifically that this mechanistic feature of biochemical binding is at play. I would expect, for starters, a reciprocal requirement for binding (which is not tested), and some quantitative features that would be difficult to evaluate in vivo. I do not think cooperative binding needs to be invoked anyway, as the authors do not make any specific point or prediction about it. But if they do think this is going on, I think it would need to be referred to as a speculation.

Author Response:

We have revised the text to more accurately reflect a functional requirement (Polr3E is required for Sxl chromatin binding), rather than implying direct biochemical cooperation at the chromatin level.

- In lines 428-432, the authors discuss the ancestral role of Sxl and make a comparison with ELAV, in the context of an RNA-binding protein that has molecular functions beyond those of a splicing factor, considering the functions of ELAV in RNA stability and translation, and finishing with "suggesting that similar regulatory mechanisms may be at play". I do not understand this latter sentence. Which mechanisms are these? Are the authors referring to the molecular activities of ELAV and SXL? But what would be the similarity? SXL seems to

have a dual capacity to bind RNA and protein interactors, which allows it to work both in chromatin-level regulation as well as post-transcriptionally in splicing; but ELAV seems rather to take advantage of its RNA binding function to make it work in multiple RNA-related contexts, all post-transcriptional. I do not see an obvious parallel beyond the fact that RNA binding proteins can function at different levels of gene expression regulation -- but I would not say this parallel are "similar regulatory mechanisms", so I find the whole comparison a bit confusing.

Author Response:

We have streamlined this section, reducing speculative content while maintaining appropriate context about Sxl's potential ancestral functions. The Discussion now focuses primarily on the direct mechanistic findings while noting the possibility of conserved functions in other species.

- One aspect of the work that I find is missing in the discussion is the possibility that the simultaneous capacity of Sxl for RNA binding and Polr3E binding: are these mutually exclusive? if so, are they competitive or hierarchical? how would they be coordinated anyway?

Author Response:

This is an interesting point, but we believe our data already addresses this question. Our experiments with RNA-binding-deficient mutants (SxlGS and SxlRNA) demonstrate that Sxl retains full chromatin-binding capacity via Polr3E even when unable to bind RNA (Fig. 2). This shows that RNA binding and Polr3E binding are not mutually exclusive and do not appear to be competitive. The two activities can be mechanistically separated, suggesting they involve distinct molecular interfaces within the RBD region. Therefore, coordination between these functions is not required for chromatin recruitment, though both activities may contribute to Sxl's overall biological functions in different contexts.

- The only aspect of the paper where I found that one could make an experimental improvement is the claim that Sxl induces the expression of genes that have the overall effect of stimulating protein synthesis. The OPP experiment shows a correlation between the expression of Sxl and the rate of protein synthesis initiation. However, a more powerful experiment would be, rather obviously, to introduce Sxl knock-down in the same experiment, and observe whether in Sxl-expressing neurons the incorporation of OPP is reduced. I put this forth as a minor point because the tenet of the paper would not be affected by the results (though the perception of importance of the newly described function could be reinforced).

Author Response:

We thank the reviewer for this experimental suggestion. We have now completed these experiments and they substantially strengthen the manuscript. The new data (Figure 6b-e) demonstrate that Sxl bidirectionally regulates protein synthesis in adult male neurons:

- Sxl knockdown significantly reduces OPP incorporation (p=0.0048; Fig. 6c), demonstrating that loss of Sxl decreases translational output in neurons

- SxlRAC overexpression significantly increases OPP incorporation (p=0.0128; Fig. 6e), showing the reciprocal effect

- Co-localisation analysis confirms that regions with high Sxl expression exhibit elevated nascent protein synthesis (Rcoloc=0.81; Fig. 6a, Fig. S6)

These bidirectional effects move beyond correlation to provide direct functional evidence that Sxl levels regulate translational capacity in neurons. Combined with our tRNA expression data (showing reciprocal changes in pre-tRNA levels with Sxl manipulation; Fig. 5, Fig. S5), these results support our model that Sxl influences translation through Polr3E-dependent regulation of tRNA synthesis.

- In a similar way, it would be interesting to know whether the recruitment of Polr3E and Sxl to chromatin is co-dependent or Sxl follows Polr3E. This is also a minor point because this would possibly refine the mechanism of recruitment but does not alter the main discovery.

Author Response:

We have addressed a similar point for Reviewer 2 (see below) and included a Discussion point for this:

If Sxl were required for Polr3E recruitment to RNA Pol III, then, in most male Drosophila melanogaster cells, Polr3E would not be incorporated, and males would not be viable (as it is essential for Pol III activity). While it is possible that there could be a subtle effect on Polr3E recruitment, such an experiment would not alter the central conclusion of our study - that Sxl is recruited to chromatin (accessory to the Pol III complex) via Polr3E.

Figures and reporting:

- In Figure 2, it would be helpful to see the truncation coordinate for the N and C truncations.

We have added the truncation coordinates to the Figure 2 legend: N-terminal deletion removes amino acids 1-120; C-terminal deletion removes amino acids 281-354.

- In Figure 3D, genomic coordinates are missing.

We have added genomic coordinates to Figure 3D (chr2L, 30 kb scale bar shown).

- In Figure 3E, the magnitude in the Y axis is not entirely clear (at least not to me). How is the amount of binding across the genome quantified? Is this the average amplitude of normalised TaDa signal across the genome? Or only within binding intervals?

We have clarified the Figure 3E legend to explain that the Y-axis shows the log_2 fold change of Dam-Sxl binding over Dam-alone control, calculated as the genome-wide average of normalized TaDa signal across all GATC fragments. This represents binding across the entire genome, not just within called peaks, providing an unbiased measure of overall chromatin occupancy.

- Figure S3E-F: it would be interesting to show the degree of overlap between the downregulated genes that are also binding targets (regardless of the outcome).

We have added this analysis to Figure S3e-f. The data show that 65% of Dam-Sxl chromatin targets are significantly downregulated in Sxl knockdowns (Fig. S3e), and 69% of Dam-Sxl targets are downregulated in Polr3E knockdowns (Fig. S3f), demonstrating substantial overlap between chromatin binding and transcriptional regulation.

- Figure 5C-E: similarly to Figure S3, it would be interesting to know how the transcriptional effects compare with the binding targets.

As noted above, we have included this comparative analysis showing the overlap between Dam-Sxl binding targets and transcriptional changes. The high percentage of bound targets showing transcriptional changes (65-69%) supports a direct regulatory relationship between Sxl chromatin occupancy and gene expression.

- Authors use Gehan-Breslow-Wilcoxon to test survival, which is a bit unusual, as it gives more weight to the early deaths (which are rare in most Drosophila longevity experiments). Is there any rationale behind this? It may be even favour their null hypothesis.

We used the Gehan-Breslow-Wilcoxon test because it provides greater sensitivity to differences occurring across the entire survival curve, including mid-to-late timepoints where the effects of neuronal Sxl manipulation are most apparent. While this test does weight early events more heavily, we also performed Log-rank (Mantel-Cox) tests as an alternative analysis. Both tests gave concordant results for all survival experiments, confirming the robustness of the observed phenotypes. We have added a note in the Methods clarifying that both statistical approaches were applied and yielded consistent conclusions.

Writing and language:

- Introduction finishes without providing an outline of the findings (which is fine by me if that is what the authors wanted).

We opted to end the Introduction by establishing the biological context and open questions rather than providing an explicit outline of findings. The Introduction concludes by highlighting the evidence for Tra-independent Sxl functions (TIF phenomenon) and noting that "Collectively, these findings support the existence of an alternative, yet unidentified, mode of Sxl action beyond its canonical role in sex-specific splicing." This sets up the rationale for our study without previewing the specific results, which we felt maintained better narrative flow for the reader.

- In lines 361-5, the authors say "We speculate that this interaction not only facilitates Pol III transcription but may also influence chromatin architecture and RNA Pol II-driven transcription as observed with Pol III regulation in other organisms". "This interaction" refers to Polr3E-Sxl-DNA interaction and with "Pol III transcription" I presume the authors refer to transcription executed by Pol III. I am not clear about the meaning of the end of the sentence "as observed with Pol III regulation in other organisms". What is the observation, exactly? That Pol III modifies chromatin in Pol II regulated loci, or that Pol III interactors change chromatin architecture?

We have clarified this sentence in the revised Discussion. The observation refers to studies in other organisms showing that Pol III components can influence chromatin architecture at Pol II-transcribed genes. Specifically: Carrière et al. (2012) showed genomic binding of Pol III machinery at Pol II loci in mouse ES cells; Wang et al. (2014) demonstrated that compromised Pol III assembly alters intergenic Pol II transcription in yeast; and Jiang et al. (2022) showed that Pol III maintains local chromatin architecture affecting mRNA transcription. We have revised the sentence to read: "We speculate that this interaction not only facilitates Pol III transcription but may also influence chromatin architecture and RNA Pol II-driven transcription, as Pol III components have been shown to regulate chromatin structure and Pol II activity in other organisms (Carrière et al., 2012; Wang et al., 2014; Jiang et al., 2022)."

- DPE abbreviation is not introduced (and only used once).

We have removed the DPE abbreviation and now write out "days post-eclosion" in full throughout the manuscript for clarity.

- A few typos:
Line 41 ...splicing of the Sxl[late] transcripts, which is [ARE?] constitutively transcribed (Keyes et al.,...
Line 76 ...sexes but appears restricted to the nervous system [OF] male pupae and adults (Cline et

Line 289 ...and S41). To assess any effect [ON]translational output, O-propargyl-puromycin (OPP)o
Line 323 ...illustrating that the majority (72%) changes in tRNA levels [ARE] due to upregulation...hi
Line 402 ...it was discovered [WE DISCOVERED]
Line 792 ...Sxl across chromosomes X, 2 L/R, 3 L/R and 4. The y-axis represents the log[SYMBOL] ratio... This happens in other figure legends as well.

We thank the reviewer for the careful reading. We have corrected all the identified typos.

Referee Cross-commenting

Reviewer 1 asks how physiological is the Sxl chromatin-association assay. I think the loss of association in Polr3E knock-down and the lack of association of other splicing factors goes a long way into answering this question. It is true that having positive binding data specifically for Sxl-RAC and negative binding data for a deletion mutant of the RMM domain would provide more robust conclusions (see below), but I am not sure it is completely necessary -- though this will depend on which journal the authors want to send the paper to.

I think that the comment of reviewer 1 about the levels of expression of Sxl-DAM does not apply here because of the way TaDa works - it relies on codon slippage to produce minimal amounts of the DAM fusion protein, so by construction it will be expressed at much lower levels than the endogenous protein.

Reviewer 1 also asks whether Polr3E chromatin-association is also dependent on Sxl, to round up the model and also as a way to address whether Sxl association to chromatin is real. While I agree with this on the former aim (this would be a nice-to-have), I think I disagree on the latter; there is no need for Polr3E recruitment to depend on Sxl for Sxl association to chromatin to be physiologically relevant. Polr3E is a peripheral component of Pol III and unlikely to depend on a factor of restricted expression like Sxl to interact with chromatin. The recruitment of Sxl could well be entirely 'hierarchical' and subject to Polr3E.

Revewer 2 is concerned with the fact that every mutant form of Sxl shows the same result from the DamID labelling. I have to agree with this to a point. A deletion mutant of RMM domains would address this. Microscopy evidence in salivary glands would be nice, certainly, but the system may not lend itself to this particular interaction, which might be short-lived and/or weak. I do not immediately see the relevance of the chromatin binding capacity of non-Drosophilidae Sxl -- though it might indicate that the impact of the discovery is less likely to go beyond this group.

Reviewer 2 does not find surprising that some tRNA genes (less than half) are regulated by Sxl. I think the value of that observation is just qualitative, as tRNAs are Pol III-produced transcripts, but their point is correct. A hypergeometric test could settle this.

Reviewer 2 is concerned that the evidence of direct interaction between Sxl and Polr3E is a single 1999 two-hybrid study. But that paper contains also GST pull-downs that narrow down the specific domains that mediate binding, and perform the binding in competitive salt conditions. I think it is enough. The author team, I think, are not biochemists, so finding the right collaborators and performing these experiments would take time that I am not sure is warranted.

Reviewer 2 is also concerned that the longevity assays may not be meaningful due to the difference in genetic backgrounds. This is a very reasonable concern (which I would extend to the climbing assays - any quantitative phenotype is sensitive to genetic background). However, I think the authors here may have already designed the experiment with this in mind - the controls express untargeted RNAi constructs, but I lose track of which one is control of which. This should be clarified in Methods.

Other comments are in line, I think, with what I have pointed out and I generally agree with everything else that has been said.

Reviewer #3 (Significance (Required)):

Drosophila Sxl is widely known as an RNA-binding protein which functions as a splicing factor to determine sex identity in Drosophila and related species. It is a favourite example of how splicing factors and alternative can have profound influence in biology and used cleverly in the molecular circuitry of the cell to enact elegant regulatory decisions.

In this work, Storer, McClure and colleagues use genome-wide DNA-protein binding assays, transcriptomics, and genetics to work out that Sxl is also a chromatin factor with an sex-independent, neuron-specific role in stimulating transcription by Pol III and Pol II, of genes involved with metabolism and protein homeostasis, including some encoding tRNAs.

This opens a large number of interesting biological questions that range from biochemistry, gene regulation or neurobiology to evolution. How is the simultaneous capacity of binding RNA and chromatin (with the same protein domain, RRM) regulated/coordinated? How did this dual activity evolve and which one is the ancestral one? How many other RRM-containin RNA-binding proteins can also bind chromatin? How is Sxl recruited to chromatin to both Pol II and Pol III targets and are they functionally related? If so, how is the coordination of cellular functions activated through different RNA polymerases taking place and what is the role of Sxl in this? What are the functional consequences to neuronal biology? Does this affect similarly all Sxl-expressing neurons?

The evidence for the central tenet of the paper -- that Sxl acts as a chromatin regulator with Polr3E, activating at least some of its targets with either Pol III or Pol II -- is logical and compelling, the paper is well written and the figures well presented. Of course, more

experiments could always be wished for and proposed, but I think this manuscript could be published in many journals with just a minor revision not involving additional experiments.

Reviewer #4 (Evidence, reproducibility and clarity (Required)):

The convincing analysis demonstrates a role for the Drosophila Sex determining gene sex lethal in controlling aspects of transcription in the nervous system independent of its role in splicing. Interaction with an RNA Pol III subunit mediating Sxl association with chromatin and similar knockdown phenotypes strongly support the role of Sxl in the regulation of neuronal metabolism. Given that Sxl is an evolutionary recent acquisition for sex determination, the study may reveal an ancestral role for Sxl.

The conclusions are well justified by the datasets presented and I have no issues with the study or the interpretation. Throughout the work is well referenced, though perhaps the authors might take a look at Zhang et al (2014) (PMID: 24271947) for an interesting evolutionary perspective for the discussion.

Thank you for the thoughtful suggestion. We have incorporated the findings from Zhang et al. into the Discussion.

I have some minor comments for clarification:

There is no Figure 2b, should be labelled 2 or label TaDa plots as 2b

We have labeled the TaDa genome browser tracks in Figure 2 as panel "a" to clarify the figure structure.

Clarify if Fig 2 data are larval or adult

We have clarified in the Figure 2 legend that these data are from larval brains: "Targeted DamID data from larval neurons showing chromatin occupancy..."

Fig 3d - are these replicates or female and male?

We have clarified in the Figure 3d legend that these are biological replicates: "Three biological replicates are shown for wild-type and Polr3E RNAi conditions (one female-only sample and two mixed-sex samples for wild-type; three mixed-sex samples for Polr3E RNAi)."

Please elaborate on tub-GAL80[ts] developmental defects

We have elaborated in the Results section (lines ~225) and Methods that tub-GAL80ts is used to restrict GAL4 activity to adults, thereby "bypassing developmental effects" that could arise

from neuronal Sxl manipulation during development. Flies are reared at 18°C (permissive temperature where GAL80 is active and represses GAL4) and shifted to 29°C post-eclosion to induce adult-specific knockdown or overexpression.

Fig 4e, are transcriptomics done with the VDRC RNAi line? The VDRC and BDSC RNAi lines exhibit different behaviours - former has "better" survival and Better negative geotaxis, the latter seems to have poorer survival but little geotaxis effect?

Yes, the transcriptomics in Figure 4e were performed using the VDRC #109221 line. We have clarified this in the figure legend. We acknowledge that the two RNAi lines show somewhat different behavioral phenotypes (Figure 4 vs. Figure S3a-b), which likely reflects differences in knockdown efficiency or off-target effects. However, both lines show the key survival deficit, supporting the robustness of this phenotype.

Fig S3 - volcano plot for Polr3E?

We have added a volcano plot showing differential gene expression for Polr3E knockdown (new Figure S3d), complementing the existing Sxl knockdown volcano plot (Figure S3c).

Fig S4a - legend says downregulated genes?

The Figure S4a legend correctly states "upregulated genes" - this was not an error in the manuscript. The GO analysis shows genes upregulated upon SxlRAC overexpression, consistent with increased metabolic activity.

The discussion should at least touch on the fact that Sxl amorphs (i.e. Sxl[fP7B0] are male viable and fertile, emphasising that the newly uncovered role is not essential.

We address this important point in the Discussion (lines ~388-394), noting that "While we observe a mild survival deficit in Sxl knockdowns, overexpression of SxlRAC in adults results in a marked decline in survival... These data underscore the importance of tightly regulating Sxl expression to maintain neuronal homeostasis." We further emphasize that the lifespan effects are "mild" and that the role is "dosage-sensitive" rather than essential, consistent with the viability of Sxl null males.

Reviewer #4 (Significance (Required)):

A nonessential role for Sxl in the nervous system independent of sex-determination contributes to better understanding a) the evolution of sex determining mechanisms, b) the role of RNA PolIII in neuronal homeostasis and c) more widely to the neuronal aging field. I think this well-focused study reveals a hitherto unsuspected role for Sxl.
