# Supplementary material for: Neuronal protein Sex-lethal modulates tRNA synthesis via the polymerase III subunit Polr3E in male Drosophila neurons
Source: PLoS Biol. 2026 Jul 17;24(7):e3003863. doi: 10.1371/journal.pbio.3003863 (PMC13432123; doi:10.1371/journal.pbio.3003863)
Supplement: Attachment — Submitted filename: Responses to Reviewer Comments R2_1.docx [file pbio.3003863.s066.docx]

Rev. 1:
In this manuscript, the authors show that Sxl (unlike the other splicing factors tested) binds to chromatin, mainly near the transcription start sites for both PolII and PolII transcripts. They further show that domains of Sxl required for RNA binding and other aspects of Sxl splicing function are not required for chromatin binding. They show that Sxl binding overlaps with that of Polr3E and is dependent on Polr3E (based on RNAi experiments knocking down Polr3E). Sxl bound about half of the genes in the genome (53% occupancy on expressed genes), including 156 of the 297 tRNAs encoded in Drosophila, whereas Polr3E bound 238 of the 297 tRNA genes. The authors show that knockdown of Sxl in males results in very slight decreases in long term male survival, whereas knockdown of Polr3E had more significant decreases. They show that loss of both genes improves a behavior - negative geotaxis - in the same direction. They show that there is some correlation between gene expression
changes in Sxl knock down and knockdown of Polr3E. They show that 50 of the 365 genes affected by Sxl knock down are shared with knockdown of Polr3E (although they do not indicate if the 50 genes change expression in the same direction). They show that overexpression of the form of Sxl normally expressed in male neurons leads to male lethality as well as changes in expression of 31 of the 287 tRNA genes encoded in Drosophila (a class of genes known to be PolIII dependent). They show that 23 of the 31 tRNA genes go up (only 20 are shown in the table) and nine go down. Most of the affected tRNAs are for Lysine (17; one each for Cys, Arg and Tyr, no information is shown for the three not in the table). They show that localization of a tagged version of Sxl is visible in male brains and correlates with the presence of OPP (a measure of translation levels) (Rcoloc = 0.81). Finally, they show that translation (as measured by OPP intensity) goes up when the chromatin binding form
of Sxl is overexpressed and translation goes down when Sxl is knocked down by RNAi. Concerningly, the controls in the two different experiments changed as much as the experimentals did in the individual experiments.

Many of my original concerns remain regarding the significance of the findings presented in this paper. The major ones are listed below.

The authors never share whether the same genes change expression in expected ways across experiments. They show only broad correlations. It would be nice to have shown a table of all the genes that significantly change and what direction they change in all experimental conditions.

Author Response: We have revised Figure 4 to bring the correlation analysis to the forefront, highlighting the relationships between significant datasets. In addition, we have updated the supplementary tables to include gene names corresponding to the RNA-seq and correlation analyses.

Why are Polr3E and Sxl binding to PolII transcribed genes as well as to PolIII transcribed genes? Is it possible that these proteins are simply accumulating on open chromatin? How does this binding compare with active chromatin markers?

Author Response: This is unlikely to reflect non-specific accumulation at open chromatin, as the DamID analysis pipeline includes normalisation to Dam-only controls, which account for open chromatin signal and ensure that the observed binding is specific.

It's not clear in Figure 4G if the correlation in genes that change in Sxl and PolrE3 knockdown are comparisons of genes that only go down or if they are genes that go in both directions and if the direction of expression change was accounted for in the analysis. What is plotted on the X axis?

Author Response: We have expanded the Fig. 4g legend to clarify the correlation shown in this panel. The scatter plot compares significant gene expression changes between Polr3E and Sxl knockdown samples, with each point representing a gene locus plotted by log₂ fold change in Sxl knockdown (x-axis) versus Polr3E knockdown (y-axis). Dashed lines at zero have been added to delineate quadrants of concordant and discordant expression changes (up- and downregulation).

Likewise, with the 50 genes whose expression is altered with loss of Sxl and PolrE3 shown in the Venn diagram in Figure 4G, it is unclear if the direction of change is the same in the two different knockdown backgrounds.

Author Response: As the overlap is limited, we included both up- and downregulated genes. The correlation analysis further demonstrates concordance in the direction of change between datasets.

The OPP assays to measure levels of translation are also concerning, since the levels of OPP staining change as much between the two different control samples as they change between the control and the experimental in each individual experiment. I think this may indicate that there can be considerable variation from animal to animal, experiment to experiment with this methodology. Importantly, we are never told how many animals were imaged in these experiments nor how many sections were analyzed. Even if I had more confidence in the assay, it is hard to understand why translation is changing in both directions. Translation might be expected to go down if most of the tRNAs for lysine are reduced in expression, but I wouldn't expect translation to go up with so few tRNAs changing expression - translation would require most tRNAs be upregulated.

Author Response: Given the inherent variability of immunostaining, we implemented controlled experimental conditions to minimise technical differences. All comparisons were performed and imaged in parallel using identical microscope settings, and an additional normalisation step was applied to account for inter-experimental variability. Both raw and normalised datasets are included in the supplementary information, and the within-experiment trends remain consistent. We have also included the number of animals per experiment.

In response to the concern regarding Sxl^RAC^ OPP staining, we have expanded the dataset with additional images. Although a mild increase in signal persists, it is no longer statistically significant. We thank the reviewer for highlighting this point.

Minor question:
What are the gray boxes in the top diagram in Figure 3A, 3D?

Author Response: The grey boxes in Fig. 3A and D denote individual gene regions.


Rev. 2:
In this revised manuscript, Storer et al. have incorporated additional evidence addressing one of my concerns (whether protein translation was indeed affected in SxlRAC-expressing neurons by SxlRAC) as well as the wider molecular model proposed (the effect of recruiting Sxl by Pol3Er in regulating tRNAs.

My previous stance is the same: I think the tenet of the paper, namely, that Sxl molecular function goes beyond its role as an RNA binding protein to regulate splicing and dose compensation but as a chromatin binding protein recruited by Pol3Er to regulate transcription, is novel and sufficiently backed by the data to be of interest as a paper without adding more. With the new data (the imaging data, at least), the original claim seems even more robust.

Therefore, my recommendation is the same: publication with minor modification/clarification.

My minor comments to this version of the paper are as follows:

1 - I am not very enthusiastic about the new emphasis on Sxl exerting its function through increasing tRNA expression. This is an expansion of the original claim that feels a bit overstretched. There are two things that jar in this model.

+ 1A - One, that there is no evidence that the effects of Sxl happen through changes in tRNA abundance. It is a reasonable speculation, but just that. To address this, it would be necessary to do some epistatic analysis, i.e. over expressing tRNA-Leu-TAT or a tRNA-Lys (or a combination) and observe a rescue of the phenotypes observed with SxlRNAi (lifespan, climbing). That would be a difficult experiment to run satisfactorily because of the redundancy and complexity of tRNA loci. A simpler one would be to show that you can at least can phenocopy the SxlRNAi with tRNA knock-down, but I think it would still be technically challenging for the same reason. I do not mind these experiments not being done, but then this emphasis and some of the statements may not be warranted (i.e. the epigraph title in line 327 "Sxl modulates neuronal translation downstream of tRNA regulation" - without epistasis analysis the claim that tRNA is downstream cannot be made - or tRNAs being mentioned
in the title and Lys-tRNA in the abstract).

+ 1B - Two, the observations about regulation of tRNA expression reported in the paper. There are statistically significant effects on multiple tRNAs, most repeatedly Lysine-bearing tRNAs, however:

(1Bi) The effects seem weak: as most log2FC values are below 0.7, which means increases of expression of 60% at most; yes, for some amino acids there might be a compound effect of having multiple tRNAs experiencing this but, is this biochemically enough to expect an increase of ribosomal activity/efficiency? Is there literature that justifies this inference? If not, it is only a reasonable speculation;

(1Bii) Also, Lysine is the amino acid with most tRNAs unregulated but the pre-tRNA qPCR assays are done for a His, Leu and Ile tRNAs, and only one of them (Ile) is statistically significant (unless I have missed something). So the picture of Sxl increasing tRNAs to increase protein synthesis rates seems mechanistically a bit muddled (though the OPP assays seem convincing at face vale, see below). Statements like in lines 295-97 make this a bit confusing: "... a striking enrichment for Lys-tRNAs precursor tRNA levels, in particular pre-tRNATAT, were elevated relative to the Pol II-specific U3 transcript, as measured by qPCR (Fig. S6), with reversed trends in Sxl knockdowns (Fig. S6)." pre-tRNATAT is an isoleucine tRNA !

Author Response: We agree with these comments and have revised the text to more accurately reflect the results. The qPCR experiments were intended to confirm the direction of change in tRNA levels (Pol III output) following Sxl manipulations, using established methods and primer designs. However, we consider the small RNA-seq dataset to provide a more comprehensive and quantitative assessment of Sxl^RAC^ function, and it is therefore prioritised in the main figure.

While the qPCR results support a directional shift, they are more limited, as expression is normalised to U3 alone. Accordingly, we have moved these data to the supplementary information and now refer to them more cautiously in the text. We hope this revision clarifies the interpretation and improves the overall model.

Changes in tRNA abundance are typically modest in magnitude. Previous studies have shown that regulation often occurs at the level of specific tRNA iso-acceptors rather than global tRNA pools, with relatively small but coordinated changes that are nonetheless functionally significant (PMC11398488). Our observations are consistent with this established behaviour.

Finally, we have combined the tRNA and protein synthesis data into a single figure to facilitate a more integrated and exploratory interpretation of the results. This presentation is intended to encourage further investigation into the role of Sxl, rather than prematurely constraining its functional interpretation.

2 - I appreciate the effort of the authors in performing the imaging experiments I suggested (OPP incorporation in Sxl RNAi and SxlRAC over-expression and co-localisation analysis). However, the methods are not sufficiently clear about how these were performed. In particular:

+ 2A - Co-localisation analysis needs a baseline control of random co-localisation - absolute measures are not evidence enough. I suggest the authors establish a comparison between OPP and DAPI, for instance; that should give a much lower co-localisation factor value. Also, while the authors describe the pipeline of analysis, they do not explain what "enhanced contrast" means in the tables where they provide the data values. In principle, there should be no contrast enhancement or if there is, it should be done with a reproducible algorithm whose details are included in Methods.

Author Response: We thank the reviewer for their helpful suggestions, which prompted us to revise our colocalisation analysis approach. In response, we have removed the “enhanced contrast” step from the pipeline and instead implemented a “background removal” method, which provides improved coverage of the central brain region. In addition, we now include analyses of both raw (unprocessed) images and a baseline comparison in the Supplementary Data to support transparency and robustness of the results. We have also revised the Methods section to provide a more detailed description of the analytical workflow.

+ 2B - Intensity comparisons with histofluorescence need to be carefully explained to be accepted at face value. The authors do not provide information about image acquisition metadata (i.e. laser intensities, pixel dwell, gain, laser warm-up time, etc) nor intra-image and inter-image normalisation. For instance, the data tables have a normalised intensity column but this normalisation procedure is not described, and it should.

Author Response: We have revised the Methods section to include a more detailed description of the analytical workflow, including clarification of the normalised intensity calculation. In addition, we have added a Supplementary Table providing the image acquisition metadata.

We thank the reviewers for their careful reading of our manuscript and for their constructive suggestions, which have helped improve the clarity and quality of the work. We believe the revisions have strengthened the manuscript and made the presentation more accurate and engaging.
