# Supplementary material for: Neuronal protein Sex-lethal modulates tRNA synthesis via the polymerase III subunit Polr3E in male Drosophila neurons
Source: PLoS Biol. 2026 Jul 17;24(7):e3003863. doi: 10.1371/journal.pbio.3003863 (PMC13432123; doi:10.1371/journal.pbio.3003863)
Supplement: Peer review history

# Author comment: Author response to Decision Letter 0

**DOI:** 10.1371/journal.pbio.3003863.r001

27 Jul 2025

Attachment Submitted filename: 157578_1_rebuttal_3448522_szjvj8.pdf

# Editor report: Decision Letter 0

**DOI:** 10.1371/journal.pbio.3003863.r002

**Authors:** Ines Alvarez-Garcia

6 Aug 2025

Dear Dr Southall,

Thank you for submitting via Review Commons your manuscript entitled "Sex-lethal is recruited to chromatin by Polr3E to regulate neuronal tRNA synthesis in males" for consideration as a Research Article by PLOS Biology.

Your manuscript has now been evaluated by the PLOS Biology editorial staff as well as by an academic editor with relevant expertise and I am writing to let you know that we would like to consider a revision of the manuscript.

However, before we can send you the decision, we need you to complete your submission by providing the metadata that is required for full assessment. To this end, please login to Editorial Manager where you will find the paper in the 'Submissions Needing Revisions' folder on your homepage. Please click 'Revise Submission' from the Action Links and complete all additional questions in the submission questionnaire.

Once your full submission is complete, your paper will undergo a series of checks. After your manuscript has passed the checks, I will send you the decision. To provide the metadata for your submission, please Login to Editorial Manager (https://www.editorialmanager.com/pbiology) within two working days, i.e. by Aug 08 2025 11:59PM.

During the process of completing your manuscript submission, you will be invited to opt-in to posting your pre-review manuscript as a bioRxiv preprint. Visit http://journals.plos.org/plosbiology/s/preprints for full details. If you consent to posting your current manuscript as a preprint, please upload a single Preprint PDF.

Feel free to email us at plosbiology@plos.org if you have any queries relating to your submission.

Kind regards,

Ines

--

Ines Alvarez-Garcia, PhD

Senior Editor

PLOS Biology

ialvarez-garcia@plos.org

# Author comment: Author response to Decision Letter 1

**DOI:** 10.1371/journal.pbio.3003863.r003

7 Aug 2025

Attachment Submitted filename: 157578_1_rebuttal_3448522_szjvj8_auresp_1.pdf

# Editor report: Decision Letter 1

**DOI:** 10.1371/journal.pbio.3003863.r004

**Authors:** Ines Alvarez-Garcia

12 Aug 2025

Dear Dr Southall,

Thank you for sending us via Review Commons your manuscript entitled "Sex-lethal is recruited to chromatin by Polr3E to regulate neuronal tRNA synthesis in males" for consideration at PLOS Biology. Your manuscript and revision plan in response to the Review Commons' reports have been evaluated by the PLOS Biology editors and an Academic Editor with relevant expertise.

In light of the discussions, we would like to invite you to revise the work to thoroughly address the reviewers' reports according to your revision plan, but also including the experiments requested by Reviewer 3 - tRNA qPCR at later timepoint in Sxl KD flies and OPP staining in Sxl KD / Sxl-RAC flies - as we do think they will complement the study.

Given the extent of revision needed, we cannot make a decision about publication until we have seen the revised manuscript and your response to the reviewers' comments. Your revised manuscript is likely to be sent for further evaluation by all or a subset of the reviewers.

In addition to these revisions, you will need to complete some formatting changes, which you will receive in a follow up email. A member of our team will be in touch with a set of requests shortly.

We expect to receive your revised manuscript within 3 months. Please email us (plosbiology@plos.org) if you have any questions or concerns, or would like to request an extension.

At this stage, your manuscript remains formally under active consideration at our journal; please notify us by email if you do not intend to submit a revision so that we may withdraw it.

**IMPORTANT - SUBMITTING YOUR REVISION**

Your revisions should address the specific points made by each reviewer. Please submit the following files along with your revised manuscript:

1. A 'Response to Reviewers' file - this should detail your responses to the editorial requests, present a point-by-point response to all of the reviewers' comments, and indicate the changes made to the manuscript.

*NOTE: In your point-by-point response to the reviewers, please provide the full context of each review. Do not selectively quote paragraphs or sentences to reply to. The entire set of reviewer comments should be present in full and each specific point should be responded to individually, point by point.

You should also cite any additional relevant literature that has been published since the original submission and mention any additional citations in your response.

2. In addition to a clean copy of the manuscript, please also upload a 'track-changes' version of your manuscript that specifies the edits made. This should be uploaded as a "Revised Article with Changes Highlighted" file type.

3. Resubmission Checklist

When you are ready to resubmit your revised manuscript, please refer to this resubmission checklist: https://plos.io/Biology_Checklist

To submit a revised version of your manuscript, please go to https://www.editorialmanager.com/pbiology/ and log in as an Author. Click the link labelled 'Submissions Needing Revision' where you will find your submission record.

Please make sure to read the following important policies and guidelines while preparing your revision and fulfil the editorial requests:

a) *PLOS Data Policy*

Please note that as a condition of publication PLOS' data policy (http://journals.plos.org/plosbiology/s/data-availability) requires that you make available all data used to draw the conclusions arrived at in your manuscript. If you have not already done so, you must include any data used in your manuscript either in appropriate repositories, within the body of the manuscript, or as supporting information (N.B. this includes any numerical values that were used to generate graphs, histograms etc.). Please also indicate in each figure legend where the data can be found. For an example see here: http://www.plosbiology.org/article/info%3Adoi%2F10.1371%2Fjournal.pbio.1001908#s5

b) *Published Peer Review*

Please note while forming your response, if your article is accepted, you may have the opportunity to make the peer review history publicly available. The record will include editor decision letters (with reviews) and your responses to reviewer comments. If eligible, we will contact you to opt in or out. Please see here for more details:

https://blogs.plos.org/plos/2019/05/plos-journals-now-open-for-published-peer-review/

c) *Blot and Gel Data Policy*

Please provide the original, uncropped and minimally adjusted images supporting all blot and gel results reported in an article's figures or Supporting Information files. We will require these files before a manuscript can be accepted so please prepare them now, if you have not already uploaded them. Please carefully read our guidelines for how to prepare and upload this data: https://journals.plos.org/plosbiology/s/figures#loc-blot-and-gel-reporting-requirements

d) *Protocols deposition*

To enhance the reproducibility of your results, we recommend that if applicable you deposit your laboratory protocols in protocols.io, where a protocol can be assigned its own identifier (DOI) such that it can be cited independently in the future. For instructions see: https://journals.plos.org/plosbiology/s/submission-guidelines#loc-materials-and-methods

Thank you again for your submission to our journal. We hope that our editorial process has been constructive thus far, and we welcome your feedback at any time. Please don't hesitate to contact us if you have any questions or comments.

Sincerely,

Ines

--

Ines Alvarez-Garcia, PhD

Senior Editor

PLOS Biology

ialvarez-garcia@plos.org

# Author comment: Author response to Decision Letter 2

**DOI:** 10.1371/journal.pbio.3003863.r005

5 Feb 2026

Attachment Submitted filename: Storer et al - Reponse to Reviewers.pdf

# Peer review: Decision Letter 2

**DOI:** 10.1371/journal.pbio.3003863.r006

**Authors:** Ines Alvarez-Garcia

30 Mar 2026

Dear Dr Southall,

Thank you for your patience while we considered your revised manuscript entitled "Sex-lethal is recruited to chromatin by Polr3E to regulate neuronal tRNA synthesis" for consideration as a Research Article at PLOS Biology. Your revised study has now been evaluated by the PLOS Biology editors, the Academic Editor and two of the original reviewers.

The reviews are attached below. As you will see, the reviewers find the paper very much improved, but they raise several remaining points that would need to be addressed before we can consider the manuscript for publication. Reviewer 1 notes that it is not mentioned if the same genes change expression in expected ways across experiments, thus you should provide a table with all the gene information. The reviewer also raises concerns about the OPP assays performed to measure levels of translation because the levels of OPP staining change in a similar way among the two different controls than in the experimental animals, suggesting a high degree of variation among individuals. Please provide an explanation and the experimental details, which seem to be missing. Reviewer 2 is not convinced about the conclusion that Sxl exerts its function via increased tRNA expression, thus you should either provide more evidence to support this finding or tone down the statement. In addition, this reviewer thinks you should improve the colocalization analysis to complete the new imaging experiments.

In light of the reviews, which you will find at the end of this email, we are pleased to offer you the opportunity to address the remaining points from the reviewers in a revision that we anticipate should not take you very long. We will then assess your revised manuscript and your response to the reviewers' comments with our Academic Editor aiming to avoid further rounds of peer-review, although we might need to consult with the reviewers, depending on the nature of the revisions.

In addition to these revisions, you may need to complete some formatting changes, which you will receive in a follow up email. A member of our team will be in touch with a set of requests shortly. If you do not receive a separate email within a few days, please assume that checks have been completed, and no additional changes are required.

We expect to receive your revised manuscript within 1 month. Please email us (plosbiology@plos.org) if you have any questions or concerns, or would like to request an extension.

At this stage, your manuscript remains formally under active consideration at our journal; please notify us by email if you do not intend to submit a revision so that we withdraw the manuscript.

**IMPORTANT - SUBMITTING YOUR REVISION**

Your revisions should address the specific points made by each reviewer. Please submit the following files along with your revised manuscript:

1. A 'Response to Reviewers' file - this should detail your responses to the editorial requests, present a point-by-point response to all of the reviewers' comments, and indicate the changes made to the manuscript.

*NOTE: In your point-by-point response to the reviewers, please provide the full context of each review. Do not selectively quote paragraphs or sentences to reply to. The entire set of reviewer comments should be present in full and each specific point should be responded to individually.

You should also cite any additional relevant literature that has been published since the original submission and mention any additional citations in your response.

2. In addition to a clean copy of the manuscript, please also upload a 'track-changes' version of your manuscript that specifies the edits made. This should be uploaded as a "Revised Article with Changes Highlighted " file type.

3. Resubmission Checklist

When you are ready to resubmit your revised manuscript, please refer to this resubmission checklist: https://plos.io/Biology_Checklist

To submit a revised version of your manuscript, please go to https://www.editorialmanager.com/pbiology/ and log in as an Author. Click the link labelled 'Submissions Needing Revision' where you will find your submission record.

Please make sure to read the following important policies and guidelines while preparing your revision and fulfil the editorial requests:

a) *PLOS Data Policy*

Please note that as a condition of publication PLOS' data policy (http://journals.plos.org/plosbiology/s/data-availability) requires that you make available all data used to draw the conclusions arrived at in your manuscript. If you have not already done so, you must include any data used in your manuscript either in appropriate repositories, within the body of the manuscript, or as supporting information (N.B. this includes any numerical values that were used to generate graphs, histograms etc.). Please also indicate in each figure legend where the data can be found. For an example see here: http://www.plosbiology.org/article/info%3Adoi%2F10.1371%2Fjournal.pbio.1001908#s5

b) *Published Peer Review*

Please note while forming your response, if your article is accepted, you may have the opportunity to make the peer review history publicly available. The record will include editor decision letters (with reviews) and your responses to reviewer comments. If eligible, we will contact you to opt in or out. Please see here for more details:

https://blogs.plos.org/plos/2019/05/plos-journals-now-open-for-published-peer-review/

c) *Blot and Gel Data Policy*

Please provide the original, uncropped and minimally adjusted images supporting all blot and gel results reported in an article's figures or Supporting Information files. We will require these files before a manuscript can be accepted so please prepare them now, if you have not already uploaded them. Please carefully read our guidelines for how to prepare and upload this data: https://journals.plos.org/plosbiology/s/figures#loc-blot-and-gel-reporting-requirements

d) *Protocols deposition*

To enhance the reproducibility of your results, we recommend that if applicable you deposit your laboratory protocols in protocols.io, where a protocol can be assigned its own identifier (DOI) such that it can be cited independently in the future. For instructions see: https://journals.plos.org/plosbiology/s/submission-guidelines#loc-materials-and-methods

Thank you again for your submission to our journal. We hope that our editorial process has been constructive thus far, and we welcome your feedback at any time. Please don't hesitate to contact us if you have any questions or comments.

Sincerely,

Ines

--

Ines Alvarez-Garcia, PhD

Senior Editor

PLOS Biology

ialvarez-garcia@plos.org

----------------------------------------------------------------

Reviewers’ comments

Rev. 1:

In this manuscript, the authors show that Sxl (unlike the other splicing factors tested) binds to chromatin, mainly near the transcription start sites for both PolII and PolII transcripts. They further show that domains of Sxl required for RNA binding and other aspects of Sxl splicing function are not required for chromatin binding. They show that Sxl binding overlaps with that of Polr3E and is dependent on Polr3E (based on RNAi experiments knocking down Polr3E). Sxl bound about half of the genes in the genome (53% occupancy on expressed genes), including 156 of the 297 tRNAs encoded in Drosophila, whereas Polr3E bound 238 of the 297 tRNA genes. The authors show that knockdown of Sxl in males results in very slight decreases in long term male survival, whereas knockdown of Polr3E had more significant decreases. They show that loss of both genes improves a behavior - negative geotaxis - in the same direction. They show that there is some correlation between gene expression changes in Sxl knock down and knockdown of Polr3E. They show that 50 of the 365 genes affected by Sxl knock down are shared with knockdown of Polr3E (although they do not indicate if the 50 genes change expression in the same direction). They show that overexpression of the form of Sxl normally expressed in male neurons leads to male lethality as well as changes in expression of 31 of the 287 tRNA genes encoded in Drosophila (a class of genes known to be PolIII dependent). They show that 23 of the 31 tRNA genes go up (only 20 are shown in the table) and nine go down. Most of the affected tRNAs are for Lysine (17; one each for Cys, Arg and Tyr, no information is shown for the three not in the table). They show that localization of a tagged version of Sxl is visible in male brains and correlates with the presence of OPP (a measure of translation levels) (Rcoloc = 0.81). Finally, they show that translation (as measured by OPP intensity) goes up when the chromatin binding form of Sxl is overexpressed and translation goes down when Sxl is knocked down by RNAi. Concerningly, the controls in the two different experiments changed as much as the experimentals did in the individual experiments.

Many of my original concerns remain regarding the significance of the findings presented in this paper. The major ones are listed below.

The authors never share whether the same genes change expression in expected ways across experiments. They show only broad correlations. It would be nice to have shown a table of all the genes that significantly change and what direction they change in all experimental conditions.

Why are Polr3E and Sxl binding to PolII transcribed genes as well as to PolIII transcribed genes? Is it possible that these proteins are simply accumulating on open chromatin? How does this binding compare with active chromatin markers?

It's not clear in Figure 4G if the correlation in genes that change in Sxl and PolrE3 knockdown are comparisons of genes that only go down or if they are genes that go in both directions and if the direction of expression change was accounted for in the analysis. What is plotted on the X axis?

Likewise, with the 50 genes whose expression is altered with loss of Sxl and PolrE3 shown in the Venn diagram in Figure 4G, it is unclear if the direction of change is the same in the two different knockdown backgrounds.

The OPP assays to measure levels of translation are also concerning, since the levels of OPP staining change as much between the two different control samples as they change between the control and the experimental in each individual experiment. I think this may indicate that there can be considerable variation from animal to animal, experiment to experiment with this methodology. Importantly, we are never told how many animals were imaged in these experiments nor how many sections were analyzed. Even if I had more confidence in the assay, it is hard to understand why translation is changing in both directions. Translation might be expected to go down if most of the tRNAs for lysine are reduced in expression, but I wouldn't expect translation to go up with so few tRNAs changing expression - translation would require most tRNAs be upregulated.

Minor question:

What are the gray boxes in the top diagram in Figure 3A, 3D?

Rev. 2:

In this revised manuscript, Storer et al. have incorporated additional evidence addressing one of my concerns (whether protein translation was indeed affected in SxlRAC-expressing neurons by SxlRAC) as well as the wider molecular model proposed (the effect of recruiting Sxl by Pol3Er in regulating tRNAs.

My previous stance is the same: I think the tenet of the paper, namely, that Sxl molecular function goes beyond its role as an RNA binding protein to regulate splicing and dose compensation but as a chromatin binding protein recruited by Pol3Er to regulate transcription, is novel and sufficiently backed by the data to be of interest as a paper without adding more. With the new data (the imaging data, at least), the original claim seems even more robust.

Therefore, my recommendation is the same: publication with minor modification/clarification.

My minor comments to this version of the paper are as follows:

1 - I am not very enthusiastic about the new emphasis on Sxl exerting its function through increasing tRNA expression. This is an expansion of the original claim that feels a bit overstretched. There are two things that jar in this model.

+ 1A - One, that there is no evidence that the effects of Sxl happen through changes in tRNA abundance. It is a reasonable speculation, but just that. To address this, it would be necessary to do some epistatic analysis, i.e. over expressing tRNA-Leu-TAT or a tRNA-Lys (or a combination) and observe a rescue of the phenotypes observed with SxlRNAi (lifespan, climbing). That would be a difficult experiment to run satisfactorily because of the redundancy and complexity of tRNA loci. A simpler one would be to show that you can at least can phenocopy the SxlRNAi with tRNA knock-down, but I think it would still be technically challenging for the same reason. I do not mind these experiments not being done, but then this emphasis and some of the statements may not be warranted (i.e. the epigraph title in line 327 "Sxl modulates neuronal translation downstream of tRNA regulation" - without epistasis analysis the claim that tRNA is downstream cannot be made - or tRNAs being mentioned in the title and Lys-tRNA in the abstract).

+ 1B - Two, the observations about regulation of tRNA expression reported in the paper. There are statistically significant effects on multiple tRNAs, most repeatedly Lysine-bearing tRNAs, however:

(1Bi) The effects seem weak: as most log2FC values are below 0.7, which means increases of expression of 60% at most; yes, for some amino acids there might be a compound effect of having multiple tRNAs experiencing this but, is this biochemically enough to expect an increase of ribosomal activity/efficiency? Is there literature that justifies this inference? If not, it is only a reasonable speculation;

(1Bii) Also, Lysine is the amino acid with most tRNAs unregulated but the pre-tRNA qPCR assays are done for a His, Leu and Ile tRNAs, and only one of them (Ile) is statistically significant (unless I have missed something). So the picture of Sxl increasing tRNAs to increase protein synthesis rates seems mechanistically a bit muddled (though the OPP assays seem convincing at face vale, see below). Statements like in lines 295-97 make this a bit confusing: "... a striking enrichment for Lys-tRNAs precursor tRNA levels, in particular pre-tRNATAT, were elevated relative to the Pol II-specific U3 transcript, as measured by qPCR (Fig. S6), with reversed trends in Sxl knockdowns (Fig. S6)." pre-tRNATAT is an isoleucine tRNA !

2 - I appreciate the effort of the authors in performing the imaging experiments I suggested (OPP incorporation in Sxl RNAi and SxlRAC over-expression and co-localisation analysis). However, the methods are not sufficiently clear about how these were performed. In particular:

+ 2A - Co-localisation analysis needs a baseline control of random co-localisation - absolute measures are not evidence enough. I suggest the authors establish a comparison between OPP and DAPI, for instance; that should give a much lower co-localisation factor value. Also, while the authors describe the pipeline of analysis, they do not explain what "enhanced contrast" means in the tables where they provide the data values. In principle, there should be no contrast enhancement or if there is, it should be done with a reproducible algorithm whose details are included in Methods.

+ 2B - Intensity comparisons with histofluorescence need to be carefully explained to be accepted at face value. The authors do not provide information about image acquisition metadata (i.e. laser intensities, pixel dwell, gain, laser warm-up time, etc) nor intra-image and inter-image normalisation. For instance, the data tables have a normalised intensity column but this normalisation procedure is not described, and it should.

# Author comment: Author response to Decision Letter 3

**DOI:** 10.1371/journal.pbio.3003863.r007

7 May 2026

Attachment Submitted filename: Responses to Reviewer Comments R2_1.docx

# Editor report: Decision Letter 3

**DOI:** 10.1371/journal.pbio.3003863.r008

**Authors:** Ines Alvarez-Garcia

5 Jun 2026

Dear Dr Southall,

Thank you for the submission of your revised Research Article entitled "Neuronal protein Sex-lethal modulates tRNA synthesis via the polymerase III subunit Polr3E in male Drosophila neurons" for publication in PLOS Biology. On behalf of my colleagues and the Academic Editor, Richard Benton, I am delighted to let you know that we can in principle accept your manuscript for publication, provided you address any remaining formatting and reporting issues. These will be detailed in an email you should receive within 2-3 business days from our colleagues in the journal operations team; no action is required from you until then. Please note that we will not be able to formally accept your manuscript and schedule it for publication until you have completed any requested changes.

Please take a minute to log into Editorial Manager at http://www.editorialmanager.com/pbiology/, click the "Update My Information" link at the top of the page, and update your user information to ensure an efficient production process.

PRESS

We frequently collaborate with press offices. If your institution or institutions have a press office, please notify them about your upcoming paper at this point, to enable them to help maximise its impact. If the press office is planning to promote your findings, we would be grateful if they could coordinate with biologypress@plos.org. If you have previously opted in to the early version process, we ask that you notify us immediately of any press plans so that we may opt out on your behalf.

We also ask that you take this opportunity to read our Embargo Policy regarding the discussion, promotion and media coverage of work that is yet to be published by PLOS. As your manuscript is not yet published, it is bound by the conditions of our Embargo Policy. Please be aware that this policy is in place both to ensure that any press coverage of your article is fully substantiated and to provide a direct link between such coverage and the published work. For full details of our Embargo Policy, please visit http://www.plos.org/about/media-inquiries/embargo-policy/.

Many congratulations and thanks again for choosing PLOS Biology for publication and supporting Open Access publishing. We look forward to publishing your study.

Sincerely,

Ines

--

Ines Alvarez-Garcia, PhD

Senior Editor

PLOS Biology

ialvarez-garcia@plos.org
